# Reference values for the quality of life after traumatic brain injury questionnaire and its overall scale from the adult German general population

**DOI:** 10.1186/s41687-026-01014-3

**Published:** 2026-02-09

**Authors:** Marina Zeldovich, Anna C. Mayer, Stephan Wüstenhagen, Katrin Cunitz, Nicole von Steinbuechel

**Affiliations:** 1 Faculty of Psychotherapy Science, Sigmund Freud University Vienna, Vienna, Austria; 2 Institute of Psychology, Universität Innsbruck, Innsbruck, Austria; 3 Department of Clinical Neurophysiology, Filadelfia, Dianalund, Denmark; 4 Department of Psychology, Charlotte Fresenius University of Applied Sciences, Cologne, Germany

**Keywords:** Traumatic brain injury, Health-related quality of life, QoLIBRI, QoLIBRI-OS, Reference values, German general population, Community sample

## Abstract

**Purpose:**

One of the central outcomes to which attention should be paid after sustaining a traumatic brain injury (TBI) is health-related quality of life (HRQoL). The present study aims at providing reference values for the Quality of Life after Brain Injury (QoLIBRI) and its overall scale (QoLIBRI-OS) using data from the German general population.

**Methods:**

A total of 3,502 individuals from the general German population completed an online survey containing adapted versions of both instruments designed for individuals without TBI. Psychometric properties, including reliability and validity analyses, were examined. Comparisons of construct assessment were conducted using 353 sex-, age-, and education-matched dyads from the general population sample and individuals after TBI.

**Results:**

Both instruments demonstrated satisfactory psychometric properties in the general population sample. The construct assessment between the general population and the TBI samples was considered sufficiently comparable to derive reference values. A patient is considered to have an impaired HRQoL if their QoLIBRI or QoLIBRI-OS score falls below the 16th percentile of their reference group, which is defined by sex, chronic health condition status, age, or education.

**Conclusion:**

We provided reference values in form of an open access web-based application. Clinicians can use the provided reference values to directly compare the QoLIBRI and QoLIBRI-OS scores of individuals after TBI with those of the general population. This allows them to identify impaired HRQoL domains and develop personalized treatment plans.

**Supplementary Information:**

The online version contains supplementary material available at 10.1186/s41687-026-01014-3.

## Abstract (plain language summary)

Health-related quality of life (HRQoL) is one of the outcomes requiring attention after sustaining a traumatic brain injury (TBI). The Quality of Life after Brain Injury (QoLIBRI) and its short version, the overall scale (QoLIBRI-OS), are reliable patient-reported outcome measures for assessing TBI-specific HRQoL. This study informs clinical decision-making for individuals after TBI in Germany by providing reference values from the general population, which allows for the easy evaluation and interpretation of questionnaire scores and helps identify areas in need of treatment. The reference values are presented as percentiles, stratified by sex, age, and education, and are available in the form of an open-access web application. With this application, clinicians can directly compare the QoLIBRI/QoLIBRI-OS scores of affected individuals with those of the general population.

**Supplementary Information:**

The online version contains supplementary material available at 10.1186/s41687-026-01014-3.

## Introduction

Traumatic brain injury (TBI) is one of the most common head injuries, with a crude annual incidence in Europe ranging from 47.3 to 694 per 100,000 population on national level [1]. According to multiple studies, the incidence of TBI in Germany consistently aligns with the range of 250–350 per 100,000 [1, 2]. Although mild TBI is the most commonly diagnosed severity and usually requires no hospitalization, hospitalization rates for moderate to severe TBI in Germany are estimated at 10.1 to 13.6 per 100,000, resulting in up to 23.5% mortality cases [3]. A large-scale prospective observational cohort study in Germany found that men in their early twenties and mid-fifties, as well as both sexes in their late seventies, are most frequently affected by TBI [2]. Of those interviewed 12 months after injury, one-third still reported TBI-related problems, despite most commonly having experienced a mild TBI [2]. Overall, TBI places a significant burden on those affected, their families, and healthcare systems.

Apart from its short- and long-term consequences affecting multiple areas of mental and physical health [4], TBI also impacts health-related quality of life (HRQoL) [5]. HRQoL is a multidimensional construct covering various health areas (e.g., physical or emotional). In contrast, disease-specific HRQoL specifically aligns with areas affected by a certain health condition. One of the most commonly used patient-reported outcome measures (PROMs) to assess TBI-related HRQoL is the Quality of Life after Brain Injury (QoLIBRI) questionnaire [6, 7]. The QoLIBRI assesses HRQoL after TBI across six dimensions: cognition, self, daily life and autonomy, social relationships, emotional problems, and physical problems. Its short overall scale (QoLIBRI-OS) [8] allows for a quick, economical evaluation of HRQoL after TBI. Both questionnaires are available in multiple languages [6, 8–13], including German [6, 8, 9, 14], and have been shown to sensitively capture HRQoL cross-sectionally [15] and longitudinally [16].

The interpretation of PROM scores often involves using cut-off values established by different methods. For instance, the cut-off values for the total scores of the both questionnaires were derived using geometric mean regression analysis using the norm-based mental component summary score (MCS) of the Short Form 36 (SF-36) [17]. As a result, values below 60 (QoLIBRI) and 52 (QoLIBRI-OS), respectively, indicate impaired HRQoL [18]. However, such a derivation may neglect other factors affecting HRQoL, such as age, gender and/or sex, or the presence of chronic health conditions. Additionally, it does not provide information on normative HRQoL in the general population.

To overcome this limitation, reference values for the QoLIBRI instruments, based on percentiles from the general population, have recently been established in Italy [19, 20] and in the Netherlands and the United Kingdom [20, 21]. These values provide an indication of whether a patient’s score is lower than would be expected for members of the general population with similar characteristics.

Until now, no reference values have been provided for the German versions of the QoLIBRI or QoLIBRI-OS. Considering that reference values facilitate evaluating the impact of TBI on HRQoL and provide a more nuanced basis for comparing patient scores, the present study aims to close this gap. Before establishing reference values based on data from the German general population, we will analyze the instruments’ applicability to this target group, investigate its psychometric properties, and compare construct assessment between the general population and TBI samples. Once established, the reference values can be used in clinical research and practice to evaluate TBI-specific HRQoL in Germany.

## Methods and materials

### Study participants

Between December 10 and December 23, 2021, participants from the *German general population* completed an online survey. Dynata Ltd (ISO certification 20252) organized the recruitment process and hosted and conducted the survey. The aim was to assess data from *N* = 3,500 participants, selected to be representative of the German general population in terms of sex and age. The target number of participants was determined by ensuring there was a sufficient number to provide reference values, taking into account potential stratifications, and to enable comparison with studies on reference values from other countries [19–21]. Potential participants were recruited through various channels, including generic emails, push notifications, and app notifications. After receiving a brief description of the study, participants could provide informed consent and continue with the survey. Upon completion, they were rewarded with certificates and tokens. Participants who had sustained a TBI within the past ten years or who were suffering from a life-threatening disease were excluded. Further quality checks concerned the quality of open-ended questions, response patterns, and interview completion time. A total of *N* = 3,502 participants were eligible for inclusion in the analyses.

**Fig. 1:**
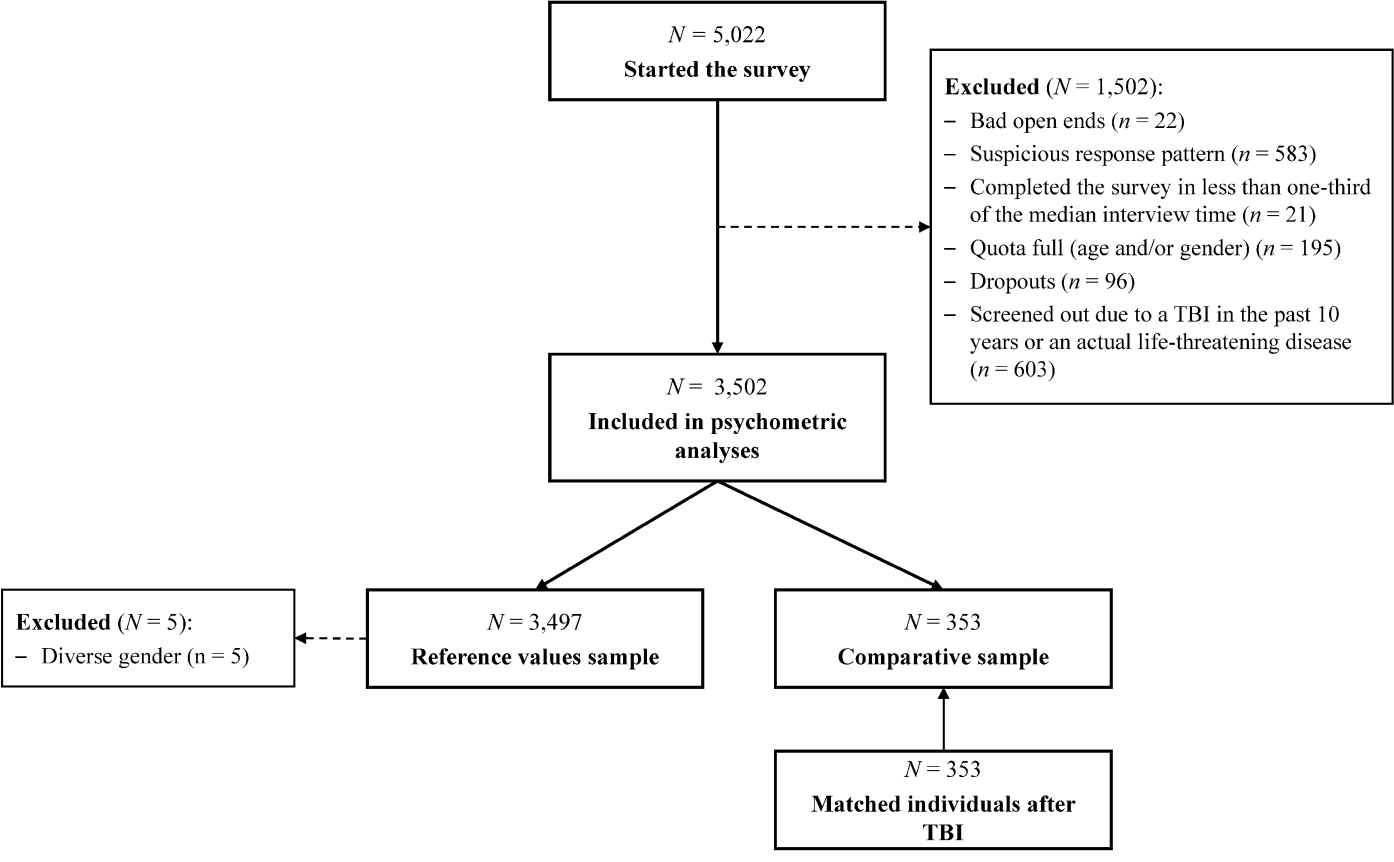
Participants flow

For the comparative analyses of HRQoL assessment, data from *individuals after TBI* were obtained from two sources. The first source was data from German-speaking participants in the QoLIBRI project [6–8]. The second source was data collected at German-speaking sites in the Collaborative European NeuroTrauma Effectiveness Research in Traumatic Brain Injury (CENTER-TBI) project [9, 22]. To ensure accurate comparisons, individuals after TBI were paired via propensity score matching, adjusting for sex, age, and education. A total of *N* = 353 participants were included in the analyses. For details, see Fig. 1.

### Materials

The first part of the online survey assessed sociodemographic (sex, age, education) and health-related (information on chronic health conditions) questions. The latter consisted of the following categories and an open-ended option: asthma, chronic bronchitis, severe heart disease, stroke complications, diabetes, severe back pain, osteoarthritis, rheumatism, cancer, memory impairment due to neurological disease or dementia, age-related memory impairment, and depression. Additionally, participants could indicate that they did not suffer from any chronic health conditions.

The second part of the survey included 37 QoLIBRI items and six QoLIBRI-OS items. To apply the questionnaires to the general population, all content related to TBI was omitted. This concerned the instructions of both questionnaires and the item 37 (“Overall, how bothered are you by the effects of your brain injury?”) of the long version of the QoLIBRI. The instructions were rephrased as follows: “…we would like to know how satisfied (bothered) you are with different aspects of your life”. Item 37 was modified to read: “…disturbed overall by health problems?”. This procedure corresponded to adaptations of the questionnaires for studies conducted in other countries [19–21].

Both questionnaires utilize a five-point Likert-type scale (1: not at all; 2: slightly; 3: moderately; 4: quite; 5: very). To calculate the QoLIBRI scale and total scores, the items on the Emotions and Physical Problems scales are to be reversed. The scales are calculated as mean scores across the items, transformed to a range of 0 (worst HRQoL) to 100 (best HRQoL), according to the scoring procedure. The QoLIBRI-OS score is calculated similarly by averaging the item values, transformed to a 0–100 range.

Participants after TBI responded to the initial self-report versions of both questionnaires in German language.

### Statistical analyses

We examined item characteristics (response patterns, *M*, *SD*, skewness, kurtosis, floor and ceiling effects), reliability, unidimensionality measures, and factorial validity of both questionnaires to ensure their applicability to the general German population.

Reliability analyses included the following metrics (acceptable values in parentheses): Cronbach’s alpha (α > 0.70) [23], α if item omitted (no exceeding initial α of the scale), corrected item-total correlations (CITC > 0.40) [24], and McDonald’s omega (ω > 0.70). The unidimensionality measures comprised assessment of tau equivalence (τ), congeneric fit (ρ_c_), and their product (*U*) to indicate unidimensionality and homogeneity of the scales [25]. Values closer to 1 were considered desirable [25]. These measures additionally inform reliability findings obtained using Cronbach’s α and McDonald’s ω.

We tested the applicability of the six-factor model to the QoLIBRI data and the one-factor model to the QoLIBRI-OS data using confirmatory factor analysis (CFA) with both a robust maximum likelihood (MLR) estimator and a robust mean and variance-adjusted weighted least squares (WLS) estimator. The following indices were used to evaluate the model fit (acceptable values in parentheses): χ^2^ with respective *p* value (*p* > 0.05), comparative fit index (CFI > 0.90), Tucker-Lewis index (TLI > 0.90), root mean square error of approximation (RMSEA ≤ 0.08) with 90% confidence interval (CI), and standardized root mean square residual (SRMR ≤ 0.10) [26].

Then, we conducted measurement invariance (MI) analyses based on MLR estimator to compare HRQoL assessment between the general population and the TBI samples. Prior to this, we applied a matching procedure to adjust for age, sex, and level of education. We used the nearest neighbor approach based on propensity scores estimated via logistic regression analyses. This approach was designed to distinguish more clearly the impact of belonging to the general population versus the TBI population on HRQoL assessment. We used a Love plot [27, 28] to visualise the covariance balance between the matched samples. A perfect balance for each matched characteristic is indicated by a value of 1. In the matched sample, we tested and compared three increasingly constrained models (equality of factor loadings, intercepts, and residuals). The MI assumption can be retained if the differences between the models are not significant (*p* > 0.05). Additionally, we deemed changes in CFI and RMSEA negligible if they were less than ΔCFI ≤ 0.010 [29] and ΔRMSEA ≤ 0.015 [30], respectively, to inform the decision regarding non-violation of MI.

Prior to establishing reference values, we carried out regression analyses with QoLIBRI(−OS) scale and total scores as outcomes and sex (male and female; *n* = 5 diverse participants were omitted due to low number of cases), age in groups (young adults: 18 to 40 years; middle-aged adults: 41 to 64 years; and older adults: over 65 years), and presence of chronic health conditions (no and yes; coded as present, if any of listed conditions was endorsed) as covariates. Additionally, we inspected interactions between the factors to determine further stratification of the reference values.

Finally, we established reference values using the following percentiles: 2.5%, 5%, 16%, 30%, 40%, 50%, 60%, 70%, 85%, 95% and 97.5%. The 50^th^ percentile corresponds to the median and mean of the distribution. The average HRQoL value falls within the 16–85% range (both cut-offs rounded to the next integer). Therefore, values corresponding to the 16^th^ percentile or below indicate impairment in HRQoL and are considered clinically relevant.

We used R version 4.3.3 [31] applying the following packages: *table1* [32] for descriptives, *psych* [33] for psychometric analyses, and *MBESS* [34] for reliability coefficients including 95% CI, *matchIt* [35] for propensity score matching, *lavaan* [36], *semTools* [37] and *semPlot* [38] for CFA and MI, and *car* [39] for regression analyses. All analyses employed a 5% alpha level to determine significance.

**Table 1:** Sample characteristics of the German general population sample

Characteristics	Group/Value	*N* **(%)**
Sex	Female	1,777 (50.7)
	Male	1,720 (49.1)
	Diverse	5 (0.1)
Age in years	*M* (*SD*)	49.7 (16.8)
	*Mdn* [Min, Max]	51 [18 88]
Age in groups^1^	Young Adults	1,143 (32.6)
	Middle-aged Adults	1,482 (42.3)
	Older Adults	877 (25.0)
Education^2^	Low Education	293 (23.5)
	Medium Education	2,386 (68.1)
	High Education	823 (23.5)
Chronic health conditions^3^	No	1,596 (45.6)
	Yes	1,906 (54.4)

^1^Young Adults: 18 to 40 years, Middle-aged Adults: 41 to 64 years, Older Adults: over 65 years

^2^ Low Education: no degree or primary school, Medium Education: secondary school, high school, vocational school or trade or technical certificate; High Education: university or college degree.

^3^Coded as present, if any of listed conditions (i.e., asthma, chronic bronchitis, severe heart disease, stroke complications, diabetes, severe back pain, osteoarthritis, rheumatism, cancer, memory impairment due to neurological disease or dementia, age-related memory impairment, or depression) was endorsed

*Note*. *N*: absolute frequencies. %: relative frequencies. *M*: mean. *SD*: standard deviation. *Mdn*: median. *Min*: minimum. *Max*: maximum.

## Results

### Sample characteristics

A total of 3,502 participants (51% female) with an average age of 49.7 ± 16.8 years were included in the final analyses. Most participants (68%) had graduated from secondary school, high school, or a vocational school, or they had a trade or technical certificate. More than half (54%) reported not suffering from any chronic health conditions (see Table 1).

### Psychometric properties

#### Item characteristics

For the QoLIBRI items, average item responses were *M* = 3.65, *SD* = 1.08 with moderate levels of skewness and approximately normal-tailed distribution ($\overline {SK} = - 0.63, \overline {KU} = - 0.05$). For the QoLIBRI-OS items, average item responses were *M* = 3.46, *SD* = 1.08 ($\overline {SK} = - 0.64, \overline {KU} = 0.11)$. Overall, the responses indicated moderate (3) to quite (4) levels of satisfaction with HRQoL. This was also reflected in the response patterns, with most participants reporting moderate to high satisfaction across all dimensions. For details, see Appendix, Table A1.

#### Reliability

Reliability values ranged from α = 0.82, 95% CI[0.81, 0.84] (Social) to α = 0.94, 95% CI [0.94, 0.95] (QoLIBRI Total Score) and from ω = 0.82, 95% CI [0.81, 0.83] (Social) to ω = 0.94, 95% CI [0.94, 0.94] (QoLIBRI Total Score). The CIs were narrow. None of the items contributed to the initial internal consistency inflation of the scales. CITC exceeded 0.40 across all domains. Unidimensionality was close to 1 for all domains and the QoLIBRI-OS Total Score but decreased for the QoLIBRI Total Score. For details, see Appendix Table A2.

#### Factorial validity

**Fig. 2:**
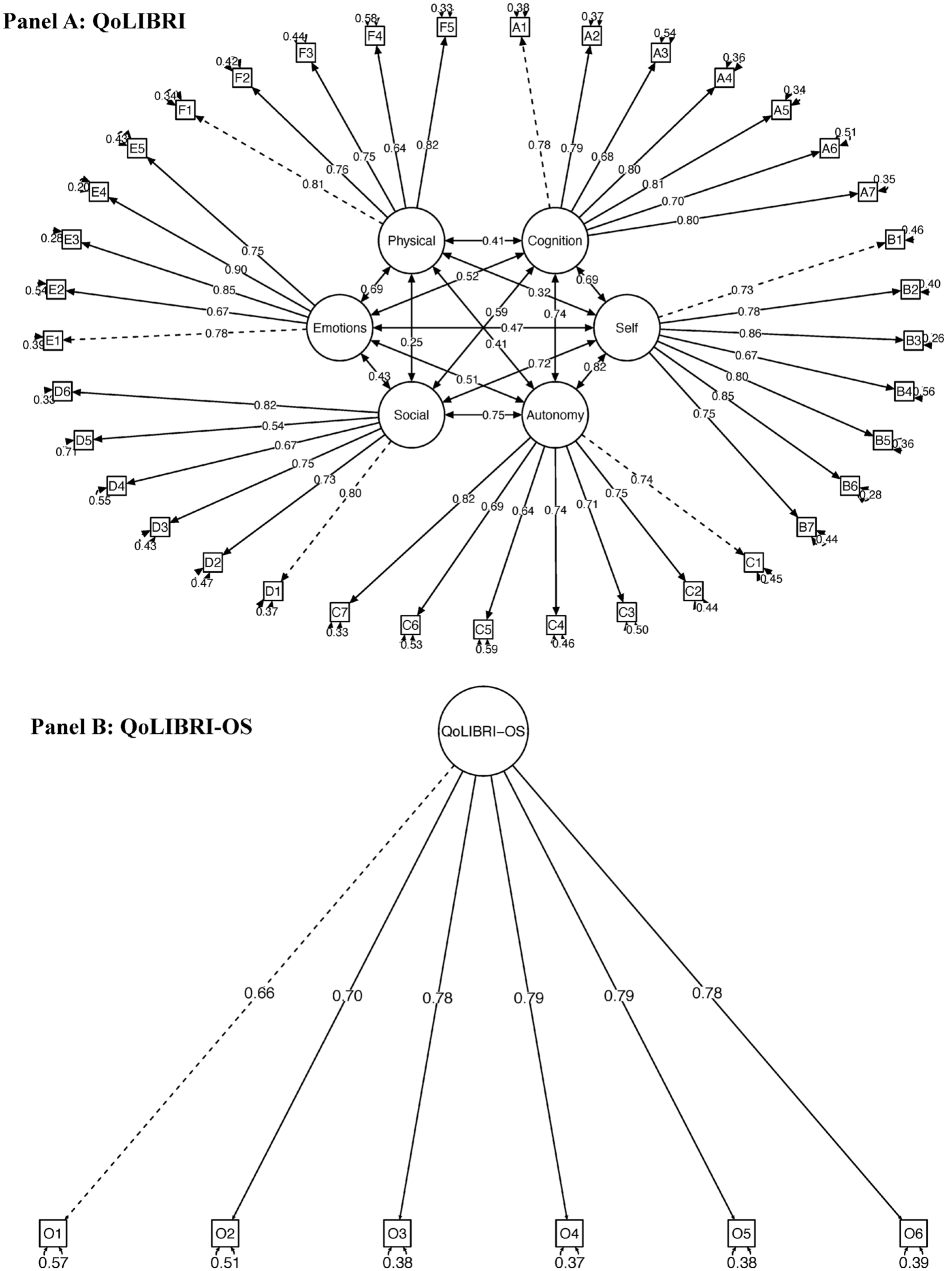
CFA results (standardized coefficients). The six-factor structure of the QoLIBRI (panel **A**) comprises the following latent factors: Cognition, Self, Autonomy and Daily life, Social Relationships, Emotional Problems, and Physical Problems. The one-factor structure of the QoLIBRI-OS (panel **B**) comprises the overall HRQoL: the QoLIBRI-OS total score

The six-factor structure of the QoLIBRI showed a satisfactory model fit, with almost all of the fit indices being within the permissible cut-off ranges irrespective of the chosen estimator. The unidimensional structure of the QoLIBRI-OS also showed a satisfactory model fit, except for the RMSEA, which was slightly elevated in both tested models. For details, see Table 2 and Fig. 2 and Tables S1–S10.

**Table 2:** Goodness of model fit for the QoLIBRI and QoLIBRI-OS

Scale	Estimator	**χ**^**2**^** (df)**^**1**^	*p*^**1**^	**CFI**^**1**^	**TLI**^**1**^	**RMSEA [90% CI]** ^**1**^	**SRMR**^**2**^	**AIC**^**3**^	**BIC**^**3**^
QoLIBRI	WLS	9,209.279 (614)	< 0.001	0.880	0.870	0.069 [0.067, 0.070]	0.046	–	–
	MLR	5,477.144 (614)	< 0.001	0.904	0.896	0.055 [0.053, 0.056]	0.044	324,546.60	325,094.94
QoLIBRI-OS	WLS	440.362 (9)	< 0.001	0.961	0.934	0.113 [0.104, 0.123]	0.034	–	–
	MLR	193.253 (9)	< 0.001	0.967	0.944	0.095 [0.083, 0.107]	0.032	51,010.92	51,084.85

^1^For WLS estimators, robust values are reported for χ^2^, CFI, TLI, RMSEA, and CI; for MLR estimators, scaled values are reported for χ^2^, robust CFI, TLI, and RMSEA and CI

^2^Standard SRMR is reported for all estimators

^3^AIC and BIC are provided for MLR only

*Note*. WLS: mean and variance-adjusted weighted least square estimator, MLR: robust maximum likelihood estimator, *df*: degrees of freedom, *p*: *p*-value, CFI: comparative fit index, TLI: Tucker-Lewis index, RMSEA: root mean square error of approximation with 90% confidence interval (CI), AIC: Akaike’s information criterion, BIC: Bayesian information criterion

### Reference values

#### Measurement invariance

Matching resulted in *N* = 353 dyads from the general and TBI samples, with a variance ratio close to 1. This indicates that the samples were successfully aligned according to the sociodemographic characteristics (see Fig. S1 and Table S11). The measurement invariance analyses revealed significant differences (*p* < 0.05) between the models with increasing constraints among the investigated groups (see Table S12 for an overview, and Tables S13–S32 for further details). However, as the differences in fit indices were most frequently found to be at the second decimal place, they were not necessarily practical. Therefore, we concluded that the construct assessment was sufficiently comparable across the samples, and we deemed it appropriate to provide the reference values obtained from the German general population sample.

#### Regression analyses

Higher age, a higher level of educational attainment, and an absence of chronic health conditions were found to be indicative of better HRQoL, as measured by the QoLIBRI and the QoLIBRI-OS Total Scores (see Table S33). Additionally, there were sex-related effects on the Self (Table S37) and Emotion (Table S42) scales. The significant results of the second-order interactions indicated that the reference values should be classified according to sex and the presence of chronic health conditions, and then further divided into age groups and educational levels, in line with previous studies on both instruments [19–21]. For details on results, see Tables S34–S47.

#### Reference values

According to the percentile-based reference values, a patient is considered to experience an impaired HRQoL if their QoLIBRI or QoLIBRI-OS score falls below the 16^th^ percentile of the reference group to which they belong, as defined by their sex, chronic health condition status, and age or education. Table A3–Table A6 contain reference values for the QoLIBRI and QoLIBRI-OS Total Scores. A detailed overview on reference values for the scales and total scores is available at https://mzeldovich.shinyapps.io/Reference_Values_QOLIBRI_German/ (last access 15^th^ October 2025). Below two examples are given on how to interpret reference values in the clinical practice:

##### Example 1: QoLIBRI total score

After a TBI, a middle-aged woman (ages 40–59) with a chronic health condition reports a QoLIBRI Total Score of 45. The mean score in the reference group of middle-aged women with chronic conditions is 63.5 ± 16.4. The 16^th^ percentile is 47. A score of 45 is below the 16^th^ percentile, indicating impaired HRQoL compared to general population.

##### Example 2: QoLIBRI-OS

After a TBI, a young adult man (ages 18–39) with a chronic health condition reports a QoLIBRI-OS score of 40. In the reference group of young adult men with chronic conditions, the mean score is 56.3 ± 18.8. The 16^th^ percentile is 42. A score of 42 is below the 16^th^ percentile, indicating impaired HRQoL compared to general population.

## Discussion

The present study aimed to provide reference values for the QoLIBRI and its short form the QoLIBRI-OS based on data from a large, sex- and age-representative sample of the German general population. Prior to providing these values, we examined the psychometric properties of both instruments and compared HRQoL assessment to the assessment in the TBI sample for which the instruments were initially designed. The reference values are available as an open-access web application that allows users to easily assess and apply the values when evaluating the HRQoL of individual patients.

Overall, reference data from the German population closely align with international studies, replicating similar stratification schemes, socio-demographic patterns, and cut-off points for impaired HRQoL [19–21]. Compared to other countries, the general population in Germany reports similar or slightly higher HRQoL levels than in Italy [19, 20], notably higher levels than in the UK [20, 21], and slightly lower levels than in the Netherlands [20, 21]. While these cross-country comparisons are descriptive, they offer preliminary insights into quality of life in different countries. They also underscore the importance of establishing more nuanced criteria instead of relying solely on common, unified cut-off values, as previously suggested [18].

We also find similar patterns when comparing the HRQoL findings collected using the QoLIBRI instruments with the findings from other studies that used generic measures (e.g., SF-36 or EQ-5D-5 L). In an international comparison, the German general population reports relatively high generic HRQoL. The presence of chronic health conditions also has a pronounced effect on quality of life, resulting in lower questionnaire scores [40, 41]. The same applies to lower educational attainment [42]. However, the positive effect of age on HRQoL observed in this study is not fully supported by previous research. This discrepancy may be partly attributable to the composition of the groups and the sample size of particular age groups (e.g., older adults) [41].

Taken together, the results of the present study indicate the applicability of slightly adapted QoLIBRI instruments to the German general population. The results also provide a basis for within- and cross-country comparisons, thereby contributing to the national and international HRQoL research.

### Study limitations

Although this is the first study to provide general population-based reference values for the QoLIBRI instruments in Germany, there are some limitations to disclose. First, the survey was conducted online, which limited our ability to control the setting in which the questionnaires were completed. We attempted to overcome this limitation by collecting data through a research agency and controlling for data quality. Second, the data from the German general population were designed to be representative of sex and age; however, neither their intersections nor other factors were representative, thus limiting the generalizability of the findings. Finally, health condition information was based on self-report and not collected using diagnoses. Nevertheless, given the comparability with results from other studies on HRQoL, we assume the reliability of the results obtained in the present study.

## Conclusion

The reference values provided in this study can now be used in German clinical practice and national and international research. The study findings underscore the necessity of more nuanced normative data that does not solely rely on the cut-offs of the questionnaire’s scales and total scores. The web-based, open-access application facilitates the application of reference values and allows single patients to be compared to the general population while considering sociodemographic and health-related factors. This, in turn, enables more precise identification of areas of impairment and adaptation of treatment to individual needs.

## Electronic supplementary material

Below is the link to the electronic supplementary material.

Supplementary Material 1

## Data Availability

The data that support the findings of this study are available on request from the corresponding author. The data are not publicly available due to privacy or ethical restrictions.

## Appendix A

**Table A1:** Descriptive statistics of the QoLIBRI and QoLIBRI-OS items

								Responses				
Scale	Item	M	SD	SK	KU	Floor	Ceiling	1	2	3	4	5
Cognition	A1	3.7	0.96	−0.74	0.42	0.03	0.18	3%	8%	24%	47%	18%
	A2	3.88	0.96	−0.79	0.33	0.02	0.28	2%	7%	20%	43%	28%
	A3	3.72	0.94	−0.63	0.28	0.03	0.2	3%	7%	27%	44%	20%
	A4	3.95	0.93	−0.83	0.53	0.02	0.3	2%	5%	19%	43%	30%
	A5	3.91	0.98	−0.83	0.44	0.03	0.31	3%	5%	21%	40%	31%
	A6	4.12	0.99	−1.09	0.76	0.02	0.44	2%	5%	16%	33%	44%
	A7	3.83	0.91	−0.71	0.54	0.02	0.23	2%	5%	24%	46%	23%
Self	B1	3.29	0.98	−0.52	0.08	0.06	0.08	6%	10%	39%	37%	8%
	B2	3.31	1.01	−0.46	−0.12	0.06	0.1	6%	12%	36%	36%	10%
	B3	3.54	1.11	−0.58	−0.27	0.06	0.2	6%	11%	26%	37%	20%
	B4	3.35	0.96	−0.56	0.12	0.05	0.08	5%	11%	36%	40%	8%
	B5	3.43	1.05	−0.59	−0.05	0.07	0.13	7%	10%	31%	40%	13%
	B6	3.44	0.98	−0.64	0.18	0.05	0.1	5%	10%	31%	44%	10%
	B7	3.25	1.05	−0.48	−0.24	0.08	0.09	8%	12%	35%	36%	9%
Daily Life and Autonomy	C1	3.69	1.05	−0.66	0	0.04	0.23	4%	8%	25%	39%	23%
	C2	3.81	1.09	−0.76	−0.03	0.04	0.31	4%	8%	22%	35%	31%
	C3	3.83	1.01	−0.71	0.09	0.03	0.28	3%	7%	23%	39%	28%
	C4	3.96	0.99	−0.82	0.22	0.02	0.35	2%	6%	20%	37%	35%
	C5	3.45	1.25	−0.62	−0.53	0.12	0.21	12%	8%	24%	35%	21%
	C6	3.33	1.14	−0.44	−0.48	0.09	0.15	9%	12%	30%	34%	15%
	C7	3.66	1.02	−0.6	−0.03	0.04	0.21	4%	9%	26%	40%	21%
Social Relationships	D1	3.84	1	−0.73	0.16	0.03	0.29	3%	7%	23%	39%	29%
	D2	3.79	1.06	−0.75	0.09	0.04	0.28	4%	7%	23%	37%	28%
	D3	3.65	1.03	−0.65	0.09	0.04	0.21	4%	8%	27%	40%	21%
	D4	3.67	1.22	−0.76	−0.26	0.09	0.3	9%	7%	22%	33%	30%
	D5	3.12	1.28	−0.31	−0.9	0.17	0.14	17%	10%	30%	28%	14%
	D6	3.54	0.92	−0.62	0.37	0.03	0.12	3%	8%	31%	46%	12%
Emotional Problems^1^	E1	3.8	1.2	−0.56	−0.82	0.04	0.41	4%	12%	24%	19%	41%
	E2	3.8	1.19	−0.63	−0.66	0.04	0.38	4%	12%	22%	24%	38%
	E3	3.72	1.26	−0.63	−0.72	0.07	0.37	7%	13%	20%	24%	37%
	E4	3.62	1.28	−0.51	−0.87	0.07	0.34	7%	14%	21%	24%	34%
	E5	3.85	1.17	−0.7	−0.5	0.04	0.39	4%	10%	22%	24%	40%
Physical Problems^1^	F1	3.81	1.17	−0.59	−0.68	0.04	0.38	4%	12%	24%	23%	38%
	F2	3.88	1.18	−0.7	−0.6	0.03	0.42	3%	11%	21%	22%	42%
	F3	3.38	1.25	−0.31	−0.95	0.08	0.23	8%	18%	23%	28%	23%
	F4	3.75	1.15	−0.6	−0.55	0.04	0.33	4%	12%	22%	30%	33%
	F5	3.35	1.1	−0.34	−0.6	0.06	0.15	6%	16%	29%	34%	15%
QoLIBRI-OS	O1	3.2	0.95	−0.61	0.06	0.07	0.04	7%	12%	40%	37%	4%
	O2	3.67	0.96	−0.72	0.36	0.03	0.17	3%	8%	25%	47%	17%
	O3	3.43	0.96	−0.64	0.21	0.05	0.1	5%	10%	32%	44%	10%
	O4	3.76	1	−0.74	0.25	0.03	0.24	3%	7%	23%	43%	24%
	O5	3.47	1.01	−0.57	0	0.05	0.13	5%	10%	30%	41%	13%
	O6	3.25	1.05	−0.53	−0.2	0.09	0.08	9%	11%	34%	37%	8%

*Note*. ^1^ reversed items,*M*: mean, *SD*: standard deviation, *SK*: Skewness, *KU*: Kurtosis, Floor: floor effect (percentage of participants endorsing the lowest response scale category), Ceiling: ceiling effect (percentage of participants endorsing the highest response scale category), Responses: endorsement of response categories in % (1: 1: not at all; 2: slightly; 3: moderately; 4: quite; 5: very)

**Table A2:** Item and scale characteristics of QoLIBRI and QoLIBRI-OS items

Scale	Item	α [95% CI]	α if item deleted	ω [95% CI]	τ	**ρ**_**c**_	U	CITC
Cognition	A1	0.88 [0.87, 0.89]	0.87	0.88 [0.87, 0.89]	0.99	< .99	0.98	0.72
	A2		0.86					0.75
	A3		0.87					0.67
	A4		0.86					0.76
	A5		0.87					0.71
	A6		0.87					0.64
	A7		0.86					0.76
Self	B1	0.89 [0.89, 0.90]	0.89	0.89 [0.87,0.90]	0.98	0.99	0.97	0.67
	B2		0.88					0.72
	B3		0.87					0.79
	B4		0.88					0.67
	B5		0.87					0.76
	B6		0.87					0.83
	B7		0.88					0.71
Daily Life and Autonomy	C1	0.85 [0.84, 0.86]	0.83	0.85 [0.85, 0.86]	0.98	0.99	0.97	0.69
	C2		0.83					0.73
	C3		0.84					0.66
	C4		0.84					0.61
	C5		0.85					0.58
	C6		0.84					0.65
	C7		0.82					0.78
Social Relationships	D1	0.82 [0.81, 0.84]	0.79	0.82 [0.81, 0.83]	0.97	0.98	0.96	0.72
	D2		0.8					0.68
	D3		0.8					0.68
	D4		0.8					0.68
	D5		0.82					0.57
	D6		0.8					0.66
Emotional Problems	E1	0.86 [0.86, 0.87]	0.83	0.87 [0.86, 0.87]	0.98	0.99	0.98	0.73
	E2		0.85					0.67
	E3		0.83					0.78
	E4		0.82					0.81
	E5		0.84					0.69
Physical Problems	F1	0.84 [0.83, 0.85]	0.8	0.84 [0.83, 0.85]	0.99	0.99	0.98	0.72
	F2		0.8					0.72
	F3		0.8					0.71
	F4		0.83					0.61
	F5		0.8					0.75
QoLIBRI Total Score	A1–F5	0.94 [0.94, 0.95]	–	0.94 [0.94, 0.94]	0.86	0.91	0.78	–
QoLIBRI-OS	O1	0.86 [0.85, 0.87]	0.85	0.86 [0.85, 0.87]	0.99	0.99	0.98	0.62
	O2		0.85					0.65
	O3		0.83					0.74
	O4		0.83					0.75
	O5		0.83					0.73
	O6		0.83					0.74
QoLIBRI-OS Total Score	O1–O6	0.86 [0.85, 0.87]	–	0.86 [0.85, 0.87]	0.99	0.99	0.98	–

*Note*. α: Cronbach’s alpha, ω: McDonald’s omega, CI: 95% confidence interval limits are calculated using percentile bootstrap with 1000 replications, τ: tau equivalence, ρ_c_: congeneric fit, U: product of τ and ρ_c_, CITC: corrected item-total correlations

**Table A3:** Reference values for QoLIBRI total score by age

Sex	**Chronic Health Condition** ^**1**^	**Age** ^**2**^	N	M	SD	2.5%	5%	16%	30%	40%	50%	60%	70%	85%	95%	97.5%
Female	No	Young Adults	397	61.72	13.44	38	42	48	53	57	61	65	70	77	84	87
		Middle-aged Adults	309	70.34	14.54	40	47	53	63	67	71	77	81	87	90	92
		Older Adults	102	77.32	13.42	47	51	66	75	78	81	82	84	87	92	95
Female	Yes	Young Adults	317	56.48	12.66	31	35	46	50	52	56	59	63	70	78	81
		Middle-aged Adults	412	63.47	16.39	30	35	47	54	60	64	69	74	81	87	90
		Older Adults	240	71.49	14.23	36	43	58	66	71	75	78	81	84	90	91
Male	No	Young Adults	250	63.77	13.87	41	44	49	55	59	63	67	72	80	86	91
		Middle-aged Adults	371	71.16	14.19	43	47	54	64	70	73	77	81	86	91	93
		Older Adults	165	78.87	10.53	56	58	68	74	78	81	83	86	89	92	93
Male	Yes	Young Adults	178	57.43	12.43	35	40	48	51	53	56	59	62	72	80	83
		Middle-aged Adults	389	63.34	17.06	27	34	47	54	59	64	70	75	81	89	91
		Older Adults	367	71.82	12.62	41	50	60	67	70	74	76	80	84	90	91

^1^Coded present, if any of listed conditions (i.e., asthma, chronic bronchitis, severe heart disease, stroke complications, diabetes, severe back pain, osteoarthritis, rheumatism, cancer, memory impairment due to neurological disease or dementia, age-related memory impairment, and depression) was endorsed.

^2^Young Adults: 18 to 40 years, Middle-aged Adults: 41 to 64 years, Older Adults: over 65 years. 16^th^ percentile represents the cut-off for impaired HRQoL

*Note. N*: absolute frequencies. %: percentiles. M: mean. SD: standard deviation.

**Table A4:** Reference values for QoLIBRI total score by Education

Sex	**Chronic Health Condition** ^**1**^	**Education** ^**2**^	N	M	SD	2.5%	5%	16%	30%	40%	50%	60%	70%	85%	95%	97.5%
Female	No	Low Education	56	64.08	15.47	35	42	49	52	60	63	68	74	82	89	91
		Medium Education	560	66.36	15.16	39	43	50	57	63	67	71	77	83	89	91
		High Education	192	69.68	13.83	45	48	54	60	66	72	75	80	84	89	92
Female	Yes	Low Education	95	59.11	16.85	28	32	42	50	54	58	65	70	77	85	87
		Medium Education	725	63.28	15.77	32	36	48	54	59	63	68	74	81	87	90
		High Education	149	65.2	14.55	38	43	50	56	61	66	70	75	80	88	91
Male	No	Low Education	60	61.85	15.89	37	42	48	50	53	58	62	71	82	87	91
		Medium Education	484	69.81	14.2	43	45	53	62	67	72	75	79	85	91	92
		High Education	242	73.78	13.5	47	49	59	67	72	76	80	83	87	92	93
Male	Yes	Low Education	81	61.48	16.38	26	30	48	52	56	61	67	71	81	85	87
		Medium Education	614	65.1	15.66	33	37	49	57	61	66	71	75	82	89	90
		High Education	239	68.08	14.76	37	41	53	61	66	70	74	78	83	89	91

^1^Coded as present, if any of listed conditions (i.e., asthma, chronic bronchitis, severe heart disease, stroke complications, diabetes, severe back pain, osteoarthritis, rheumatism, cancer, memory impairment due to neurological disease or dementia, age-related memory impairment, and depression) was endorsed.

^2^Low Education: no degree or primary school, Medium Education: secondary school, high school, vocational school or trade or technical certificate; High Education: university or college degree. 16^th^ percentile represents the cut-off for impaired HRQoL

*Note*. *N*: absolute frequencies. %: percentiles. *M*: mean. *SD*: standard deviation.

**Table A5:** Reference values for QoLIBRI-OS by age

Sex	**Chronic Health Condition** ^**1**^	**Age** ^**2**^	N	M	SD	2.5%	5%	16%	30%	40%	50%	60%	70%	85%	95%	97.5%
Female	No	Young Adults	397	61.69	17.7	25	29	46	50	54	62	71	75	79	88	92
		Middle-aged Adults	309	65.91	17.24	25	33	50	58	62	71	75	75	83	92	92
		Older Adults	102	71	16.52	36	42	55	71	71	75	75	79	87	92	96
Female	Yes	Young Adults	317	56.74	18.41	17	25	38	50	50	58	62	67	75	83	96
		Middle-aged Adults	412	56.24	20.29	14	21	33	46	54	58	62	71	75	88	88
		Older Adults	240	63.56	18.12	21	25	50	58	62	67	71	75	79	84	92
Male	No	Young Adults	250	62.3	19.59	19	27	46	54	58	62	71	75	83	92	95
		Middle-aged Adults	371	66.31	18.04	21	29	50	62	67	71	75	75	83	88	92
		Older Adults	165	72.9	13.87	38	50	58	67	71	75	79	82	88	92	95
Male	Yes	Young Adults	178	56.34	18.8	12	21	42	50	53	58	62	67	75	83	88
		Middle-aged Adults	389	55.42	20.48	12	18	33	46	50	54	62	71	75	88	88
		Older Adults	367	63.01	17.02	21	26	46	54	62	67	71	75	79	86	88

^1^Coded as present, if any of listed conditions (i.e., asthma, chronic bronchitis, severe heart disease, stroke complications, diabetes, severe back pain, osteoarthritis, rheumatism, cancer, memory impairment due to neurological disease or dementia, age-related memory impairment, and depression) was endorsed.

^2^Young Adults: 18 to 40 years, Middle-aged Adults: 41 to 64 years, Older Adults: over 65 years. 16^th^ percentile represents the cut-off for impaired HRQoL

*Note*. *N*: absolute frequencies. %: percentiles. *M*: mean. *SD*: standard deviation.

**Table A6:** Reference values for QoLIBRI-OS by Education

Sex	**Chronic Health Condition** ^**1**^	**Education** ^**2**^	N	M	SD	2.5%	5%	16%	30%	40%	50%	60%	70%	85%	95%	97.5%
Female	No	Low Education	56	60.42	16.15	33	36	46	50	54	58	67	71	79	83	86
		Medium Education	560	63.65	17.86	25	33	46	54	58	67	71	75	79	88	96
		High Education	192	68.08	16.93	25	38	50	62	67	71	75	79	83	92	92
Female	Yes	Low Education	95	55.26	21.75	14	17	33	46	50	54	62	71	75	85	97
		Medium Education	725	57.95	19.32	17	21	38	50	54	58	67	71	75	87	88
		High Education	149	61.38	17.81	24	29	42	54	58	62	67	71	79	83	93
Male	No	Low Education	60	55.49	22.59	0	16	35	50	50	54	64	71	75	88	88
		Medium Education	484	65.72	18.11	25	33	50	58	62	71	75	75	83	92	96
		High Education	242	70.52	15.6	33	42	54	62	67	75	75	79	88	92	92
Male	Yes	Low Education	81	52.83	19.67	12	17	37	46	50	54	58	62	75	79	83
		Medium Education	614	58.43	18.95	17	21	42	50	54	62	67	71	75	83	90
		High Education	239	60.91	19.23	16	25	42	54	58	67	71	71	79	88	88

^1^Codedas present, if any of listed conditions (i.e., asthma, chronic bronchitis, severe heart disease, stroke complications, diabetes, severe back pain, osteoarthritis, rheumatism, cancer, memory impairment due to neurological disease or dementia, age-related memory impairment, and depression) was endorsed.

^2^Low Education: no degree or primary school, Medium Education: secondary school, high school, vocational school or trade or technical certificate; High Education: university or college degree. 16^th^ percentile represents the cut-off for impaired HRQoL

*Note*. *N*: absolute frequencies. %: percentiles. *M*: mean. *SD*: standard deviation.

## Abbreviations

AIC: Akaike’s Information Criterion
BIC: Bayesian information criterion
CENTER-TBI: Collaborative European NeuroTrauma Effectiveness Research in Traumatic Brain Injury
CFA: Confirmatory factor analysis
CFI: Comparative fit index
CI: Confidence interval
HRQoL: Health-related quality of life
MCS: Mental component summary score
MI: Measurement invariance
MLR: Maximum likelihood robust
PROM: Patient-reported outcome measure
QoLIBRI: Quality of Life after Brain Injury
QoLIBRI-OS: Quality of Life after Brain Injury – Overall Scale
RMSEA: Root mean square error of approximation
SF-36: Short Form 36
SRMR: Root mean square residual
TBI: Traumatic brain injury
TLI: Tucker-Lewis index
WLS: Weighted Least Squares
