# Supplementary material for: Reference values for the quality of life after traumatic brain injury questionnaire and its overall scale from the adult German general population
Source: J Patient Rep Outcomes. 2026 Feb 9;10:39. doi: 10.1186/s41687-026-01014-3 (PMC12988058; doi:10.1186/s41687-026-01014-3)
Supplement: Supplementary file 1 — Supplementary Material 1 [file 41687_2026_1014_MOESM1_ESM.docx]

**Reference Values for the Quality of Life After Traumatic Brain Injury Questionnaire and Its Overall Scale from the Adult German General Population**

*Supplemental Material*

Table of Contents

Confirmatory Factor Analysis 2

QoLIBRI – WLS estimator 2

QoLIBRI – MLR Estimator 5

QoLIBRI–OS – WLS estimator 8

QoLIBRI–OS – MLR estimator 9

Propensity Score Matching 10

Measurement Invariance 12

Overview on MI Analyses Results 12

QoLIBRI – MLR 13

Model 1 13

Model 2 19

Model 3 25

Model 4 31

QoLIBRI–OS – MLR 37

Model 1 37

Model 2 38

Model 3 39

Model 4 40

Regression Analysis 41

Overview on Regression Analyses Results 41

QoLIBRI(-OS) Scales and Second–Order Regression Analyses 42

Cognition 42

Self 43

Daily Life and Autonomy 44

Social Relationships 45

Emotional Problems 46

Physical Problems 47

QoLIBRI Total Score 48

QoLIBRI-OS Total Score 49

# Confirmatory Factor Analysis

## QoLIBRI – WLS estimator

Table S1. Factor Loadings of Confirmatory Factor Analysis for QoLIBRI Scales (WLS estimator)

| Scale | Items | Estimate | *SE* | *z* | *p* | 95% CI  lower | 95% CI  upper |
| --- | --- | --- | --- | --- | --- | --- | --- |
| Cognition | A1 | 0.78 | 0.01 | 86.09 | <0.001 | 0.77 | 0.80 |
|  | A2 | 0.79 | 0.01 | 92.48 | <0.001 | 0.77 | 0.81 |
|  | A3 | 0.68 | 0.01 | 64.69 | <0.001 | 0.66 | 0.70 |
|  | A4 | 0.80 | 0.01 | 96.60 | <0.001 | 0.79 | 0.82 |
|  | A5 | 0.81 | 0.01 | 94.07 | <0.001 | 0.80 | 0.83 |
|  | A6 | 0.70 | 0.01 | 60.01 | <0.001 | 0.68 | 0.72 |
|  | A7 | 0.80 | 0.01 | 98.89 | <0.001 | 0.79 | 0.82 |
| Self | B1 | 0.73 | 0.01 | 79.16 | <0.001 | 0.71 | 0.75 |
|  | B2 | 0.78 | 0.01 | 95.72 | <0.001 | 0.76 | 0.79 |
|  | B3 | 0.86 | 0.01 | 135.62 | <0.001 | 0.85 | 0.87 |
|  | B4 | 0.67 | 0.01 | 68.84 | <0.001 | 0.65 | 0.69 |
|  | B5 | 0.80 | 0.01 | 110.02 | <0.001 | 0.79 | 0.82 |
|  | B6 | 0.85 | 0.01 | 140.49 | <0.001 | 0.84 | 0.86 |
|  | B7 | 0.75 | 0.01 | 88.38 | <0.001 | 0.73 | 0.77 |
| Daily Life and Autonomy | C1 | 0.74 | 0.01 | 81.76 | <0.001 | 0.72 | 0.76 |
|  | C2 | 0.75 | 0.01 | 82.97 | <0.001 | 0.73 | 0.77 |
|  | C3 | 0.71 | 0.01 | 70.03 | <0.001 | 0.69 | 0.73 |
|  | C4 | 0.74 | 0.01 | 75.44 | <0.001 | 0.72 | 0.76 |
|  | C5 | 0.64 | 0.01 | 55.90 | <0.001 | 0.62 | 0.66 |
|  | C6 | 0.69 | 0.01 | 69.94 | <0.001 | 0.67 | 0.71 |
|  | C7 | 0.82 | 0.01 | 113.42 | <0.001 | 0.80 | 0.83 |
| Social Relationships | D1 | 0.80 | 0.01 | 89.65 | <0.001 | 0.78 | 0.81 |
|  | D2 | 0.73 | 0.01 | 69.90 | <0.001 | 0.71 | 0.75 |
|  | D3 | 0.75 | 0.01 | 75.78 | <0.001 | 0.73 | 0.77 |
|  | D4 | 0.67 | 0.01 | 57.40 | <0.001 | 0.65 | 0.69 |
|  | D5 | 0.54 | 0.01 | 39.52 | <0.001 | 0.51 | 0.57 |
|  | D6 | 0.82 | 0.01 | 87.56 | <0.001 | 0.80 | 0.84 |
| Emotional Problems | E1 | 0.78 | 0.01 | 72.36 | <0.001 | 0.76 | 0.80 |
|  | E2 | 0.67 | 0.01 | 54.64 | <0.001 | 0.65 | 0.70 |
|  | E3 | 0.85 | 0.01 | 100.87 | <0.001 | 0.83 | 0.86 |
|  | E4 | 0.90 | 0.01 | 118.61 | <0.001 | 0.88 | 0.91 |
|  | E5 | 0.75 | 0.01 | 67.29 | <0.001 | 0.73 | 0.77 |
| Physical Problems | F1 | 0.81 | 0.01 | 75.04 | <0.001 | 0.79 | 0.83 |
|  | F2 | 0.76 | 0.01 | 68.93 | <0.001 | 0.74 | 0.78 |
|  | F3 | 0.75 | 0.01 | 67.51 | <0.001 | 0.73 | 0.77 |
|  | F4 | 0.64 | 0.01 | 48.13 | <0.001 | 0.62 | 0.67 |
|  | F5 | 0.82 | 0.01 | 80.12 | <0.001 | 0.80 | 0.84 |

*Note*. Estimate: factor loading, *SE*: standard error, z: z-value, *p*: *p*-value, 95% CI: 95% confidence interval.

Table S2. Variances of QoLIBRI Items in Confirmatory Factor Analysis (WLS estimator)

| Scale | Items | Estimate | *SE* | *z* | *p* | 95% CI  lower | 95% CI  upper |
| --- | --- | --- | --- | --- | --- | --- | --- |
| Cognition | A1 | 0.38 | 0.01 | 26.83 | <0.001 | 0.36 | 0.41 |
|  | A2 | 0.37 | 0.01 | 27.74 | <0.001 | 0.35 | 0.40 |
|  | A3 | 0.54 | 0.01 | 37.69 | <0.001 | 0.51 | 0.57 |
|  | A4 | 0.36 | 0.01 | 26.85 | <0.001 | 0.33 | 0.38 |
|  | A5 | 0.34 | 0.01 | 24.31 | <0.001 | 0.31 | 0.37 |
|  | A6 | 0.51 | 0.02 | 31.01 | <0.001 | 0.48 | 0.54 |
|  | A7 | 0.35 | 0.01 | 27.01 | <0.001 | 0.33 | 0.38 |
| Self | B1 | 0.46 | 0.01 | 34.16 | <0.001 | 0.44 | 0.49 |
|  | B2 | 0.40 | 0.01 | 31.75 | <0.001 | 0.37 | 0.42 |
|  | B3 | 0.26 | 0.01 | 24.34 | <0.001 | 0.24 | 0.29 |
|  | B4 | 0.56 | 0.01 | 43.00 | <0.001 | 0.53 | 0.58 |
|  | B5 | 0.36 | 0.01 | 30.35 | <0.001 | 0.33 | 0.38 |
|  | B6 | 0.28 | 0.01 | 27.06 | <0.001 | 0.26 | 0.30 |
|  | B7 | 0.44 | 0.01 | 34.27 | <0.001 | 0.41 | 0.46 |
| Daily Life and Autonomy | C1 | 0.45 | 0.01 | 33.30 | <0.001 | 0.42 | 0.48 |
|  | C2 | 0.44 | 0.01 | 32.62 | <0.001 | 0.41 | 0.47 |
|  | C3 | 0.50 | 0.01 | 34.37 | <0.001 | 0.47 | 0.52 |
|  | C4 | 0.46 | 0.01 | 31.56 | <0.001 | 0.43 | 0.48 |
|  | C5 | 0.59 | 0.01 | 40.56 | <0.001 | 0.56 | 0.62 |
|  | C6 | 0.53 | 0.01 | 39.29 | <0.001 | 0.50 | 0.56 |
|  | C7 | 0.33 | 0.01 | 27.93 | <0.001 | 0.31 | 0.35 |
| Social Relationships | D1 | 0.37 | 0.01 | 25.92 | <0.001 | 0.34 | 0.39 |
|  | D2 | 0.47 | 0.02 | 31.19 | <0.001 | 0.44 | 0.50 |
|  | D3 | 0.43 | 0.01 | 29.09 | <0.001 | 0.41 | 0.46 |
|  | D4 | 0.55 | 0.02 | 35.54 | <0.001 | 0.52 | 0.58 |
|  | D5 | 0.71 | 0.01 | 48.21 | <0.001 | 0.68 | 0.74 |
|  | D6 | 0.33 | 0.02 | 21.59 | <0.001 | 0.30 | 0.36 |
| Emotional Problems | E1 | 0.39 | 0.02 | 23.37 | <0.001 | 0.36 | 0.43 |
|  | E2 | 0.54 | 0.02 | 32.65 | <0.001 | 0.51 | 0.58 |
|  | E3 | 0.28 | 0.01 | 19.98 | <0.001 | 0.26 | 0.31 |
|  | E4 | 0.20 | 0.01 | 14.50 | <0.001 | 0.17 | 0.22 |
|  | E5 | 0.43 | 0.02 | 25.87 | <0.001 | 0.40 | 0.47 |
| Physical Problems | F1 | 0.34 | 0.02 | 19.23 | <0.001 | 0.30 | 0.37 |
|  | F2 | 0.42 | 0.02 | 24.73 | <0.001 | 0.38 | 0.45 |
|  | F3 | 0.44 | 0.02 | 26.70 | <0.001 | 0.41 | 0.47 |
|  | F4 | 0.58 | 0.02 | 33.80 | <0.001 | 0.55 | 0.62 |
|  | F5 | 0.33 | 0.02 | 19.70 | <0.001 | 0.30 | 0.36 |

*Note*. Estimate: variance, *SE*: standard error, z: z-value, *p*: *p*-value, 95% CI: 95% confidence interval.

Table S3. Covariances between QoLIBRI Scales in Confirmatory Factor Analysis (WLS estimator)

| Scale | Scale | Estimate | *SE* | *z* | *p* | 95% CI lower | 95% CI upper |
| --- | --- | --- | --- | --- | --- | --- | --- |
| Cognition | Self | 0.69 | 0.01 | 71.22 | <0.001 | 0.68 | 0.71 |
|  | Daily Life and Autonomy | 0.74 | 0.01 | 74.50 | <0.001 | 0.72 | 0.76 |
|  | Social Relationships | 0.59 | 0.01 | 46.09 | <0.001 | 0.57 | 0.62 |
|  | Emotional Problems | 0.52 | 0.01 | 36.82 | <0.001 | 0.49 | 0.55 |
|  | Physical Problems | 0.41 | 0.02 | 25.32 | <0.001 | 0.38 | 0.44 |
| Self | Daily Life and Autonomy | 0.82 | 0.01 | 111.41 | <0.001 | 0.81 | 0.84 |
|  | Social Relationships | 0.72 | 0.01 | 75.01 | <0.001 | 0.70 | 0.74 |
|  | Emotional Problems | 0.47 | 0.01 | 32.88 | <0.001 | 0.44 | 0.50 |
|  | Physical Problems | 0.32 | 0.02 | 19.46 | <0.001 | 0.29 | 0.36 |
| Daily Life  and  Autonomy | Social Relationships | 0.75 | 0.01 | 77.94 | <0.001 | 0.73 | 0.77 |
|  | Emotional Problems | 0.51 | 0.01 | 34.85 | <0.001 | 0.48 | 0.54 |
|  | Physical Problems | 0.41 | 0.02 | 24.39 | <0.001 | 0.37 | 0.44 |
| Social Relationships | Emotional Problems | 0.43 | 0.02 | 26.84 | <0.001 | 0.39 | 0.46 |
|  | Physical Problems | 0.25 | 0.02 | 13.56 | <0.001 | 0.21 | 0.28 |
| Emotional Problems | Physical Problems | 0.69 | 0.01 | 60.31 | <0.001 | 0.67 | 0.72 |

*Note*. Estimate: covariance, *SE*: standard error, z: z-value, *p*: *p*-value, 95% CI: 95% confidence interval.

## QoLIBRI – MLR Estimator

Table S4. Factor Loadings of Confirmatory Factor Analysis for QoLIBRI Scales (MLR estimator)

| Scale | Items | Estimator | *SE* | *z* | *p* | 95% CI  lower | 95% CI  upper |
| --- | --- | --- | --- | --- | --- | --- | --- |
| Cognition | A1 | 0.72 | 0.01 | 60.41 | <0.001 | 0.70 | 0.75 |
|  | A2 | 0.75 | 0.01 | 76.30 | <0.001 | 0.73 | 0.77 |
|  | A3 | 0.66 | 0.01 | 54.76 | <0.001 | 0.64 | 0.69 |
|  | A4 | 0.76 | 0.01 | 75.28 | <0.001 | 0.74 | 0.78 |
|  | A5 | 0.73 | 0.01 | 66.15 | <0.001 | 0.70 | 0.75 |
|  | A6 | 0.64 | 0.01 | 48.42 | <0.001 | 0.61 | 0.66 |
|  | A7 | 0.77 | 0.01 | 78.64 | <0.001 | 0.75 | 0.79 |
| Self | B1 | 0.66 | 0.01 | 51.16 | <0.001 | 0.63 | 0.68 |
|  | B2 | 0.72 | 0.01 | 65.96 | <0.001 | 0.69 | 0.74 |
|  | B3 | 0.81 | 0.01 | 101.18 | <0.001 | 0.79 | 0.82 |
|  | B4 | 0.67 | 0.01 | 55.46 | <0.001 | 0.64 | 0.69 |
|  | B5 | 0.77 | 0.01 | 81.99 | <0.001 | 0.75 | 0.79 |
|  | B6 | 0.83 | 0.01 | 106.28 | <0.001 | 0.81 | 0.84 |
|  | B7 | 0.72 | 0.01 | 65.81 | <0.001 | 0.70 | 0.74 |
| Daily Life and Autonomy | C1 | 0.70 | 0.01 | 63.06 | <0.001 | 0.68 | 0.72 |
|  | C2 | 0.72 | 0.01 | 66.62 | <0.001 | 0.70 | 0.74 |
|  | C3 | 0.66 | 0.01 | 53.97 | <0.001 | 0.64 | 0.68 |
|  | C4 | 0.64 | 0.01 | 48.18 | <0.001 | 0.61 | 0.66 |
|  | C5 | 0.58 | 0.01 | 39.94 | <0.001 | 0.56 | 0.61 |
|  | C6 | 0.66 | 0.01 | 54.18 | <0.001 | 0.64 | 0.69 |
|  | C7 | 0.79 | 0.01 | 90.80 | <0.001 | 0.77 | 0.80 |
| Social  Relationships | D1 | 0.74 | 0.01 | 65.55 | <0.001 | 0.72 | 0.76 |
|  | D2 | 0.69 | 0.01 | 54.57 | <0.001 | 0.67 | 0.72 |
|  | D3 | 0.71 | 0.01 | 61.00 | <0.001 | 0.69 | 0.73 |
|  | D4 | 0.63 | 0.01 | 44.89 | <0.001 | 0.61 | 0.66 |
|  | D5 | 0.53 | 0.02 | 34.49 | <0.001 | 0.50 | 0.56 |
|  | D6 | 0.71 | 0.01 | 59.57 | <0.001 | 0.68 | 0.73 |
| Emotional  Problems | E1 | 0.72 | 0.01 | 62.18 | <0.001 | 0.70 | 0.74 |
|  | E2 | 0.64 | 0.01 | 47.22 | <0.001 | 0.62 | 0.67 |
|  | E3 | 0.81 | 0.01 | 97.80 | <0.001 | 0.79 | 0.83 |
|  | E4 | 0.85 | 0.01 | 107.96 | <0.001 | 0.83 | 0.86 |
|  | E5 | 0.71 | 0.01 | 62.80 | <0.001 | 0.69 | 0.73 |
| Physical  Problems | F1 | 0.75 | 0.01 | 67.10 | <0.001 | 0.72 | 0.77 |
|  | F2 | 0.73 | 0.01 | 63.77 | <0.001 | 0.71 | 0.75 |
|  | F3 | 0.72 | 0.01 | 63.81 | <0.001 | 0.70 | 0.75 |
|  | F4 | 0.62 | 0.01 | 46.78 | <0.001 | 0.60 | 0.65 |
|  | F5 | 0.75 | 0.01 | 68.54 | <0.001 | 0.73 | 0.77 |

*Note*. Estimate: factor loading, *SE*: standard error, z: z-value, *p*: *p*-value, 95% CI: 95% confidence interval.

Table S5. Variances of QoLIBRI Items in Confirmatory Factor Analysis (MLR estimator)

| Scale | Items | Estimate | *SE* | *z* | *p* | 95%  CI lower | 95%  CI upper |
| --- | --- | --- | --- | --- | --- | --- | --- |
| Cognition | A1 | 0.48 | 0.02 | 27.34 | <0.001 | 0.44 | 0.51 |
|  | A2 | 0.44 | 0.01 | 29.51 | <0.001 | 0.41 | 0.47 |
|  | A3 | 0.56 | 0.02 | 34.98 | <0.001 | 0.53 | 0.59 |
|  | A4 | 0.42 | 0.02 | 27.60 | <0.001 | 0.39 | 0.45 |
|  | A5 | 0.47 | 0.02 | 29.72 | <0.001 | 0.44 | 0.50 |
|  | A6 | 0.59 | 0.02 | 35.16 | <0.001 | 0.56 | 0.63 |
|  | A7 | 0.41 | 0.01 | 27.59 | <0.001 | 0.38 | 0.44 |
| Self | B1 | 0.57 | 0.02 | 33.31 | <0.001 | 0.53 | 0.60 |
|  | B2 | 0.49 | 0.02 | 31.31 | <0.001 | 0.46 | 0.52 |
|  | B3 | 0.35 | 0.01 | 27.00 | <0.001 | 0.32 | 0.37 |
|  | B4 | 0.56 | 0.02 | 34.68 | <0.001 | 0.52 | 0.59 |
|  | B5 | 0.41 | 0.01 | 28.43 | <0.001 | 0.38 | 0.44 |
|  | B6 | 0.32 | 0.01 | 24.74 | <0.001 | 0.29 | 0.34 |
|  | B7 | 0.48 | 0.02 | 30.91 | <0.001 | 0.45 | 0.52 |
| Daily Life and Autonomy | C1 | 0.51 | 0.02 | 32.37 | <0.001 | 0.48 | 0.54 |
|  | C2 | 0.48 | 0.02 | 30.82 | <0.001 | 0.45 | 0.51 |
|  | C3 | 0.57 | 0.02 | 35.06 | <0.001 | 0.53 | 0.60 |
|  | C4 | 0.59 | 0.02 | 35.27 | <0.001 | 0.56 | 0.63 |
|  | C5 | 0.66 | 0.02 | 38.44 | <0.001 | 0.62 | 0.69 |
|  | C6 | 0.56 | 0.02 | 34.75 | <0.001 | 0.53 | 0.59 |
|  | C7 | 0.38 | 0.01 | 27.79 | <0.001 | 0.35 | 0.41 |
| Social  Relationships | D1 | 0.45 | 0.02 | 27.22 | <0.001 | 0.42 | 0.49 |
|  | D2 | 0.52 | 0.02 | 29.74 | <0.001 | 0.49 | 0.56 |
|  | D3 | 0.50 | 0.02 | 30.35 | <0.001 | 0.47 | 0.53 |
|  | D4 | 0.60 | 0.02 | 33.41 | <0.001 | 0.56 | 0.63 |
|  | D5 | 0.72 | 0.02 | 44.20 | <0.001 | 0.69 | 0.75 |
|  | D6 | 0.50 | 0.02 | 29.82 | <0.001 | 0.47 | 0.53 |
| Emotional  Problems | E1 | 0.48 | 0.02 | 29.11 | <0.001 | 0.45 | 0.52 |
|  | E2 | 0.59 | 0.02 | 33.48 | <0.001 | 0.55 | 0.62 |
|  | E3 | 0.35 | 0.01 | 25.85 | <0.001 | 0.32 | 0.37 |
|  | E4 | 0.28 | 0.01 | 21.33 | <0.001 | 0.26 | 0.31 |
|  | E5 | 0.50 | 0.02 | 30.83 | <0.001 | 0.46 | 0.53 |
| Physical  Problems | F1 | 0.44 | 0.02 | 26.88 | <0.001 | 0.41 | 0.48 |
|  | F2 | 0.47 | 0.02 | 28.21 | <0.001 | 0.44 | 0.50 |
|  | F3 | 0.48 | 0.02 | 29.02 | <0.001 | 0.44 | 0.51 |
|  | F4 | 0.61 | 0.02 | 36.52 | <0.001 | 0.58 | 0.64 |
|  | F5 | 0.43 | 0.02 | 26.14 | <0.001 | 0.40 | 0.47 |

*Note*. Estimate: variance, *SE*: standard error, z: z-value, *p*: *p*-value, 95% CI: 95% confidence interval.

Table S6. Covariances between QoLIBRI Scales in Confirmatory Factor Analysis (MLR estimator)

| Scale | Scale | Estimate | *SE* | *z* | *p* | 95%  CI lower | 95%  CI upper |
| --- | --- | --- | --- | --- | --- | --- | --- |
| Cognition | Self | 0.68 | 0.01 | 51.61 | <0.001 | 0.66 | 0.71 |
|  | Daily Life and Autonomy | 0.71 | 0.01 | 50.17 | <0.001 | 0.68 | 0.74 |
|  | Social Relationships | 0.59 | 0.02 | 35.01 | <0.001 | 0.56 | 0.62 |
|  | Emotional Problems | 0.50 | 0.02 | 30.50 | <0.001 | 0.47 | 0.54 |
|  | Physical Problems | 0.40 | 0.02 | 21.91 | <0.001 | 0.36 | 0.43 |
| Self | Daily Life and Autonomy | 0.82 | 0.01 | 78.84 | <0.001 | 0.80 | 0.84 |
|  | Social Relationships | 0.73 | 0.01 | 55.35 | <0.001 | 0.70 | 0.75 |
|  | Emotional Problems | 0.48 | 0.02 | 25.25 | <0.001 | 0.45 | 0.52 |
|  | Physical Problems | 0.32 | 0.02 | 15.16 | <0.001 | 0.28 | 0.36 |
| Daily Life  and  Autonomy | Social Relationships | 0.76 | 0.01 | 56.28 | <0.001 | 0.73 | 0.78 |
|  | Emotional Problems | 0.50 | 0.02 | 27.06 | <0.001 | 0.46 | 0.54 |
|  | Physical Problems | 0.40 | 0.02 | 20.35 | <0.001 | 0.36 | 0.44 |
| Social Relationships | Emotional Problems | 0.42 | 0.02 | 21.26 | <0.001 | 0.38 | 0.46 |
|  | Physical Problems | 0.25 | 0.02 | 11.91 | <0.001 | 0.21 | 0.29 |
| Emotional Problems | Physical Problems | 0.68 | 0.01 | 47.77 | <0.001 | 0.66 | 0.71 |

*Note*. Estimate: covariance, *SE*: standard error, z: z-value, *p*: *p*-value, 95% CI: 95% confidence interval.

## QoLIBRI–OS – WLS estimator

Table S7. Factor Loadings of Confirmatory Factor Analysis for QoLIBRI-OS (WLS estimator)

| Scale | Items | Estimate | *SE* | *z* | *p* | 95% CI  lower | 95% CI  upper |
| --- | --- | --- | --- | --- | --- | --- | --- |
| QoLIBRI-OS | O1 | 0.66 | 0.01 | 61.98 | <0.001 | 0.64 | 0.68 |
|  | O2 | 0.70 | 0.01 | 72.23 | <0.001 | 0.68 | 0.72 |
|  | O3 | 0.78 | 0.01 | 103.24 | <0.001 | 0.77 | 0.80 |
|  | O4 | 0.79 | 0.01 | 102.76 | <0.001 | 0.78 | 0.81 |
|  | O5 | 0.79 | 0.01 | 103.58 | <0.001 | 0.77 | 0.80 |
|  | O6 | 0.78 | 0.01 | 101.87 | <0.001 | 0.77 | 0.80 |

*Note*. Estimate: factor loading, *SE*: standard error, z: z-value, *p*: *p*-value, 95% CI: 95% confidence interval.

Table S8. Variances of QoLIBRI-OS Items in Confirmatory Factor Analysis (WLS estimator)

| Scale | Items | Estimate | *SE* | *z* | *p* | 95% CI  lower | 95% CI  upper |
| --- | --- | --- | --- | --- | --- | --- | --- |
| QoLIBRI-OS | O1 | 0.57 | 0.01 | 40.96 | <0.001 | 0.54 | 0.60 |
|  | O2 | 0.51 | 0.01 | 38.08 | <0.001 | 0.49 | 0.54 |
|  | O3 | 0.38 | 0.01 | 32.32 | <0.001 | 0.36 | 0.41 |
|  | O4 | 0.37 | 0.01 | 30.10 | <0.001 | 0.35 | 0.39 |
|  | O5 | 0.38 | 0.01 | 32.05 | <0.001 | 0.36 | 0.41 |
|  | O6 | 0.39 | 0.01 | 32.27 | <0.001 | 0.36 | 0.41 |

*Note*. Estimate: variance, *SE*: standard error, z: z-value, *p*: *p*-value, 95% CI: 95% confidence interval.

## QoLIBRI–OS – MLR estimator

Table S9. Factor Loadings of Confirmatory Factor Analysis for QOLIBRI OS (MLR estimator)

| Scale | Items | Estimate | *SE* | *z* | *p* | 95% CI  lower | 95% CI  upper |
| --- | --- | --- | --- | --- | --- | --- | --- |
| QoLIBRI-OS | O1 | 0.63 | 0.01 | 46.34 | <0.001 | 0.60 | 0.65 |
|  | O2 | 0.65 | 0.01 | 44.25 | <0.001 | 0.62 | 0.68 |
|  | O3 | 0.75 | 0.01 | 67.76 | <0.001 | 0.73 | 0.78 |
|  | O4 | 0.75 | 0.01 | 64.26 | <0.001 | 0.73 | 0.77 |
|  | O5 | 0.74 | 0.01 | 64.43 | <0.001 | 0.72 | 0.77 |
|  | O6 | 0.75 | 0.01 | 67.80 | <0.001 | 0.73 | 0.77 |

*Note*. Estimate: factor loading, *SE*: standard error, z: z-value, *p*: *p*-value, 95% CI: 95% confidence interval.

Table S10. Variances of QOLIBRI OS Items in Confirmatory Factor Analysis (MLR estimator)

| Scale | Items | Estimate | *SE* | *z* | *p* | 95% CI  lower | 95% CI  upper |
| --- | --- | --- | --- | --- | --- | --- | --- |
| QoLIBRI-OS | O1 | 0.61 | 0.02 | 36.03 | <0.001 | 0.58 | 0.64 |
|  | O2 | 0.58 | 0.02 | 30.66 | <0.001 | 0.54 | 0.62 |
|  | O3 | 0.43 | 0.02 | 25.64 | <0.001 | 0.40 | 0.46 |
|  | O4 | 0.44 | 0.02 | 24.94 | <0.001 | 0.40 | 0.47 |
|  | O5 | 0.45 | 0.02 | 26.10 | <0.001 | 0.41 | 0.48 |
|  | O6 | 0.44 | 0.02 | 26.14 | <0.001 | 0.40 | 0.47 |

*Note*. Estimate: variance, *SE*: standard error, z: z-value, *p*: *p*-value, 95% CI: 95% confidence interval.

# Propensity Score Matching

Figure S1. Love plot on balance in the matched sample (absolute standardized mean difference) . Matching based on age, gender and education. Distance: estimate of propensity scores. Filled circles show the difference after matching; empty circles show the difference before matching.

Table S11. Sample Characteristics of the Matched Samples.

|  |  | General  Population Sample | TBI  Sample | Total |
| --- | --- | --- | --- | --- |
| Characteristics | Group/Value | *N* = 353 | *N* = 353 | *N* = 706 |
| Sex | Female | 109 (30.9%) | 109 (30.9%) | 218 (30.9%) |
|  | Male | 244 (69.1%) | 244 (69.1%) | 488 (69.1%) |
| Age in years | *M* (*SD*) | 47.4 (16.4) | 47.3 (16.7) | 47.3 (16.5) |
|  | *Mdn* [Min, Max] | 49.0 [18, 82] | 49.0 [17, 86] | 49.0 [17, 86] |
| Education | Low Education | 18 (5.1%) | 19 (5.4%) | 37 (5.2%) |
|  | Medium Education | 202 (57.2%) | 201 (56.9%) | 403 (57.1%) |
|  | High Education | 133 (37.7%) | 133 (37.7%) | 266 (37.7%) |

Note. *N*: absolute frequencies. %: relative frequencies. *M*: mean. *SD*: standard deviation. Mdn: median. Min: minimum. Max: maximum. Low Education: no degree or primary school, Medium Education: secondary school, high school, vocational school or trade or technical certificate; High Education: university or college degree

# Measurement Invariance

## Overview on MI Analyses Results

Table S12. Summary on Measurement Invariance Analyses Results (MLR Estimator)

|  |  |  |  |  |  |  |  |  | Difference test | | | | | |
| --- | --- | --- | --- | --- | --- | --- | --- | --- | --- | --- | --- | --- | --- | --- |
| Scale | Model | χ2 | *df* | AIC | BIC | RMSEA | CFI | TLI | ΔRMSEA | ΔCFI | ΔTLI | Δχ2 | Δ*df* | *p* |
| QoLIBRI | Model 1 (Configural Variance) | 970.73 | 1228 | 46194 | 47246 | 0.066 | 0.873 | 0.862 | – | – | – | – | – | – |
|  | Model 2 (Metric Invariance) | 1227.27 | 1259 | 46202 | 47125 | 0.066 | 0.869 | 0.861 | <0.01 | -0.004 | -0.001 | 60.55 | 31 | 0.001 |
|  | Model 3 (Scalar Invariance) | 1263.37 | 1290 | 46223 | 47016 | 0.067 | 0.864 | 0.859 | <0.01 | -0.005 | -0.002 | 71.55 | 31 | <0.001 |
|  | Model 4 (Strict Invariance) | 1311.26 | 1327 | 46233 | 46873 | 0.067 | 0.861 | 0.86 | <0.01 | -0.003 | 0.001 | 65.74 | 37 | 0.002 |
| QoLIBRI-OS | Model 1 (Configural Variance) | 9.43 | 18 | 9469 | 9629 | 0.088 | 0.976 | 0.959 | – | – | – | – | – | – |
|  | Model 2 (Metric Invariance) | 21.33 | 23 | 9472 | 9610 | 0.084 | 0.971 | 0.962 | -0.003 | -0.004 | 0.003 | 12.08 | 5 | 0.034 |
|  | Model 3 (Scalar Invariance) | 45.4 | 28 | 9510 | 9625 | 0.103 | 0.948 | 0.944 | 0.018 | -0.023 | -0.018 | 53.6 | 5 | <0.001 |
|  | Model 4 (Strict Invariance) | 56.41 | 34 | 9513 | 9602 | 0.097 | 0.944 | 0.95 | -0.006 | -0.004 | 0.006 | 18.31 | 6 | 0.005 |

*Note*. Scaled values are reported for χ^2^, robust CFI, TLI, and RMSEA. *df*: degrees of freedom, *p*: *p*-value, CFI: comparative fit index, TLI: Tucker-Lewis index, RMSEA: root mean square error of approximation, AIC: Akaike’s information criterion, BIC: Bayesian information criterion, Δ sign prior to measure indicates fit measure difference calculated as a raw difference between the model with higher constraints to the model with less constraints. The Δ sign prior to the measure indicates the difference in fit between the two models, which is calculated as the raw difference between the model with the more restrictive constraints and the model with the fewer constraints.

## QoLIBRI – MLR

### Model 1

Table S13. Standardized Factor Loadings for Measurement Invariance Model 1 (Configural Invariance) for QoLIBRI Scales (MLR Estimator)

|  |  | Group 1 | | | | | | Group 2 | | | | | |
| --- | --- | --- | --- | --- | --- | --- | --- | --- | --- | --- | --- | --- | --- |
| Scale | Items | Estimate | *SE* | *z* | *p* | 95% CI lower | 95% CI upper | Estimate | *SE* | *z* | *p* | 95% CI lower | 95% CI upper |
| Cognition | A1 | 0.72 | 0.05 | 14.42 | <0.001 | 0.62 | 0.81 | 0.88 | 0.03 | 34.10 | <0.001 | 0.83 | 0.93 |
|  | A2 | 0.69 | 0.04 | 17.15 | <0.001 | 0.61 | 0.77 | 0.83 | 0.05 | 17.22 | <0.001 | 0.73 | 0.92 |
|  | A3 | 0.69 | 0.04 | 18.54 | <0.001 | 0.62 | 0.77 | 0.77 | 0.05 | 15.61 | <0.001 | 0.67 | 0.86 |
|  | A4 | 0.79 | 0.03 | 27.46 | <0.001 | 0.73 | 0.85 | 0.81 | 0.04 | 22.58 | <0.001 | 0.74 | 0.88 |
|  | A5 | 0.66 | 0.04 | 15.07 | <0.001 | 0.57 | 0.74 | 0.76 | 0.05 | 14.65 | <0.001 | 0.66 | 0.87 |
|  | A6 | 0.60 | 0.05 | 12.22 | <0.001 | 0.51 | 0.70 | 0.68 | 0.07 | 10.16 | <0.001 | 0.55 | 0.81 |
|  | A7 | 0.75 | 0.04 | 21.38 | <0.001 | 0.68 | 0.82 | 0.88 | 0.03 | 31.49 | <0.001 | 0.83 | 0.94 |
| Self | B1 | 0.65 | 0.04 | 15.66 | <0.001 | 0.57 | 0.73 | 0.82 | 0.03 | 26.19 | <0.001 | 0.76 | 0.89 |
|  | B2 | 0.69 | 0.04 | 18.06 | <0.001 | 0.62 | 0.77 | 0.78 | 0.04 | 20.10 | <0.001 | 0.70 | 0.86 |
|  | B3 | 0.79 | 0.02 | 32.47 | <0.001 | 0.74 | 0.84 | 0.85 | 0.03 | 29.18 | <0.001 | 0.79 | 0.90 |
|  | B4 | 0.63 | 0.04 | 14.87 | <0.001 | 0.55 | 0.71 | 0.68 | 0.05 | 12.96 | <0.001 | 0.58 | 0.79 |
|  | B5 | 0.79 | 0.03 | 29.11 | <0.001 | 0.74 | 0.84 | 0.76 | 0.04 | 17.53 | <0.001 | 0.68 | 0.85 |
|  | B6 | 0.87 | 0.02 | 42.00 | <0.001 | 0.83 | 0.91 | 0.83 | 0.03 | 25.81 | <0.001 | 0.77 | 0.89 |
|  | B7 | 0.72 | 0.03 | 23.44 | <0.001 | 0.66 | 0.78 | 0.86 | 0.03 | 34.45 | <0.001 | 0.81 | 0.91 |
| Daily Life  and  Autonomy | C1 | 0.68 | 0.04 | 19.40 | <0.001 | 0.61 | 0.75 | 0.84 | 0.05 | 17.15 | <0.001 | 0.74 | 0.93 |
|  | C2 | 0.70 | 0.04 | 17.20 | <0.001 | 0.62 | 0.78 | 0.82 | 0.05 | 18.15 | <0.001 | 0.73 | 0.91 |
|  | C3 | 0.64 | 0.05 | 14.08 | <0.001 | 0.55 | 0.72 | 0.73 | 0.04 | 17.94 | <0.001 | 0.65 | 0.81 |
|  | C4 | 0.58 | 0.05 | 11.53 | <0.001 | 0.48 | 0.68 | 0.67 | 0.06 | 10.87 | <0.001 | 0.55 | 0.79 |
|  | C5 | 0.64 | 0.04 | 15.26 | <0.001 | 0.56 | 0.72 | 0.72 | 0.05 | 13.43 | <0.001 | 0.61 | 0.82 |
|  | C6 | 0.64 | 0.04 | 14.87 | <0.001 | 0.55 | 0.72 | 0.80 | 0.04 | 21.04 | <0.001 | 0.73 | 0.88 |
|  | C7 | 0.78 | 0.03 | 29.13 | <0.001 | 0.72 | 0.83 | 0.89 | 0.02 | 38.86 | <0.001 | 0.84 | 0.93 |
| Social  Relationships | D1 | 0.69 | 0.04 | 17.74 | <0.001 | 0.62 | 0.77 | 0.81 | 0.05 | 18.01 | <0.001 | 0.72 | 0.90 |
|  | D2 | 0.66 | 0.04 | 15.13 | <0.001 | 0.57 | 0.74 | 0.75 | 0.06 | 13.06 | <0.001 | 0.64 | 0.87 |
|  | D3 | 0.71 | 0.04 | 17.90 | <0.001 | 0.63 | 0.79 | 0.81 | 0.04 | 18.41 | <0.001 | 0.72 | 0.89 |
|  | D4 | 0.61 | 0.04 | 14.53 | <0.001 | 0.53 | 0.70 | 0.67 | 0.06 | 11.79 | <0.001 | 0.56 | 0.79 |
|  | D5 | 0.61 | 0.04 | 14.36 | <0.001 | 0.53 | 0.69 | 0.68 | 0.06 | 11.27 | <0.001 | 0.56 | 0.80 |
|  | D6 | 0.72 | 0.04 | 18.49 | <0.001 | 0.64 | 0.79 | 0.72 | 0.06 | 12.39 | <0.001 | 0.60 | 0.83 |
| Emotional  Problems | E1 | 0.65 | 0.04 | 16.15 | <0.001 | 0.57 | 0.72 | 0.86 | 0.04 | 23.11 | <0.001 | 0.79 | 0.93 |
|  | E2 | 0.59 | 0.05 | 12.40 | <0.001 | 0.50 | 0.69 | 0.80 | 0.04 | 19.08 | <0.001 | 0.72 | 0.89 |
|  | E3 | 0.80 | 0.03 | 26.94 | <0.001 | 0.75 | 0.86 | 0.83 | 0.04 | 22.79 | <0.001 | 0.76 | 0.90 |
|  | E4 | 0.87 | 0.02 | 39.22 | <0.001 | 0.83 | 0.91 | 0.81 | 0.04 | 19.69 | <0.001 | 0.73 | 0.89 |
|  | E5 | 0.73 | 0.04 | 19.97 | <0.001 | 0.66 | 0.80 | 0.79 | 0.04 | 21.58 | <0.001 | 0.72 | 0.86 |
| Physical  Problems | F1 | 0.60 | 0.05 | 11.04 | <0.001 | 0.50 | 0.71 | 0.85 | 0.04 | 22.39 | <0.001 | 0.77 | 0.92 |
|  | F2 | 0.67 | 0.05 | 14.32 | <0.001 | 0.58 | 0.76 | 0.82 | 0.04 | 22.25 | <0.001 | 0.75 | 0.89 |
|  | F3 | 0.77 | 0.03 | 23.69 | <0.001 | 0.71 | 0.83 | 0.61 | 0.06 | 9.56 | <0.001 | 0.49 | 0.74 |
|  | F4 | 0.56 | 0.05 | 12.17 | <0.001 | 0.47 | 0.65 | 0.68 | 0.06 | 11.38 | <0.001 | 0.56 | 0.79 |
|  | F5 | 0.73 | 0.04 | 18.71 | <0.001 | 0.65 | 0.81 | 0.82 | 0.04 | 19.75 | <0.001 | 0.74 | 0.90 |

*Note*. Group: (1) general population sample, (2) TBI sample, Estimate: factor loading, *SE*: standard error, *z*: *z*-value, *p*: *p*-value, 95% CI: 95% confidence interval.

Table S14. Standardized Factor Variances for Measurement Invariance Model 1 (Configural Invariance) for QoLIBRI Scales (MLR Estimator)

|  |  |  | Group 1 | | | | | | Group 2 | | | | | |
| --- | --- | --- | --- | --- | --- | --- | --- | --- | --- | --- | --- | --- | --- | --- |
| Scale |  | Items | Estimate | *SE* | *z* | *p* | 95% CI lower | 95% CI upper | Estimate | *SE* | *z* | *p* | 95% CI lower | 95% CI upper |
| Cognition |  | A1 | 0.49 | 0.07 | 6.80 | <0.001 | 0.35 | 0.63 | 0.23 | 0.05 | 5.06 | <0.001 | 0.14 | 0.32 |
|  |  | A2 | 0.52 | 0.06 | 9.47 | <0.001 | 0.42 | 0.63 | 0.32 | 0.08 | 4.01 | <0.001 | 0.16 | 0.47 |
|  |  | A3 | 0.52 | 0.05 | 9.93 | <0.001 | 0.42 | 0.62 | 0.41 | 0.08 | 5.42 | <0.001 | 0.26 | 0.56 |
|  |  | A4 | 0.38 | 0.05 | 8.30 | <0.001 | 0.29 | 0.47 | 0.34 | 0.06 | 5.93 | <0.001 | 0.23 | 0.46 |
|  |  | A5 | 0.57 | 0.06 | 9.99 | <0.001 | 0.46 | 0.68 | 0.42 | 0.08 | 5.22 | <0.001 | 0.26 | 0.57 |
|  |  | A6 | 0.64 | 0.06 | 10.71 | <0.001 | 0.52 | 0.75 | 0.54 | 0.09 | 5.85 | <0.001 | 0.36 | 0.71 |
|  |  | A7 | 0.43 | 0.05 | 8.11 | <0.001 | 0.33 | 0.54 | 0.22 | 0.05 | 4.53 | <0.001 | 0.13 | 0.32 |
| Self |  | B1 | 0.57 | 0.05 | 10.53 | <0.001 | 0.47 | 0.68 | 0.32 | 0.05 | 6.16 | <0.001 | 0.22 | 0.42 |
|  |  | B2 | 0.52 | 0.05 | 9.70 | <0.001 | 0.41 | 0.62 | 0.39 | 0.06 | 6.47 | <0.001 | 0.27 | 0.51 |
|  |  | B3 | 0.38 | 0.04 | 9.87 | <0.001 | 0.30 | 0.45 | 0.28 | 0.05 | 5.72 | <0.001 | 0.19 | 0.38 |
|  |  | B4 | 0.61 | 0.05 | 11.41 | <0.001 | 0.50 | 0.71 | 0.53 | 0.07 | 7.38 | <0.001 | 0.39 | 0.67 |
|  |  | B5 | 0.38 | 0.04 | 8.75 | <0.001 | 0.29 | 0.46 | 0.42 | 0.07 | 6.24 | <0.001 | 0.29 | 0.55 |
|  |  | B6 | 0.25 | 0.04 | 6.91 | <0.001 | 0.18 | 0.32 | 0.31 | 0.05 | 5.90 | <0.001 | 0.21 | 0.42 |
|  |  | B7 | 0.49 | 0.04 | 11.13 | <0.001 | 0.40 | 0.57 | 0.26 | 0.04 | 5.96 | <0.001 | 0.17 | 0.34 |
| Daily Life  and  Autonomy |  | C1 | 0.54 | 0.05 | 11.23 | <0.001 | 0.44 | 0.63 | 0.30 | 0.08 | 3.70 | <0.001 | 0.14 | 0.46 |
|  |  | C2 | 0.51 | 0.06 | 9.13 | <0.001 | 0.40 | 0.63 | 0.33 | 0.07 | 4.48 | <0.001 | 0.19 | 0.48 |
|  |  | C3 | 0.60 | 0.06 | 10.34 | <0.001 | 0.48 | 0.71 | 0.46 | 0.06 | 7.76 | <0.001 | 0.35 | 0.58 |
|  |  | C4 | 0.66 | 0.06 | 11.25 | <0.001 | 0.55 | 0.78 | 0.55 | 0.08 | 6.55 | <0.001 | 0.38 | 0.71 |
|  |  | C5 | 0.59 | 0.05 | 11.15 | <0.001 | 0.49 | 0.70 | 0.48 | 0.08 | 6.31 | <0.001 | 0.33 | 0.63 |
|  |  | C6 | 0.60 | 0.05 | 10.98 | <0.001 | 0.49 | 0.70 | 0.36 | 0.06 | 5.81 | <0.001 | 0.24 | 0.48 |
|  |  | C7 | 0.40 | 0.04 | 9.60 | <0.001 | 0.32 | 0.48 | 0.21 | 0.04 | 5.27 | <0.001 | 0.13 | 0.29 |
| Social Relationships |  | D1 | 0.52 | 0.05 | 9.64 | <0.001 | 0.41 | 0.63 | 0.34 | 0.07 | 4.67 | <0.001 | 0.20 | 0.48 |
|  |  | D2 | 0.57 | 0.06 | 9.96 | <0.001 | 0.46 | 0.68 | 0.43 | 0.09 | 5.00 | <0.001 | 0.26 | 0.60 |
|  |  | D3 | 0.50 | 0.06 | 8.83 | <0.001 | 0.39 | 0.61 | 0.35 | 0.07 | 4.94 | <0.001 | 0.21 | 0.49 |
|  |  | D4 | 0.62 | 0.05 | 11.96 | <0.001 | 0.52 | 0.72 | 0.55 | 0.08 | 7.11 | <0.001 | 0.40 | 0.70 |
|  |  | D5 | 0.63 | 0.05 | 12.09 | <0.001 | 0.53 | 0.73 | 0.54 | 0.08 | 6.50 | <0.001 | 0.37 | 0.70 |
|  |  | D6 | 0.49 | 0.06 | 8.83 | <0.001 | 0.38 | 0.60 | 0.49 | 0.08 | 5.91 | <0.001 | 0.33 | 0.65 |
| Emotional Problems |  | E1 | 0.58 | 0.05 | 11.25 | <0.001 | 0.48 | 0.68 | 0.26 | 0.06 | 4.02 | <0.001 | 0.13 | 0.38 |
|  |  | E2 | 0.65 | 0.06 | 11.37 | <0.001 | 0.54 | 0.76 | 0.35 | 0.07 | 5.20 | <0.001 | 0.22 | 0.49 |
|  |  | E3 | 0.35 | 0.05 | 7.33 | <0.001 | 0.26 | 0.45 | 0.31 | 0.06 | 5.07 | <0.001 | 0.19 | 0.43 |
|  |  | E4 | 0.24 | 0.04 | 6.22 | <0.001 | 0.17 | 0.32 | 0.35 | 0.07 | 5.19 | <0.001 | 0.21 | 0.48 |
|  |  | E5 | 0.47 | 0.05 | 8.94 | <0.001 | 0.37 | 0.58 | 0.38 | 0.06 | 6.65 | <0.001 | 0.27 | 0.49 |
| Physical  Problems |  | F1 | 0.64 | 0.07 | 9.64 | <0.001 | 0.51 | 0.77 | 0.28 | 0.06 | 4.44 | <0.001 | 0.16 | 0.41 |
|  |  | F2 | 0.55 | 0.06 | 8.76 | <0.001 | 0.43 | 0.67 | 0.32 | 0.06 | 5.32 | <0.001 | 0.20 | 0.44 |
|  |  | F3 | 0.41 | 0.05 | 8.19 | <0.001 | 0.31 | 0.51 | 0.63 | 0.08 | 7.98 | <0.001 | 0.47 | 0.78 |
|  |  | F4 | 0.69 | 0.05 | 13.54 | <0.001 | 0.59 | 0.79 | 0.54 | 0.08 | 6.78 | <0.001 | 0.39 | 0.70 |
|  |  | F5 | 0.47 | 0.06 | 8.22 | <0.001 | 0.36 | 0.58 | 0.33 | 0.07 | 4.79 | <0.001 | 0.19 | 0.46 |

*Note*. Group: (1) general population sample, (2) TBI sample, Estimate: variance, *SE*: standard error, *z*: *z*-value, *p*: *p*-value, 95% CI: 95% confidence interval.

Table S15. Standardized Factor Covariances for Measurement Invariance Model 1 (Configural Invariance) for QoLIBRI Scales (MLR Estimator)

| Scale 1 | Scale 2 | Group | Estimate | *SE* | *z* | *p* | 95% CI lower | 95% CI upper |
| --- | --- | --- | --- | --- | --- | --- | --- | --- |
| Cognition | Self | 1 | 0.68 | 0.05 | 15.08 | <0.001 | 0.60 | 0.77 |
|  | Daily Life and Autonomy | 1 | 0.68 | 0.05 | 13.97 | <0.001 | 0.58 | 0.77 |
|  | Social Relationships | 1 | 0.53 | 0.06 | 8.37 | <0.001 | 0.41 | 0.66 |
|  | Emotional Problems | 1 | 0.45 | 0.06 | 7.92 | <0.001 | 0.34 | 0.57 |
|  | Physical Problems | 1 | 0.37 | 0.07 | 5.41 | <0.001 | 0.24 | 0.51 |
| Self | Daily Life and Autonomy | 1 | 0.79 | 0.04 | 19.55 | <0.001 | 0.71 | 0.87 |
|  | Social Relationships | 1 | 0.69 | 0.05 | 14.16 | <0.001 | 0.60 | 0.79 |
|  | Emotional Problems | 1 | 0.42 | 0.06 | 6.51 | <0.001 | 0.29 | 0.54 |
|  | Physical Problems | 1 | 0.28 | 0.07 | 3.92 | <0.001 | 0.14 | 0.42 |
| Daily Life and  Autonomy | Social Relationships | 1 | 0.78 | 0.04 | 20.08 | <0.001 | 0.71 | 0.86 |
|  | Emotional Problems | 1 | 0.47 | 0.06 | 7.65 | <0.001 | 0.35 | 0.59 |
|  | Physical Problems | 1 | 0.37 | 0.07 | 5.39 | <0.001 | 0.24 | 0.51 |
| Social Relationships | Emotional Problems | 1 | 0.33 | 0.07 | 4.78 | <0.001 | 0.19 | 0.47 |
|  | Physical Problems | 1 | 0.18 | 0.07 | 2.39 | 0.017 | 0.03 | 0.32 |
| Emotional Problems | Physical Problems | 1 | 0.70 | 0.04 | 16.26 | <0.001 | 0.62 | 0.79 |
| Cognition | Self | 2 | 0.84 | 0.03 | 24.10 | <0.001 | 0.77 | 0.91 |
|  | Daily Life and Autonomy | 2 | 0.81 | 0.04 | 20.15 | <0.001 | 0.73 | 0.89 |
|  | Social Relationships | 2 | 0.63 | 0.07 | 8.59 | <0.001 | 0.49 | 0.78 |
|  | Emotional Problems | 2 | -0.30 | 0.09 | -3.28 | 0.001 | -0.49 | -0.12 |
|  | Physical Problems | 2 | -0.09 | 0.10 | -0.94 | 0.346 | -0.29 | 0.10 |
| Self | Daily Life and Autonomy | 2 | 0.88 | 0.03 | 33.92 | <0.001 | 0.82 | 0.93 |
|  | Social Relationships | 2 | 0.86 | 0.03 | 27.92 | <0.001 | 0.80 | 0.92 |
|  | Emotional Problems | 2 | -0.23 | 0.10 | -2.28 | 0.022 | -0.42 | -0.03 |
|  | Physical Problems | 2 | -0.15 | 0.10 | -1.43 | 0.152 | -0.35 | 0.05 |
| Daily Life and  Autonomy | Social Relationships | 2 | 0.67 | 0.07 | 10.02 | <0.001 | 0.54 | 0.80 |
|  | Emotional Problems | 2 | -0.20 | 0.10 | -2.02 | 0.043 | -0.38 | -0.01 |
|  | Physical Problems | 2 | -0.08 | 0.10 | -0.76 | 0.444 | -0.27 | 0.12 |
| Social Relationships | Emotional Problems | 2 | -0.19 | 0.10 | -2.02 | 0.043 | -0.38 | -0.01 |
|  | Physical Problems | 2 | -0.24 | 0.09 | -2.67 | 0.008 | -0.42 | -0.06 |
| Emotional Problems | Physical Problems | 2 | 0.63 | 0.08 | 8.38 | <0.001 | 0.48 | 0.78 |

*Note*. Group: (1) general population sample, (2) TBI sample, Estimate: covariance, *SE*: standard error, *z*: *z*-value, *p*: *p*-value, 95% CI: 95% confidence interval.

### Model 2

Table S16. Standardized Factor Loadings for Measurement Invariance Model 2 (Metric Invariance) for QOLIBRI (MLR Estimator)

|  |  | Group 1 | | | | | | Group 2 | | | | | |
| --- | --- | --- | --- | --- | --- | --- | --- | --- | --- | --- | --- | --- | --- |
| Scale | Items | Estimate | *SE* | *z* | *p* | 95% CI lower | 95% CI upper | Estimate | *SE* | *z* | *p* | 95% CI lower | 95% CI upper |
| Cognition | A1 | 0.73 | 0.04 | 19.55 | <0.001 | 0.65 | 0.80 | 0.87 | 0.03 | 33.55 | <0.001 | 0.82 | 0.92 |
|  | A2 | 0.68 | 0.03 | 19.58 | <0.001 | 0.62 | 0.75 | 0.83 | 0.04 | 20.74 | <0.001 | 0.75 | 0.91 |
|  | A3 | 0.70 | 0.03 | 20.97 | <0.001 | 0.63 | 0.76 | 0.77 | 0.04 | 19.76 | <0.001 | 0.69 | 0.84 |
|  | A4 | 0.78 | 0.03 | 28.37 | <0.001 | 0.72 | 0.83 | 0.83 | 0.03 | 28.35 | <0.001 | 0.77 | 0.89 |
|  | A5 | 0.65 | 0.04 | 17.34 | <0.001 | 0.57 | 0.72 | 0.77 | 0.04 | 17.82 | <0.001 | 0.69 | 0.86 |
|  | A6 | 0.57 | 0.04 | 13.33 | <0.001 | 0.49 | 0.66 | 0.71 | 0.05 | 13.69 | <0.001 | 0.61 | 0.82 |
|  | A7 | 0.78 | 0.03 | 27.40 | <0.001 | 0.72 | 0.83 | 0.86 | 0.03 | 26.77 | <0.001 | 0.80 | 0.92 |
| Self | B1 | 0.69 | 0.03 | 22.52 | <0.001 | 0.63 | 0.75 | 0.77 | 0.04 | 20.34 | <0.001 | 0.70 | 0.85 |
|  | B2 | 0.68 | 0.03 | 19.54 | <0.001 | 0.62 | 0.75 | 0.79 | 0.03 | 25.92 | <0.001 | 0.73 | 0.85 |
|  | B3 | 0.80 | 0.02 | 36.58 | <0.001 | 0.75 | 0.84 | 0.84 | 0.03 | 29.02 | <0.001 | 0.78 | 0.89 |
|  | B4 | 0.64 | 0.03 | 18.21 | <0.001 | 0.57 | 0.70 | 0.67 | 0.04 | 15.44 | <0.001 | 0.59 | 0.76 |
|  | B5 | 0.77 | 0.03 | 26.71 | <0.001 | 0.71 | 0.82 | 0.80 | 0.03 | 27.61 | <0.001 | 0.75 | 0.86 |
|  | B6 | 0.86 | 0.02 | 39.11 | <0.001 | 0.81 | 0.90 | 0.85 | 0.02 | 36.46 | <0.001 | 0.81 | 0.90 |
|  | B7 | 0.73 | 0.03 | 28.89 | <0.001 | 0.68 | 0.78 | 0.85 | 0.03 | 32.53 | <0.001 | 0.79 | 0.90 |
| Daily Life  and  Autonomy | C1 | 0.67 | 0.03 | 21.84 | <0.001 | 0.61 | 0.73 | 0.84 | 0.04 | 19.85 | <0.001 | 0.76 | 0.92 |
|  | C2 | 0.69 | 0.04 | 19.38 | <0.001 | 0.62 | 0.76 | 0.82 | 0.04 | 21.17 | <0.001 | 0.75 | 0.90 |
|  | C3 | 0.64 | 0.04 | 17.20 | <0.001 | 0.57 | 0.72 | 0.72 | 0.04 | 19.23 | <0.001 | 0.65 | 0.80 |
|  | C4 | 0.57 | 0.04 | 14.18 | <0.001 | 0.49 | 0.65 | 0.68 | 0.05 | 13.71 | <0.001 | 0.59 | 0.78 |
|  | C5 | 0.63 | 0.04 | 16.87 | <0.001 | 0.56 | 0.70 | 0.73 | 0.04 | 16.79 | <0.001 | 0.64 | 0.81 |
|  | C6 | 0.66 | 0.03 | 19.23 | <0.001 | 0.59 | 0.72 | 0.78 | 0.04 | 21.11 | <0.001 | 0.71 | 0.85 |
|  | C7 | 0.78 | 0.02 | 31.76 | <0.001 | 0.73 | 0.82 | 0.89 | 0.02 | 40.47 | <0.001 | 0.84 | 0.93 |
| Social  Relationships | D1 | 0.71 | 0.03 | 21.55 | <0.001 | 0.64 | 0.77 | 0.79 | 0.04 | 18.34 | <0.001 | 0.71 | 0.88 |
|  | D2 | 0.66 | 0.04 | 17.02 | <0.001 | 0.58 | 0.73 | 0.76 | 0.05 | 14.94 | <0.001 | 0.66 | 0.86 |
|  | D3 | 0.71 | 0.03 | 20.38 | <0.001 | 0.64 | 0.78 | 0.80 | 0.04 | 20.19 | <0.001 | 0.72 | 0.88 |
|  | D4 | 0.61 | 0.04 | 15.98 | <0.001 | 0.54 | 0.69 | 0.68 | 0.05 | 14.38 | <0.001 | 0.59 | 0.77 |
|  | D5 | 0.61 | 0.04 | 16.96 | <0.001 | 0.54 | 0.68 | 0.67 | 0.05 | 12.95 | <0.001 | 0.57 | 0.78 |
|  | D6 | 0.70 | 0.04 | 18.25 | <0.001 | 0.62 | 0.77 | 0.74 | 0.04 | 18.81 | <0.001 | 0.67 | 0.82 |
| Emotional  Problems | E1 | 0.68 | 0.03 | 23.25 | <0.001 | 0.62 | 0.74 | 0.84 | 0.04 | 21.32 | <0.001 | 0.76 | 0.91 |
|  | E2 | 0.64 | 0.03 | 18.70 | <0.001 | 0.57 | 0.71 | 0.75 | 0.05 | 16.28 | <0.001 | 0.66 | 0.84 |
|  | E3 | 0.81 | 0.03 | 29.63 | <0.001 | 0.75 | 0.86 | 0.84 | 0.03 | 27.84 | <0.001 | 0.78 | 0.90 |
|  | E4 | 0.84 | 0.03 | 33.32 | <0.001 | 0.79 | 0.89 | 0.85 | 0.03 | 31.70 | <0.001 | 0.80 | 0.91 |
|  | E5 | 0.73 | 0.03 | 23.97 | <0.001 | 0.67 | 0.79 | 0.77 | 0.04 | 21.89 | <0.001 | 0.70 | 0.84 |
| Physical  Problems | F1 | 0.67 | 0.03 | 19.65 | <0.001 | 0.61 | 0.74 | 0.80 | 0.05 | 16.10 | <0.001 | 0.70 | 0.89 |
|  | F2 | 0.71 | 0.03 | 22.17 | <0.001 | 0.65 | 0.78 | 0.79 | 0.04 | 18.08 | <0.001 | 0.70 | 0.87 |
|  | F3 | 0.69 | 0.04 | 17.87 | <0.001 | 0.62 | 0.77 | 0.72 | 0.03 | 20.99 | <0.001 | 0.66 | 0.79 |
|  | F4 | 0.56 | 0.04 | 14.60 | <0.001 | 0.49 | 0.64 | 0.67 | 0.05 | 14.33 | <0.001 | 0.58 | 0.76 |
|  | F5 | 0.69 | 0.04 | 19.57 | <0.001 | 0.62 | 0.76 | 0.83 | 0.04 | 23.26 | <0.001 | 0.76 | 0.90 |

*Note*. Group: (1) general population sample, (2) TBI sample, Estimate: factor loading, *SE*: standard error, *z*: *z*-value, *p*: *p*-value, 95% CI: 95% confidence interval.

Table S17. Standardized Factor Variances for Measurement Invariance Model 2 (Metric Invariance) for QoLIBRI Scales (MLR Estimator)

|  |  |  | Group 1 | | | | | | Group 2 | | | | | |
| --- | --- | --- | --- | --- | --- | --- | --- | --- | --- | --- | --- | --- | --- | --- |
| Scale |  | Items | Estimate | *SE* | *z* | *p* | 95% CI lower | 95% CI upper | Estimate | *SE* | *z* | *p* | 95% CI lower | 95% CI upper |
| Cognition |  | A1 | 0.47 | 0.05 | 8.79 | <0.001 | 0.37 | 0.58 | 0.24 | 0.05 | 5.29 | <0.001 | 0.15 | 0.33 |
|  |  | A2 | 0.53 | 0.05 | 11.13 | <0.001 | 0.44 | 0.63 | 0.31 | 0.07 | 4.72 | <0.001 | 0.18 | 0.44 |
|  |  | A3 | 0.51 | 0.05 | 11.09 | <0.001 | 0.42 | 0.60 | 0.41 | 0.06 | 6.93 | <0.001 | 0.30 | 0.53 |
|  |  | A4 | 0.40 | 0.04 | 9.38 | <0.001 | 0.31 | 0.48 | 0.31 | 0.05 | 6.47 | <0.001 | 0.22 | 0.41 |
|  |  | A5 | 0.58 | 0.05 | 11.99 | <0.001 | 0.49 | 0.68 | 0.40 | 0.07 | 6.01 | <0.001 | 0.27 | 0.53 |
|  |  | A6 | 0.67 | 0.05 | 13.68 | <0.001 | 0.58 | 0.77 | 0.49 | 0.07 | 6.57 | <0.001 | 0.34 | 0.64 |
|  |  | A7 | 0.40 | 0.04 | 9.05 | <0.001 | 0.31 | 0.48 | 0.26 | 0.06 | 4.77 | <0.001 | 0.15 | 0.37 |
| Self |  | B1 | 0.52 | 0.04 | 12.30 | <0.001 | 0.44 | 0.61 | 0.40 | 0.06 | 6.82 | <0.001 | 0.29 | 0.52 |
|  |  | B2 | 0.53 | 0.05 | 11.12 | <0.001 | 0.44 | 0.63 | 0.37 | 0.05 | 7.70 | <0.001 | 0.28 | 0.47 |
|  |  | B3 | 0.37 | 0.03 | 10.54 | <0.001 | 0.30 | 0.43 | 0.30 | 0.05 | 6.28 | <0.001 | 0.21 | 0.40 |
|  |  | B4 | 0.60 | 0.04 | 13.42 | <0.001 | 0.51 | 0.68 | 0.54 | 0.06 | 9.22 | <0.001 | 0.43 | 0.66 |
|  |  | B5 | 0.41 | 0.04 | 9.28 | <0.001 | 0.32 | 0.50 | 0.36 | 0.05 | 7.65 | <0.001 | 0.27 | 0.45 |
|  |  | B6 | 0.27 | 0.04 | 7.08 | <0.001 | 0.19 | 0.34 | 0.28 | 0.04 | 6.95 | <0.001 | 0.20 | 0.35 |
|  |  | B7 | 0.46 | 0.04 | 12.40 | <0.001 | 0.39 | 0.53 | 0.28 | 0.04 | 6.48 | <0.001 | 0.20 | 0.37 |
| Daily Life  and  Autonomy |  | C1 | 0.55 | 0.04 | 13.17 | <0.001 | 0.47 | 0.63 | 0.29 | 0.07 | 4.08 | <0.001 | 0.15 | 0.43 |
|  |  | C2 | 0.52 | 0.05 | 10.57 | <0.001 | 0.42 | 0.62 | 0.32 | 0.06 | 5.05 | <0.001 | 0.20 | 0.45 |
|  |  | C3 | 0.59 | 0.05 | 12.23 | <0.001 | 0.49 | 0.68 | 0.47 | 0.05 | 8.70 | <0.001 | 0.37 | 0.58 |
|  |  | C4 | 0.67 | 0.05 | 14.43 | <0.001 | 0.58 | 0.76 | 0.53 | 0.07 | 7.84 | <0.001 | 0.40 | 0.67 |
|  |  | C5 | 0.60 | 0.05 | 12.84 | <0.001 | 0.51 | 0.70 | 0.47 | 0.06 | 7.42 | <0.001 | 0.35 | 0.59 |
|  |  | C6 | 0.57 | 0.05 | 12.60 | <0.001 | 0.48 | 0.66 | 0.39 | 0.06 | 6.88 | <0.001 | 0.28 | 0.51 |
|  |  | C7 | 0.40 | 0.04 | 10.53 | <0.001 | 0.32 | 0.47 | 0.21 | 0.04 | 5.48 | <0.001 | 0.14 | 0.29 |
| Social Relationships |  | D1 | 0.50 | 0.05 | 10.77 | <0.001 | 0.41 | 0.59 | 0.37 | 0.07 | 5.46 | <0.001 | 0.24 | 0.51 |
|  |  | D2 | 0.57 | 0.05 | 11.30 | <0.001 | 0.47 | 0.67 | 0.42 | 0.08 | 5.51 | <0.001 | 0.27 | 0.58 |
|  |  | D3 | 0.49 | 0.05 | 9.90 | <0.001 | 0.40 | 0.59 | 0.36 | 0.06 | 5.65 | <0.001 | 0.23 | 0.48 |
|  |  | D4 | 0.63 | 0.05 | 13.33 | <0.001 | 0.53 | 0.72 | 0.54 | 0.06 | 8.47 | <0.001 | 0.42 | 0.67 |
|  |  | D5 | 0.62 | 0.04 | 14.02 | <0.001 | 0.54 | 0.71 | 0.55 | 0.07 | 7.81 | <0.001 | 0.41 | 0.68 |
|  |  | D6 | 0.51 | 0.05 | 9.53 | <0.001 | 0.41 | 0.62 | 0.45 | 0.06 | 7.58 | <0.001 | 0.33 | 0.56 |
| Emotional Problems |  | E1 | 0.53 | 0.04 | 13.35 | <0.001 | 0.46 | 0.61 | 0.30 | 0.07 | 4.56 | <0.001 | 0.17 | 0.43 |
|  |  | E2 | 0.59 | 0.04 | 13.60 | <0.001 | 0.51 | 0.68 | 0.43 | 0.07 | 6.24 | <0.001 | 0.30 | 0.57 |
|  |  | E3 | 0.35 | 0.04 | 8.05 | <0.001 | 0.27 | 0.44 | 0.29 | 0.05 | 5.70 | <0.001 | 0.19 | 0.39 |
|  |  | E4 | 0.29 | 0.04 | 6.71 | <0.001 | 0.20 | 0.37 | 0.27 | 0.05 | 5.90 | <0.001 | 0.18 | 0.36 |
|  |  | E5 | 0.46 | 0.04 | 10.37 | <0.001 | 0.38 | 0.55 | 0.41 | 0.05 | 7.65 | <0.001 | 0.31 | 0.52 |
| Physical  Problems |  | F1 | 0.55 | 0.05 | 11.81 | <0.001 | 0.46 | 0.64 | 0.37 | 0.08 | 4.63 | <0.001 | 0.21 | 0.52 |
|  |  | F2 | 0.49 | 0.05 | 10.76 | <0.001 | 0.40 | 0.58 | 0.38 | 0.07 | 5.54 | <0.001 | 0.25 | 0.51 |
|  |  | F3 | 0.52 | 0.05 | 9.76 | <0.001 | 0.42 | 0.63 | 0.48 | 0.05 | 9.55 | <0.001 | 0.38 | 0.57 |
|  |  | F4 | 0.68 | 0.04 | 15.67 | <0.001 | 0.60 | 0.77 | 0.55 | 0.06 | 8.70 | <0.001 | 0.42 | 0.67 |
|  |  | F5 | 0.52 | 0.05 | 10.55 | <0.001 | 0.42 | 0.62 | 0.31 | 0.06 | 5.15 | <0.001 | 0.19 | 0.42 |

*Note*. Group: (1) general population sample, (2) TBI sample, Estimate: variance, *SE*: standard error, *z*: *z*-value, *p*: *p*-value, 95% CI: 95% confidence interval.

Table S18. Standardized factor Covariances for Measurement Invariance Model 2 (Metric Invariance) for QoLIBRI Scales (MLR Estimator)

| Scale 1 | Scale 2 | Group | Estimate | *SE* | *z* | *p* | 95% CI lower | 95% CI upper |
| --- | --- | --- | --- | --- | --- | --- | --- | --- |
| Cognition | Self | 1 | 0.69 | 0.04 | 15.35 | <0.001 | 0.60 | 0.78 |
|  | Daily Life and Autonomy | 1 | 0.68 | 0.05 | 14.02 | <0.001 | 0.58 | 0.77 |
|  | Social Relationships | 1 | 0.53 | 0.06 | 8.44 | <0.001 | 0.41 | 0.65 |
|  | Emotional Problems | 1 | 0.45 | 0.06 | 7.90 | <0.001 | 0.34 | 0.56 |
|  | Physical Problems | 1 | 0.38 | 0.07 | 5.64 | <0.001 | 0.25 | 0.51 |
| Self | Daily Life and Autonomy | 1 | 0.79 | 0.04 | 19.81 | <0.001 | 0.71 | 0.87 |
|  | Social Relationships | 1 | 0.69 | 0.05 | 14.32 | <0.001 | 0.60 | 0.79 |
|  | Emotional Problems | 1 | 0.41 | 0.06 | 6.43 | <0.001 | 0.29 | 0.54 |
|  | Physical Problems | 1 | 0.27 | 0.07 | 3.77 | <0.001 | 0.13 | 0.40 |
| Daily Life and  Autonomy | Social Relationships | 1 | 0.78 | 0.04 | 20.42 | <0.001 | 0.71 | 0.86 |
|  | Emotional Problems | 1 | 0.46 | 0.06 | 7.57 | <0.001 | 0.34 | 0.58 |
|  | Physical Problems | 1 | 0.37 | 0.07 | 5.41 | <0.001 | 0.24 | 0.51 |
| Social Relationships | Emotional Problems | 1 | 0.33 | 0.07 | 4.73 | <0.001 | 0.19 | 0.46 |
|  | Physical Problems | 1 | 0.18 | 0.07 | 2.51 | 0.012 | 0.04 | 0.33 |
| Emotional Problems | Physical Problems | 1 | 0.71 | 0.04 | 16.90 | <0.001 | 0.63 | 0.79 |
| Cognition | Self | 2 | 0.84 | 0.04 | 23.87 | <0.001 | 0.77 | 0.91 |
|  | Daily Life and Autonomy | 2 | 0.82 | 0.04 | 20.75 | <0.001 | 0.74 | 0.89 |
|  | Social Relationships | 2 | 0.63 | 0.07 | 8.75 | <0.001 | 0.49 | 0.78 |
|  | Emotional Problems | 2 | -0.30 | 0.09 | -3.22 | 0.001 | -0.48 | -0.12 |
|  | Physical Problems | 2 | -0.09 | 0.10 | -0.91 | 0.365 | -0.29 | 0.11 |
| Self | Daily Life and Autonomy | 2 | 0.87 | 0.03 | 33.81 | <0.001 | 0.82 | 0.92 |
|  | Social Relationships | 2 | 0.86 | 0.03 | 28.30 | <0.001 | 0.80 | 0.92 |
|  | Emotional Problems | 2 | -0.22 | 0.10 | -2.23 | 0.026 | -0.42 | -0.03 |
|  | Physical Problems | 2 | -0.14 | 0.10 | -1.41 | 0.159 | -0.35 | 0.06 |
| Daily Life and  Autonomy | Social Relationships | 2 | 0.67 | 0.07 | 10.21 | <0.001 | 0.54 | 0.80 |
|  | Emotional Problems | 2 | -0.20 | 0.10 | -2.07 | 0.039 | -0.39 | -0.01 |
|  | Physical Problems | 2 | -0.08 | 0.10 | -0.79 | 0.431 | -0.27 | 0.11 |
| Social Relationships | Emotional Problems | 2 | -0.19 | 0.10 | -1.98 | 0.047 | -0.38 | 0.00 |
|  | Physical Problems | 2 | -0.24 | 0.09 | -2.62 | 0.009 | -0.42 | -0.06 |
| Emotional Problems | Physical Problems | 2 | 0.64 | 0.08 | 8.43 | <0.001 | 0.49 | 0.78 |

*Note*. Group: (1) general population sample, (2) TBI sample, Estimate: covariance, *SE*: standard error, *z*: *z*-value, *p*: *p*-value, 95% CI: 95% confidence interval.

### Model 3

Table S19. Standardized Factor Loadings for Measurement Invariance Model 3 (Scalar Invariance) for QoLIBRI Scales (MLR Estimator)

|  |  | Group 1 | | | | | | Group 2 | | | | | |
| --- | --- | --- | --- | --- | --- | --- | --- | --- | --- | --- | --- | --- | --- |
| Scale | Items | Estimate | *SE* | *z* | *p* | 95% CI lower | 95% CI upper | Estimate | *SE* | *z* | *p* | 95% CI lower | 95% CI upper |
| Cognition | A1 | 0.73 | 0.04 | 19.60 | <0.001 | 0.65 | 0.80 | 0.87 | 0.03 | 34.10 | <0.001 | 0.82 | 0.92 |
|  | A2 | 0.68 | 0.04 | 19.07 | <0.001 | 0.61 | 0.75 | 0.83 | 0.04 | 20.23 | <0.001 | 0.75 | 0.91 |
|  | A3 | 0.70 | 0.03 | 21.03 | <0.001 | 0.63 | 0.76 | 0.77 | 0.04 | 19.72 | <0.001 | 0.69 | 0.84 |
|  | A4 | 0.77 | 0.03 | 27.78 | <0.001 | 0.72 | 0.83 | 0.82 | 0.03 | 27.63 | <0.001 | 0.77 | 0.88 |
|  | A5 | 0.64 | 0.04 | 16.74 | <0.001 | 0.57 | 0.72 | 0.77 | 0.04 | 17.90 | <0.001 | 0.68 | 0.85 |
|  | A6 | 0.58 | 0.04 | 13.57 | <0.001 | 0.49 | 0.66 | 0.72 | 0.05 | 13.98 | <0.001 | 0.62 | 0.82 |
|  | A7 | 0.78 | 0.03 | 28.01 | <0.001 | 0.72 | 0.83 | 0.86 | 0.03 | 26.41 | <0.001 | 0.80 | 0.92 |
| Self | B1 | 0.69 | 0.03 | 22.15 | <0.001 | 0.63 | 0.75 | 0.77 | 0.04 | 19.17 | <0.001 | 0.69 | 0.85 |
|  | B2 | 0.68 | 0.03 | 19.66 | <0.001 | 0.62 | 0.75 | 0.79 | 0.03 | 26.12 | <0.001 | 0.73 | 0.85 |
|  | B3 | 0.79 | 0.02 | 35.70 | <0.001 | 0.75 | 0.84 | 0.82 | 0.03 | 27.07 | <0.001 | 0.76 | 0.88 |
|  | B4 | 0.64 | 0.03 | 18.24 | <0.001 | 0.57 | 0.71 | 0.67 | 0.04 | 15.70 | <0.001 | 0.58 | 0.75 |
|  | B5 | 0.77 | 0.03 | 26.72 | <0.001 | 0.71 | 0.83 | 0.78 | 0.03 | 23.49 | <0.001 | 0.71 | 0.84 |
|  | B6 | 0.86 | 0.02 | 38.96 | <0.001 | 0.81 | 0.90 | 0.85 | 0.02 | 35.90 | <0.001 | 0.80 | 0.90 |
|  | B7 | 0.73 | 0.03 | 29.08 | <0.001 | 0.68 | 0.78 | 0.85 | 0.03 | 32.20 | <0.001 | 0.79 | 0.90 |
| Daily Life  and  Autonomy | C1 | 0.67 | 0.03 | 21.93 | <0.001 | 0.61 | 0.73 | 0.84 | 0.04 | 19.63 | <0.001 | 0.76 | 0.92 |
|  | C2 | 0.69 | 0.04 | 19.36 | <0.001 | 0.62 | 0.76 | 0.82 | 0.04 | 21.18 | <0.001 | 0.75 | 0.90 |
|  | C3 | 0.64 | 0.04 | 17.20 | <0.001 | 0.57 | 0.72 | 0.72 | 0.04 | 19.26 | <0.001 | 0.65 | 0.80 |
|  | C4 | 0.57 | 0.04 | 14.20 | <0.001 | 0.49 | 0.65 | 0.68 | 0.05 | 13.64 | <0.001 | 0.58 | 0.78 |
|  | C5 | 0.63 | 0.04 | 16.95 | <0.001 | 0.56 | 0.70 | 0.73 | 0.04 | 16.40 | <0.001 | 0.64 | 0.81 |
|  | C6 | 0.66 | 0.03 | 19.40 | <0.001 | 0.59 | 0.72 | 0.78 | 0.04 | 21.02 | <0.001 | 0.71 | 0.85 |
|  | C7 | 0.78 | 0.02 | 31.68 | <0.001 | 0.73 | 0.82 | 0.89 | 0.02 | 40.16 | <0.001 | 0.84 | 0.93 |
| Social Relationships | D1 | 0.70 | 0.03 | 21.45 | <0.001 | 0.64 | 0.77 | 0.79 | 0.04 | 18.46 | <0.001 | 0.70 | 0.87 |
|  | D2 | 0.66 | 0.04 | 17.10 | <0.001 | 0.58 | 0.73 | 0.76 | 0.05 | 14.82 | <0.001 | 0.66 | 0.86 |
|  | D3 | 0.71 | 0.03 | 20.46 | <0.001 | 0.64 | 0.78 | 0.80 | 0.04 | 20.09 | <0.001 | 0.72 | 0.88 |
|  | D4 | 0.61 | 0.04 | 16.11 | <0.001 | 0.54 | 0.69 | 0.68 | 0.05 | 14.23 | <0.001 | 0.58 | 0.77 |
|  | D5 | 0.61 | 0.04 | 17.03 | <0.001 | 0.54 | 0.68 | 0.67 | 0.05 | 12.55 | <0.001 | 0.57 | 0.78 |
|  | D6 | 0.70 | 0.04 | 18.31 | <0.001 | 0.63 | 0.78 | 0.74 | 0.04 | 18.81 | <0.001 | 0.67 | 0.82 |
| Emotional Problems | E1 | 0.68 | 0.03 | 23.84 | <0.001 | 0.63 | 0.74 | 0.84 | 0.04 | 21.72 | <0.001 | 0.76 | 0.91 |
|  | E2 | 0.64 | 0.03 | 20.55 | <0.001 | 0.58 | 0.70 | 0.76 | 0.04 | 17.43 | <0.001 | 0.67 | 0.84 |
|  | E3 | 0.80 | 0.03 | 29.58 | <0.001 | 0.75 | 0.85 | 0.84 | 0.03 | 27.12 | <0.001 | 0.78 | 0.90 |
|  | E4 | 0.84 | 0.03 | 33.24 | <0.001 | 0.79 | 0.89 | 0.85 | 0.03 | 32.39 | <0.001 | 0.80 | 0.90 |
|  | E5 | 0.74 | 0.03 | 26.47 | <0.001 | 0.69 | 0.80 | 0.77 | 0.03 | 23.03 | <0.001 | 0.71 | 0.84 |
| Physical  Problems | F1 | 0.69 | 0.03 | 22.30 | <0.001 | 0.63 | 0.75 | 0.81 | 0.04 | 18.15 | <0.001 | 0.72 | 0.90 |
|  | F2 | 0.71 | 0.03 | 23.39 | <0.001 | 0.65 | 0.77 | 0.79 | 0.04 | 19.11 | <0.001 | 0.71 | 0.87 |
|  | F3 | 0.66 | 0.04 | 16.81 | <0.001 | 0.58 | 0.74 | 0.70 | 0.04 | 19.80 | <0.001 | 0.63 | 0.77 |
|  | F4 | 0.57 | 0.04 | 15.91 | <0.001 | 0.50 | 0.64 | 0.68 | 0.05 | 14.94 | <0.001 | 0.59 | 0.77 |
|  | F5 | 0.69 | 0.03 | 20.22 | <0.001 | 0.62 | 0.75 | 0.83 | 0.03 | 24.67 | <0.001 | 0.76 | 0.90 |

*Note*. Group: (1) general population sample, (2) TBI sample, Estimate: factor loading, *SE*: standard error, *z*: *z*-value, *p*: *p*-value, 95% CI: 95% confidence interval.

Table S20. Standardized Factor Variances for Measurement Invariance Model 3 (Scalar Invariance) for QoLIBRI Scales (MLR Estimator)

|  |  |  | Group 1 | | | | | | Group 2 | | | | | |
| --- | --- | --- | --- | --- | --- | --- | --- | --- | --- | --- | --- | --- | --- | --- |
| Scale |  | Items | Estimate | *SE* | *z* | *p* | 95% CI lower | 95% CI upper | Estimate | *SE* | *z* | *p* | 95% CI lower | 95% CI upper |
| Cognition |  | A1 | 0.47 | 0.05 | 8.79 | <0.001 | 0.37 | 0.58 | 0.24 | 0.04 | 5.34 | <0.001 | 0.15 | 0.33 |
|  |  | A2 | 0.54 | 0.05 | 11.03 | <0.001 | 0.44 | 0.63 | 0.32 | 0.07 | 4.70 | <0.001 | 0.19 | 0.45 |
|  |  | A3 | 0.51 | 0.05 | 11.06 | <0.001 | 0.42 | 0.60 | 0.41 | 0.06 | 6.88 | <0.001 | 0.29 | 0.53 |
|  |  | A4 | 0.40 | 0.04 | 9.39 | <0.001 | 0.32 | 0.49 | 0.32 | 0.05 | 6.49 | <0.001 | 0.22 | 0.42 |
|  |  | A5 | 0.59 | 0.05 | 11.85 | <0.001 | 0.49 | 0.68 | 0.41 | 0.07 | 6.23 | <0.001 | 0.28 | 0.54 |
|  |  | A6 | 0.67 | 0.05 | 13.64 | <0.001 | 0.57 | 0.76 | 0.49 | 0.07 | 6.62 | <0.001 | 0.34 | 0.63 |
|  |  | A7 | 0.39 | 0.04 | 9.07 | <0.001 | 0.31 | 0.48 | 0.26 | 0.06 | 4.69 | <0.001 | 0.15 | 0.37 |
| Self |  | B1 | 0.53 | 0.04 | 12.28 | <0.001 | 0.44 | 0.61 | 0.41 | 0.06 | 6.64 | <0.001 | 0.29 | 0.53 |
|  |  | B2 | 0.53 | 0.05 | 11.19 | <0.001 | 0.44 | 0.63 | 0.37 | 0.05 | 7.70 | <0.001 | 0.28 | 0.47 |
|  |  | B3 | 0.37 | 0.04 | 10.62 | <0.001 | 0.30 | 0.44 | 0.33 | 0.05 | 6.54 | <0.001 | 0.23 | 0.42 |
|  |  | B4 | 0.59 | 0.04 | 13.37 | <0.001 | 0.51 | 0.68 | 0.55 | 0.06 | 9.78 | <0.001 | 0.44 | 0.67 |
|  |  | B5 | 0.41 | 0.04 | 9.15 | <0.001 | 0.32 | 0.49 | 0.39 | 0.05 | 7.65 | <0.001 | 0.29 | 0.50 |
|  |  | B6 | 0.27 | 0.04 | 7.07 | <0.001 | 0.19 | 0.34 | 0.28 | 0.04 | 6.95 | <0.001 | 0.20 | 0.36 |
|  |  | B7 | 0.46 | 0.04 | 12.48 | <0.001 | 0.39 | 0.53 | 0.28 | 0.04 | 6.39 | <0.001 | 0.20 | 0.37 |
| Daily Life  and  Autonomy |  | C1 | 0.55 | 0.04 | 13.26 | <0.001 | 0.47 | 0.63 | 0.29 | 0.07 | 4.07 | <0.001 | 0.15 | 0.43 |
|  |  | C2 | 0.52 | 0.05 | 10.55 | <0.001 | 0.42 | 0.62 | 0.32 | 0.06 | 5.06 | <0.001 | 0.20 | 0.45 |
|  |  | C3 | 0.59 | 0.05 | 12.22 | <0.001 | 0.49 | 0.68 | 0.48 | 0.05 | 8.72 | <0.001 | 0.37 | 0.58 |
|  |  | C4 | 0.67 | 0.05 | 14.46 | <0.001 | 0.58 | 0.76 | 0.53 | 0.07 | 7.82 | <0.001 | 0.40 | 0.67 |
|  |  | C5 | 0.60 | 0.05 | 12.94 | <0.001 | 0.51 | 0.70 | 0.47 | 0.06 | 7.29 | <0.001 | 0.34 | 0.60 |
|  |  | C6 | 0.57 | 0.04 | 12.72 | <0.001 | 0.48 | 0.65 | 0.39 | 0.06 | 6.86 | <0.001 | 0.28 | 0.51 |
|  |  | C7 | 0.40 | 0.04 | 10.50 | <0.001 | 0.32 | 0.47 | 0.21 | 0.04 | 5.46 | <0.001 | 0.14 | 0.29 |
| Social Relationships |  | D1 | 0.50 | 0.05 | 10.87 | <0.001 | 0.41 | 0.59 | 0.38 | 0.07 | 5.69 | <0.001 | 0.25 | 0.51 |
|  |  | D2 | 0.57 | 0.05 | 11.37 | <0.001 | 0.47 | 0.67 | 0.43 | 0.08 | 5.48 | <0.001 | 0.27 | 0.58 |
|  |  | D3 | 0.49 | 0.05 | 9.88 | <0.001 | 0.39 | 0.59 | 0.36 | 0.06 | 5.62 | <0.001 | 0.23 | 0.48 |
|  |  | D4 | 0.63 | 0.05 | 13.47 | <0.001 | 0.53 | 0.72 | 0.54 | 0.06 | 8.43 | <0.001 | 0.42 | 0.67 |
|  |  | D5 | 0.62 | 0.04 | 14.17 | <0.001 | 0.54 | 0.71 | 0.55 | 0.07 | 7.67 | <0.001 | 0.41 | 0.69 |
|  |  | D6 | 0.51 | 0.05 | 9.47 | <0.001 | 0.40 | 0.61 | 0.45 | 0.06 | 7.61 | <0.001 | 0.33 | 0.56 |
| Emotional Problems |  | E1 | 0.53 | 0.04 | 13.61 | <0.001 | 0.46 | 0.61 | 0.30 | 0.06 | 4.59 | <0.001 | 0.17 | 0.42 |
|  |  | E2 | 0.59 | 0.04 | 14.73 | <0.001 | 0.51 | 0.67 | 0.43 | 0.07 | 6.53 | <0.001 | 0.30 | 0.56 |
|  |  | E3 | 0.36 | 0.04 | 8.23 | <0.001 | 0.27 | 0.44 | 0.30 | 0.05 | 5.70 | <0.001 | 0.19 | 0.40 |
|  |  | E4 | 0.30 | 0.04 | 6.98 | <0.001 | 0.21 | 0.38 | 0.28 | 0.04 | 6.21 | <0.001 | 0.19 | 0.36 |
|  |  | E5 | 0.45 | 0.04 | 10.86 | <0.001 | 0.37 | 0.53 | 0.40 | 0.05 | 7.69 | <0.001 | 0.30 | 0.50 |
| Physical Problems |  | F1 | 0.52 | 0.04 | 12.29 | <0.001 | 0.44 | 0.61 | 0.34 | 0.07 | 4.71 | <0.001 | 0.20 | 0.48 |
|  |  | F2 | 0.49 | 0.04 | 11.46 | <0.001 | 0.41 | 0.58 | 0.38 | 0.06 | 5.89 | <0.001 | 0.25 | 0.51 |
|  |  | F3 | 0.56 | 0.05 | 10.78 | <0.001 | 0.46 | 0.66 | 0.51 | 0.05 | 10.16 | <0.001 | 0.41 | 0.60 |
|  |  | F4 | 0.67 | 0.04 | 16.18 | <0.001 | 0.59 | 0.75 | 0.53 | 0.06 | 8.52 | <0.001 | 0.41 | 0.66 |
|  |  | F5 | 0.53 | 0.05 | 11.37 | <0.001 | 0.44 | 0.62 | 0.31 | 0.06 | 5.55 | <0.001 | 0.20 | 0.42 |

*Note*. Group: (1) general population sample, (2) TBI sample, Estimate: variance, *SE*: standard error, *z*: *z*-value, *p*: *p*-value, 95% CI: 95% confidence interval.

Table S21. Standardized Factor Covariances for Measurement Invariance Model 3 (Scalar Invariance) for QoLIBRI Scales (MLR Estimator)

| Scale 1 | Scale 2 | Group | Estimate | *SE* | *z* | *p* | 95% CI lower | 95% CI upper |
| --- | --- | --- | --- | --- | --- | --- | --- | --- |
| Cognition | Self | 1 | 0.69 | 0.04 | 15.33 | <0.001 | 0.60 | 0.78 |
| Cognition | Daily Life and Autonomy | 1 | 0.68 | 0.05 | 14.02 | <0.001 | 0.58 | 0.77 |
| Cognition | Social Relationships | 1 | 0.53 | 0.06 | 8.45 | <0.001 | 0.41 | 0.65 |
| Cognition | Emotional Problems | 1 | 0.45 | 0.06 | 7.89 | <0.001 | 0.34 | 0.56 |
| Cognition | Physical Problems | 1 | 0.38 | 0.07 | 5.67 | <0.001 | 0.25 | 0.51 |
| Self | Daily Life and Autonomy | 1 | 0.79 | 0.04 | 19.82 | <0.001 | 0.72 | 0.87 |
| Self | Social Relationships | 1 | 0.69 | 0.05 | 14.35 | <0.001 | 0.60 | 0.79 |
| Self | Emotional Problems | 1 | 0.41 | 0.06 | 6.41 | <0.001 | 0.29 | 0.54 |
| Self | Physical Problems | 1 | 0.26 | 0.07 | 3.73 | <0.001 | 0.12 | 0.40 |
| Daily Life and Autonomy | Social Relationships | 1 | 0.78 | 0.04 | 20.43 | <0.001 | 0.71 | 0.86 |
| Daily Life and Autonomy | Emotional Problems | 1 | 0.46 | 0.06 | 7.56 | <0.001 | 0.34 | 0.58 |
| Daily Life and Autonomy | Physical Problems | 1 | 0.37 | 0.07 | 5.42 | <0.001 | 0.24 | 0.51 |
| Social Relationships | Emotional Problems | 1 | 0.33 | 0.07 | 4.72 | <0.001 | 0.19 | 0.46 |
| Social Relationships | Physical Problems | 1 | 0.19 | 0.07 | 2.55 | 0.011 | 0.04 | 0.33 |
| Emotional Problems | Physical Problems | 1 | 0.71 | 0.04 | 16.98 | <0.001 | 0.63 | 0.79 |
| Cognition | Self | 2 | 0.84 | 0.04 | 24.03 | <0.001 | 0.77 | 0.91 |
| Cognition | Daily Life and Autonomy | 2 | 0.82 | 0.04 | 20.71 | <0.001 | 0.74 | 0.89 |
| Cognition | Social Relationships | 2 | 0.63 | 0.07 | 8.75 | <0.001 | 0.49 | 0.78 |
| Cognition | Emotional Problems | 2 | -0.30 | 0.09 | -3.23 | 0.001 | -0.48 | -0.12 |
| Cognition | Physical Problems | 2 | -0.09 | 0.10 | -0.91 | 0.362 | -0.29 | 0.11 |
| Self | Daily Life and Autonomy | 2 | 0.88 | 0.03 | 33.94 | <0.001 | 0.83 | 0.93 |
| Self | Social Relationships | 2 | 0.86 | 0.03 | 28.98 | <0.001 | 0.81 | 0.92 |
| Self | Emotional Problems | 2 | -0.22 | 0.10 | -2.23 | 0.026 | -0.42 | -0.03 |
| Self | Physical Problems | 2 | -0.15 | 0.10 | -1.43 | 0.153 | -0.35 | 0.05 |
| Daily Life and Autonomy | Social Relationships | 2 | 0.67 | 0.07 | 10.21 | <0.001 | 0.54 | 0.80 |
| Autonomy | Emotional Problems | 2 | -0.20 | 0.10 | -2.07 | 0.039 | -0.39 | -0.01 |
| Autonomy | Physical Problems | 2 | -0.08 | 0.10 | -0.78 | 0.438 | -0.27 | 0.12 |
| Social Relationships | Emotional Problems | 2 | -0.19 | 0.10 | -1.99 | 0.047 | -0.38 | 0.00 |
|  | Physical Problems | 2 | -0.24 | 0.09 | -2.65 | 0.008 | -0.42 | -0.06 |
| Emotional Problems | Physical Problems | 2 | 0.64 | 0.07 | 8.47 | <0.001 | 0.49 | 0.78 |

*Note*. Group: (1) general population sample, (2) TBI sample, Estimate: covariance, *SE*: standard error, *z*: *z*-value, *p*: *p*-value, 95% CI: 95% confidence interval.

### Model 4

Table S22. Standardized Factor Loadings for Measurement Invariance Model 4 (Strict Invariance) for QoLIBRI Scales (MLR Estimator)

|  |  | Group 1 | | | | | | Group 2 | | | | | |
| --- | --- | --- | --- | --- | --- | --- | --- | --- | --- | --- | --- | --- | --- |
| Scale | Items | Estimate | *SE* | *z* | *p* | 95% CI lower | 95% CI upper | Estimate | *SE* | *z* | *p* | 95% CI lower | 95% CI upper |
| Cognition | A1 | 0.74 | 0.04 | 21.02 | <0.001 | 0.67 | 0.81 | 0.83 | 0.03 | 28.55 | <0.001 | 0.78 | 0.89 |
|  | A2 | 0.69 | 0.04 | 19.00 | <0.001 | 0.62 | 0.77 | 0.80 | 0.03 | 28.36 | <0.001 | 0.74 | 0.85 |
|  | A3 | 0.69 | 0.03 | 19.68 | <0.001 | 0.62 | 0.75 | 0.79 | 0.03 | 28.55 | <0.001 | 0.74 | 0.85 |
|  | A4 | 0.76 | 0.03 | 27.60 | <0.001 | 0.70 | 0.81 | 0.85 | 0.02 | 42.22 | <0.001 | 0.81 | 0.89 |
|  | A5 | 0.65 | 0.04 | 17.45 | <0.001 | 0.58 | 0.72 | 0.76 | 0.03 | 24.41 | <0.001 | 0.70 | 0.82 |
|  | A6 | 0.58 | 0.04 | 14.10 | <0.001 | 0.50 | 0.67 | 0.70 | 0.04 | 20.01 | <0.001 | 0.64 | 0.77 |
|  | A7 | 0.78 | 0.03 | 28.98 | <0.001 | 0.72 | 0.83 | 0.86 | 0.02 | 40.37 | <0.001 | 0.82 | 0.90 |
| Self | B1 | 0.69 | 0.03 | 22.28 | <0.001 | 0.63 | 0.76 | 0.75 | 0.03 | 23.55 | <0.001 | 0.69 | 0.82 |
|  | B2 | 0.70 | 0.03 | 21.94 | <0.001 | 0.64 | 0.77 | 0.76 | 0.03 | 27.64 | <0.001 | 0.71 | 0.81 |
|  | B3 | 0.79 | 0.02 | 37.02 | <0.001 | 0.74 | 0.83 | 0.83 | 0.02 | 42.20 | <0.001 | 0.79 | 0.87 |
|  | B4 | 0.63 | 0.03 | 18.66 | <0.001 | 0.56 | 0.70 | 0.69 | 0.04 | 19.57 | <0.001 | 0.62 | 0.76 |
|  | B5 | 0.75 | 0.03 | 26.59 | <0.001 | 0.70 | 0.81 | 0.81 | 0.02 | 34.54 | <0.001 | 0.76 | 0.85 |
|  | B6 | 0.84 | 0.02 | 40.32 | <0.001 | 0.80 | 0.88 | 0.88 | 0.02 | 51.12 | <0.001 | 0.84 | 0.91 |
|  | B7 | 0.75 | 0.02 | 30.68 | <0.001 | 0.70 | 0.79 | 0.80 | 0.02 | 34.07 | <0.001 | 0.75 | 0.85 |
| Daily Life  and  Autonomy | C1 | 0.70 | 0.03 | 22.87 | <0.001 | 0.64 | 0.76 | 0.78 | 0.03 | 27.56 | <0.001 | 0.73 | 0.84 |
|  | C2 | 0.71 | 0.03 | 20.55 | <0.001 | 0.64 | 0.77 | 0.79 | 0.03 | 26.88 | <0.001 | 0.73 | 0.84 |
|  | C3 | 0.64 | 0.03 | 18.40 | <0.001 | 0.57 | 0.71 | 0.73 | 0.03 | 22.40 | <0.001 | 0.67 | 0.79 |
|  | C4 | 0.58 | 0.04 | 14.60 | <0.001 | 0.50 | 0.66 | 0.67 | 0.04 | 15.73 | <0.001 | 0.59 | 0.76 |
|  | C5 | 0.63 | 0.04 | 17.78 | <0.001 | 0.56 | 0.70 | 0.72 | 0.03 | 21.03 | <0.001 | 0.65 | 0.79 |
|  | C6 | 0.67 | 0.03 | 20.09 | <0.001 | 0.60 | 0.73 | 0.75 | 0.03 | 25.07 | <0.001 | 0.69 | 0.81 |
|  | C7 | 0.79 | 0.02 | 35.77 | <0.001 | 0.75 | 0.83 | 0.86 | 0.02 | 45.16 | <0.001 | 0.82 | 0.89 |
| Social Relationships | D1 | 0.71 | 0.03 | 22.05 | <0.001 | 0.65 | 0.77 | 0.78 | 0.03 | 25.07 | <0.001 | 0.72 | 0.84 |
|  | D2 | 0.66 | 0.04 | 17.65 | <0.001 | 0.59 | 0.74 | 0.74 | 0.03 | 21.23 | <0.001 | 0.67 | 0.81 |
|  | D3 | 0.72 | 0.03 | 21.03 | <0.001 | 0.65 | 0.78 | 0.79 | 0.03 | 26.36 | <0.001 | 0.73 | 0.85 |
|  | D4 | 0.61 | 0.04 | 16.98 | <0.001 | 0.54 | 0.68 | 0.69 | 0.04 | 17.68 | <0.001 | 0.61 | 0.77 |
|  | D5 | 0.61 | 0.04 | 16.64 | <0.001 | 0.53 | 0.68 | 0.69 | 0.04 | 16.93 | <0.001 | 0.61 | 0.77 |
|  | D6 | 0.69 | 0.04 | 18.13 | <0.001 | 0.61 | 0.76 | 0.76 | 0.03 | 24.58 | <0.001 | 0.70 | 0.82 |
| Emotional  Problems | E1 | 0.70 | 0.03 | 23.56 | <0.001 | 0.64 | 0.76 | 0.78 | 0.03 | 28.74 | <0.001 | 0.73 | 0.84 |
|  | E2 | 0.65 | 0.03 | 20.33 | <0.001 | 0.58 | 0.71 | 0.74 | 0.03 | 22.56 | <0.001 | 0.67 | 0.80 |
|  | E3 | 0.79 | 0.03 | 30.51 | <0.001 | 0.74 | 0.84 | 0.86 | 0.02 | 41.86 | <0.001 | 0.82 | 0.90 |
|  | E4 | 0.82 | 0.02 | 34.87 | <0.001 | 0.77 | 0.86 | 0.88 | 0.02 | 52.54 | <0.001 | 0.84 | 0.91 |
|  | E5 | 0.73 | 0.03 | 28.25 | <0.001 | 0.68 | 0.78 | 0.81 | 0.02 | 33.91 | <0.001 | 0.76 | 0.85 |
| Physical  Problems | F1 | 0.69 | 0.03 | 22.64 | <0.001 | 0.63 | 0.75 | 0.81 | 0.03 | 29.19 | <0.001 | 0.76 | 0.86 |
|  | F2 | 0.70 | 0.03 | 24.13 | <0.001 | 0.64 | 0.76 | 0.81 | 0.03 | 30.30 | <0.001 | 0.76 | 0.87 |
|  | F3 | 0.61 | 0.04 | 17.24 | <0.001 | 0.55 | 0.68 | 0.74 | 0.03 | 25.52 | <0.001 | 0.69 | 0.80 |
|  | F4 | 0.56 | 0.04 | 15.67 | <0.001 | 0.49 | 0.64 | 0.70 | 0.03 | 20.17 | <0.001 | 0.63 | 0.77 |
|  | F5 | 0.69 | 0.03 | 21.72 | <0.001 | 0.63 | 0.75 | 0.80 | 0.02 | 34.19 | <0.001 | 0.76 | 0.85 |

*Note*. Group: (1) general population sample, (2) TBI sample, Estimate: factor loading, *SE*: standard error, *z*: *z*-value, *p*: *p*-value, 95% CI: 95% confidence interval.

Table S23. Standardized Factor Variances for Measurement Invariance Model 4 (Strict Invariance) for QoLIBRI Scales (MLR Estimator)

|  |  |  | Group 1 | | | | | | Group 2 | | | | | |
| --- | --- | --- | --- | --- | --- | --- | --- | --- | --- | --- | --- | --- | --- | --- |
| Scale |  | Items | Estimate | *SE* | *z* | *p* | 95% CI lower | 95% CI upper | Estimate | *SE* | *z* | *p* | 95% CI lower | 95% CI upper |
| Cognition |  | A1 | 0.45 | 0.05 | 8.70 | <0.001 | 0.35 | 0.56 | 0.30 | 0.05 | 6.25 | <0.001 | 0.21 | 0.40 |
|  |  | A2 | 0.52 | 0.05 | 10.19 | <0.001 | 0.42 | 0.62 | 0.36 | 0.04 | 8.04 | <0.001 | 0.27 | 0.45 |
|  |  | A3 | 0.53 | 0.05 | 11.11 | <0.001 | 0.44 | 0.62 | 0.37 | 0.04 | 8.52 | <0.001 | 0.29 | 0.46 |
|  |  | A4 | 0.43 | 0.04 | 10.23 | <0.001 | 0.34 | 0.51 | 0.28 | 0.03 | 8.27 | <0.001 | 0.21 | 0.35 |
|  |  | A5 | 0.58 | 0.05 | 11.86 | <0.001 | 0.48 | 0.67 | 0.42 | 0.05 | 8.77 | <0.001 | 0.32 | 0.51 |
|  |  | A6 | 0.66 | 0.05 | 13.57 | <0.001 | 0.56 | 0.75 | 0.50 | 0.05 | 10.18 | <0.001 | 0.41 | 0.60 |
|  |  | A7 | 0.40 | 0.04 | 9.53 | <0.001 | 0.31 | 0.48 | 0.26 | 0.04 | 7.02 | <0.001 | 0.19 | 0.33 |
| Self |  | B1 | 0.52 | 0.04 | 11.94 | <0.001 | 0.43 | 0.60 | 0.43 | 0.05 | 8.98 | <0.001 | 0.34 | 0.53 |
|  |  | B2 | 0.51 | 0.04 | 11.26 | <0.001 | 0.42 | 0.59 | 0.42 | 0.04 | 10.09 | <0.001 | 0.34 | 0.50 |
|  |  | B3 | 0.38 | 0.03 | 11.51 | <0.001 | 0.32 | 0.45 | 0.31 | 0.03 | 9.34 | <0.001 | 0.24 | 0.37 |
|  |  | B4 | 0.60 | 0.04 | 14.23 | <0.001 | 0.52 | 0.69 | 0.52 | 0.05 | 10.62 | <0.001 | 0.42 | 0.62 |
|  |  | B5 | 0.43 | 0.04 | 10.07 | <0.001 | 0.35 | 0.51 | 0.35 | 0.04 | 9.30 | <0.001 | 0.28 | 0.42 |
|  |  | B6 | 0.30 | 0.03 | 8.58 | <0.001 | 0.23 | 0.37 | 0.23 | 0.03 | 7.74 | <0.001 | 0.17 | 0.29 |
|  |  | B7 | 0.44 | 0.04 | 12.14 | <0.001 | 0.37 | 0.51 | 0.36 | 0.04 | 9.59 | <0.001 | 0.29 | 0.43 |
| Daily Life  and  Autonomy |  | C1 | 0.51 | 0.04 | 12.00 | <0.001 | 0.43 | 0.60 | 0.39 | 0.04 | 8.82 | <0.001 | 0.30 | 0.48 |
|  |  | C2 | 0.50 | 0.05 | 10.36 | <0.001 | 0.41 | 0.60 | 0.38 | 0.05 | 8.26 | <0.001 | 0.29 | 0.47 |
|  |  | C3 | 0.59 | 0.04 | 13.16 | <0.001 | 0.50 | 0.68 | 0.47 | 0.05 | 9.77 | <0.001 | 0.37 | 0.56 |
|  |  | C4 | 0.67 | 0.05 | 14.52 | <0.001 | 0.58 | 0.76 | 0.55 | 0.06 | 9.53 | <0.001 | 0.44 | 0.66 |
|  |  | C5 | 0.60 | 0.04 | 13.39 | <0.001 | 0.51 | 0.69 | 0.48 | 0.05 | 9.65 | <0.001 | 0.38 | 0.58 |
|  |  | C6 | 0.56 | 0.04 | 12.65 | <0.001 | 0.47 | 0.64 | 0.43 | 0.05 | 9.63 | <0.001 | 0.35 | 0.52 |
|  |  | C7 | 0.37 | 0.04 | 10.68 | <0.001 | 0.31 | 0.44 | 0.27 | 0.03 | 8.22 | <0.001 | 0.20 | 0.33 |
| Social Relationships |  | D1 | 0.50 | 0.05 | 10.93 | <0.001 | 0.41 | 0.59 | 0.39 | 0.05 | 7.96 | <0.001 | 0.29 | 0.48 |
|  |  | D2 | 0.56 | 0.05 | 11.22 | <0.001 | 0.46 | 0.66 | 0.45 | 0.05 | 8.65 | <0.001 | 0.35 | 0.55 |
|  |  | D3 | 0.49 | 0.05 | 9.93 | <0.001 | 0.39 | 0.58 | 0.38 | 0.05 | 7.97 | <0.001 | 0.28 | 0.47 |
|  |  | D4 | 0.63 | 0.04 | 14.49 | <0.001 | 0.55 | 0.72 | 0.52 | 0.05 | 9.67 | <0.001 | 0.42 | 0.63 |
|  |  | D5 | 0.63 | 0.04 | 14.39 | <0.001 | 0.55 | 0.72 | 0.53 | 0.06 | 9.37 | <0.001 | 0.42 | 0.64 |
|  |  | D6 | 0.53 | 0.05 | 10.09 | <0.001 | 0.42 | 0.63 | 0.42 | 0.05 | 8.77 | <0.001 | 0.32 | 0.51 |
| Emotional Problems |  | E1 | 0.51 | 0.04 | 12.41 | <0.001 | 0.43 | 0.59 | 0.39 | 0.04 | 9.12 | <0.001 | 0.30 | 0.47 |
|  |  | E2 | 0.58 | 0.04 | 14.16 | <0.001 | 0.50 | 0.66 | 0.46 | 0.05 | 9.46 | <0.001 | 0.36 | 0.55 |
|  |  | E3 | 0.37 | 0.04 | 8.97 | <0.001 | 0.29 | 0.45 | 0.26 | 0.04 | 7.41 | <0.001 | 0.19 | 0.33 |
|  |  | E4 | 0.33 | 0.04 | 8.73 | <0.001 | 0.26 | 0.41 | 0.23 | 0.03 | 7.92 | <0.001 | 0.17 | 0.29 |
|  |  | E5 | 0.47 | 0.04 | 12.69 | <0.001 | 0.40 | 0.55 | 0.35 | 0.04 | 9.18 | <0.001 | 0.28 | 0.43 |
| Physical Problems |  | F1 | 0.52 | 0.04 | 12.21 | <0.001 | 0.44 | 0.60 | 0.34 | 0.04 | 7.68 | <0.001 | 0.26 | 0.43 |
|  |  | F2 | 0.51 | 0.04 | 12.57 | <0.001 | 0.43 | 0.59 | 0.34 | 0.04 | 7.70 | <0.001 | 0.25 | 0.42 |
|  |  | F3 | 0.62 | 0.04 | 14.17 | <0.001 | 0.54 | 0.71 | 0.45 | 0.04 | 10.23 | <0.001 | 0.36 | 0.53 |
|  |  | F4 | 0.68 | 0.04 | 16.72 | <0.001 | 0.60 | 0.76 | 0.51 | 0.05 | 10.49 | <0.001 | 0.41 | 0.61 |
|  |  | F5 | 0.53 | 0.04 | 12.11 | <0.001 | 0.44 | 0.61 | 0.35 | 0.04 | 9.30 | <0.001 | 0.28 | 0.43 |

*Note*. Group: (1) general population sample, (2) TBI sample, Estimate: variance, *SE*: standard error, *z*: *z*-value, *p*: *p*-value, 95% CI: 95% confidence interval.

Table S24. Standardized Factor Covariances for Measurement Invariance Model 4 (Strict Invariance) for QoLIBRI Scales (MLR Estimator)

| Scale 1 | Scale 2 | Group | Estimate | *SE* | *z* | *p* | 95% CI lower | 95% CI upper |
| --- | --- | --- | --- | --- | --- | --- | --- | --- |
| Cognition | Self | 1 | 0.69 | 0.05 | 15.26 | <0.001 | 0.60 | 0.78 |
|  | Daily Life and Autonomy | 1 | 0.68 | 0.05 | 14.05 | <0.001 | 0.58 | 0.77 |
|  | Social Relationships | 1 | 0.53 | 0.06 | 8.44 | <0.001 | 0.41 | 0.65 |
|  | Emotional Problems | 1 | 0.45 | 0.06 | 7.91 | <0.001 | 0.34 | 0.56 |
|  | Physical Problems | 1 | 0.39 | 0.07 | 5.75 | <0.001 | 0.26 | 0.52 |
| Self | Daily Life and Autonomy | 1 | 0.80 | 0.04 | 20.40 | <0.001 | 0.72 | 0.87 |
|  | Social Relationships | 1 | 0.69 | 0.05 | 14.34 | <0.001 | 0.60 | 0.79 |
|  | Emotional Problems | 1 | 0.41 | 0.07 | 6.30 | <0.001 | 0.28 | 0.54 |
|  | Physical Problems | 1 | 0.27 | 0.07 | 3.83 | <0.001 | 0.13 | 0.41 |
| Daily Life and  Autonomy | Social Relationships | 1 | 0.78 | 0.04 | 19.97 | <0.001 | 0.70 | 0.85 |
|  | Emotional Problems | 1 | 0.46 | 0.06 | 7.47 | <0.001 | 0.34 | 0.58 |
|  | Physical Problems | 1 | 0.38 | 0.07 | 5.47 | <0.001 | 0.24 | 0.51 |
| Social Relationships | Emotional Problems | 1 | 0.33 | 0.07 | 4.67 | <0.001 | 0.19 | 0.46 |
|  | Physical Problems | 1 | 0.19 | 0.07 | 2.62 | 0.009 | 0.05 | 0.33 |
| Emotional Problems | Physical Problems | 1 | 0.72 | 0.04 | 16.84 | <0.001 | 0.63 | 0.80 |
| Cognition | Self | 2 | 0.83 | 0.04 | 21.65 | <0.001 | 0.76 | 0.91 |
|  | Daily Life and Autonomy | 2 | 0.83 | 0.04 | 20.81 | <0.001 | 0.75 | 0.90 |
|  | Social Relationships | 2 | 0.63 | 0.07 | 8.83 | <0.001 | 0.49 | 0.78 |
|  | Emotional Problems | 2 | -0.29 | 0.09 | -3.16 | 0.002 | -0.47 | -0.11 |
|  | Physical Problems | 2 | -0.10 | 0.10 | -0.99 | 0.322 | -0.29 | 0.10 |
| Self | Daily Life and Autonomy | 2 | 0.87 | 0.03 | 31.46 | <0.001 | 0.82 | 0.93 |
|  | Social Relationships | 2 | 0.86 | 0.03 | 26.49 | <0.001 | 0.79 | 0.92 |
|  | Emotional Problems | 2 | -0.22 | 0.10 | -2.22 | 0.027 | -0.41 | -0.03 |
|  | Physical Problems | 2 | -0.15 | 0.10 | -1.50 | 0.133 | -0.35 | 0.05 |
| Daily Life and  Autonomy | Social Relationships | 2 | 0.68 | 0.07 | 10.20 | <0.001 | 0.55 | 0.81 |
|  | Emotional Problems | 2 | -0.20 | 0.09 | -2.14 | 0.032 | -0.39 | -0.02 |
|  | Physical Problems | 2 | -0.10 | 0.10 | -1.00 | 0.315 | -0.29 | 0.09 |
| Social Relationships | Emotional Problems | 2 | -0.19 | 0.10 | -1.98 | 0.048 | -0.38 | 0.00 |
|  | Physical Problems | 2 | -0.25 | 0.09 | -2.69 | 0.007 | -0.43 | -0.07 |
| Emotional Problems | Physical Problems | 2 | 0.63 | 0.07 | 8.47 | <0.001 | 0.49 | 0.78 |

*Note*. Group: (1) general population sample, (2) TBI sample, Estimate: covariance, *SE*: standard error, *z*: *z*-value, *p*: *p*-value, 95% CI: 95% confidence interval.

## QoLIBRI–OS – MLR

### Model 1

Table S25. Standardized Factor Loadings for Measurement Invariance Model 1 (Configural Invariance) for QoLIBRI-OS (MLR Estimator)

|  |  | Group 1 | | | | | | Group 2 | | | | | |
| --- | --- | --- | --- | --- | --- | --- | --- | --- | --- | --- | --- | --- | --- |
| Scale | Items | Estimate | *SE* | *z* | *p* | 95% CI  lower | 95% CI  upper | Estimate | *SE* | *z* | *p* | 95% CI  lower | 95% CI  upper |
| QoLIBRI-OS | O1 | 0.66 | 0.04 | 16.29 | <0.001 | 0.58 | 0.74 | 0.78 | 0.03 | 23.49 | <0.001 | 0.71 | 0.84 |
|  | O2 | 0.66 | 0.05 | 13.65 | <0.001 | 0.57 | 0.76 | 0.70 | 0.04 | 18.97 | <0.001 | 0.63 | 0.77 |
|  | O3 | 0.83 | 0.02 | 36.67 | <0.001 | 0.78 | 0.87 | 0.77 | 0.04 | 21.47 | <0.001 | 0.70 | 0.84 |
|  | O4 | 0.77 | 0.03 | 25.29 | <0.001 | 0.71 | 0.82 | 0.79 | 0.03 | 26.71 | <0.001 | 0.73 | 0.84 |
|  | O5 | 0.71 | 0.04 | 18.78 | <0.001 | 0.64 | 0.79 | 0.77 | 0.04 | 21.50 | <0.001 | 0.70 | 0.84 |
|  | O6 | 0.74 | 0.04 | 20.01 | <0.001 | 0.66 | 0.81 | 0.80 | 0.03 | 27.61 | <0.001 | 0.74 | 0.86 |

*Note*. Group: (1) general population sample, (2) TBI sample, Estimate: factor loading, *SE*: standard error, *z*: *z*-value, *p*: *p*-value, 95% CI: 95% confidence interval.

Table S26. Standardized Factor Variances for Measurement Invariance Model 1 (Configural Invariance) for QoLIBRI-OS (MLR Estimator)

|  |  | Group 1 | | | | | | Group 2 | | | | | |
| --- | --- | --- | --- | --- | --- | --- | --- | --- | --- | --- | --- | --- | --- |
| Scale | Items | Estimate | *SE* | *z* | *p* | 95% CI  lower | 95% CI  upper | Estimate | *SE* | *z* | *p* | 95% CI  lower | 95% CI  upper |
| QoLIBRI-OS | O1 | 0.57 | 0.05 | 10.71 | <0.001 | 0.46 | 0.67 | 0.39 | 0.05 | 7.63 | <0.001 | 0.29 | 0.49 |
|  | O2 | 0.56 | 0.06 | 8.68 | <0.001 | 0.43 | 0.69 | 0.51 | 0.05 | 10.00 | <0.001 | 0.41 | 0.61 |
|  | O3 | 0.32 | 0.04 | 8.55 | <0.001 | 0.25 | 0.39 | 0.40 | 0.06 | 7.23 | <0.001 | 0.29 | 0.51 |
|  | O4 | 0.41 | 0.05 | 8.95 | <0.001 | 0.32 | 0.51 | 0.38 | 0.05 | 8.28 | <0.001 | 0.29 | 0.47 |
|  | O5 | 0.49 | 0.05 | 9.18 | <0.001 | 0.39 | 0.60 | 0.41 | 0.05 | 7.48 | <0.001 | 0.30 | 0.52 |
|  | O6 | 0.46 | 0.05 | 8.43 | <0.001 | 0.35 | 0.56 | 0.36 | 0.05 | 7.81 | <0.001 | 0.27 | 0.45 |

*Note*. Group: (1) general population sample, (2) TBI sample, Estimate: variance, *SE*: standard error, *z*: *z*-value, *p*: *p*-value, 95% CI: 95% confidence interval.

### Model 2

Table S27. Standardized Factor Loadings for Measurement Invariance Model 2 (Metric Invariance) for QoLIBRI-OS (MLR Estimator)

|  |  | Group 1 | | | | | | Group 2 | | | | | |
| --- | --- | --- | --- | --- | --- | --- | --- | --- | --- | --- | --- | --- | --- |
| Scale | Items | Estimate | *SE* | *z* | *p* | 95% CI  lower | 95% CI  upper | Estimate | *SE* | *z* | *p* | 95% CI  lower | 95% CI  upper |
| QoLIBRI-OS | O1 | 0.71 | 0.03 | 24.64 | <0.001 | 0.65 | 0.76 | 0.73 | 0.04 | 20.66 | <0.001 | 0.66 | 0.80 |
|  | O2 | 0.65 | 0.04 | 17.48 | <0.001 | 0.57 | 0.72 | 0.71 | 0.03 | 21.81 | <0.001 | 0.64 | 0.77 |
|  | O3 | 0.82 | 0.02 | 37.79 | <0.001 | 0.78 | 0.86 | 0.78 | 0.03 | 27.08 | <0.001 | 0.73 | 0.84 |
|  | O4 | 0.73 | 0.03 | 24.23 | <0.001 | 0.67 | 0.79 | 0.81 | 0.02 | 34.90 | <0.001 | 0.76 | 0.85 |
|  | O5 | 0.72 | 0.03 | 23.76 | <0.001 | 0.66 | 0.78 | 0.77 | 0.03 | 24.30 | <0.001 | 0.70 | 0.83 |
|  | O6 | 0.74 | 0.03 | 24.96 | <0.001 | 0.69 | 0.80 | 0.79 | 0.03 | 28.79 | <0.001 | 0.74 | 0.85 |

*Note*. Group: (1) general population sample, (2) TBI sample, Estimate: factor loading, *SE*: standard error, *z*: *z*-value, *p*: *p*-value, 95% CI: 95% confidence interval.

Table S28. Standardized Factor Variances for Measurement Invariance Model 2 (Metric Invariance) for QoLIBRI-OS (MLR Estimator)

|  |  | Group 1 | | | | | | Group 2 | | | | | |
| --- | --- | --- | --- | --- | --- | --- | --- | --- | --- | --- | --- | --- | --- |
| Scale | Items | Estimate | *SE* | *z* | *p* | 95% CI  lower | 95% CI  upper | Estimate | *SE* | *z* | *p* | 95% CI  lower | 95% CI  upper |
| QoLIBRI-OS | O1 | 0.50 | 0.04 | 12.45 | <0.001 | 0.42 | 0.58 | 0.47 | 0.05 | 9.04 | <0.001 | 0.37 | 0.57 |
|  | O2 | 0.58 | 0.05 | 12.13 | <0.001 | 0.49 | 0.68 | 0.50 | 0.05 | 10.98 | <0.001 | 0.41 | 0.59 |
|  | O3 | 0.33 | 0.04 | 9.20 | <0.001 | 0.26 | 0.40 | 0.38 | 0.05 | 8.46 | <0.001 | 0.30 | 0.47 |
|  | O4 | 0.47 | 0.04 | 10.62 | <0.001 | 0.38 | 0.55 | 0.35 | 0.04 | 9.41 | <0.001 | 0.28 | 0.42 |
|  | O5 | 0.48 | 0.04 | 11.05 | <0.001 | 0.40 | 0.57 | 0.41 | 0.05 | 8.53 | <0.001 | 0.32 | 0.51 |
|  | O6 | 0.45 | 0.04 | 10.02 | <0.001 | 0.36 | 0.53 | 0.37 | 0.04 | 8.49 | <0.001 | 0.29 | 0.46 |

*Note*. Group: (1) general population sample, (2) TBI sample, Estimate: variance, *SE*: standard error, *z*: *z*-value, *p*: *p*-value, 95% CI: 95% confidence interval.

### Model 3

Table S29. Standardized Factor Loadings for Measurement Invariance Model 3 (Scalar Invariance) for QoLIBRI-OS (MLR Estimator)

|  |  | Group 1 | | | | | | Group 2 | | | | | |  |
| --- | --- | --- | --- | --- | --- | --- | --- | --- | --- | --- | --- | --- | --- | --- |
| Scale | Items | Estimate | *SE* | *z* | *p* | 95% CI  lower | 95% CI  upper | Estimate | *SE* | *z* | *p* | 95% CI  lower | 95% CI  upper |  |
| QoLIBRI-OS | O1 | 0.70 | 0.03 | 23.78 | <0.001 | 0.64 | 0.76 | 0.72 | 0.04 | 19.91 | <0.001 | 0.65 | 0.79 |  |
|  | O2 | 0.63 | 0.04 | 16.61 | <0.001 | 0.56 | 0.71 | 0.69 | 0.03 | 20.26 | <0.001 | 0.62 | 0.76 |  |
|  | O3 | 0.82 | 0.02 | 38.21 | <0.001 | 0.78 | 0.86 | 0.79 | 0.03 | 27.03 | <0.001 | 0.73 | 0.84 |  |
|  | O4 | 0.73 | 0.03 | 24.12 | <0.001 | 0.67 | 0.79 | 0.80 | 0.02 | 34.64 | <0.001 | 0.76 | 0.85 |  |
|  | O5 | 0.72 | 0.03 | 24.05 | <0.001 | 0.66 | 0.78 | 0.77 | 0.03 | 24.64 | <0.001 | 0.71 | 0.83 |  |
|  | O6 | 0.75 | 0.03 | 25.04 | <0.001 | 0.69 | 0.80 | 0.79 | 0.03 | 28.81 | <0.001 | 0.74 | 0.85 |  |

*Note*. Group: (1) general population sample, (2) TBI sample, Estimate: factor loading, *SE*: standard error, *z*: *z*-value, *p*: *p*-value, 95% CI: 95% confidence interval.

Table S30. Standardized Factor Variances for Measurement Invariance Model 3 (Scalar Invariance) for QoLIBRI-OS (MLR Estimator)

|  |  | Group 1 | | | | | | Group 2 | | | | | |
| --- | --- | --- | --- | --- | --- | --- | --- | --- | --- | --- | --- | --- | --- |
| Scale | Items | Estimate | *SE* | *z* | *p* | 95% CI  lower | 95% CI  upper | Estimate | *SE* | *z* | *p* | 95% CI  lower | 95% CI  upper |
| QoLIBRI-OS | O1 | 0.51 | 0.04 | 12.45 | <0.001 | 0.43 | 0.59 | 0.48 | 0.05 | 9.31 | <0.001 | 0.38 | 0.59 |
|  | O2 | 0.60 | 0.05 | 12.32 | <0.001 | 0.50 | 0.69 | 0.52 | 0.05 | 11.10 | <0.001 | 0.43 | 0.62 |
|  | O3 | 0.33 | 0.04 | 9.21 | <0.001 | 0.26 | 0.39 | 0.38 | 0.05 | 8.36 | <0.001 | 0.29 | 0.47 |
|  | O4 | 0.47 | 0.04 | 10.67 | <0.001 | 0.38 | 0.56 | 0.35 | 0.04 | 9.43 | <0.001 | 0.28 | 0.43 |
|  | O5 | 0.48 | 0.04 | 11.03 | <0.001 | 0.39 | 0.56 | 0.41 | 0.05 | 8.48 | <0.001 | 0.31 | 0.50 |
|  | O6 | 0.44 | 0.04 | 9.99 | <0.001 | 0.36 | 0.53 | 0.37 | 0.04 | 8.47 | <0.001 | 0.28 | 0.46 |

*Note*. Group: (1) general population sample, (2) TBI sample, Estimate: variance, *SE*: standard error, *z*: *z*-value, *p*: *p*-value, 95% CI: 95% confidence interval.

### Model 4

Table S31. Standardized Factor Loadings for Measurement Invariance Model 4 (Strict Invariance) for QoLIBRI-OS (MLR Estimator)

|  |  | Group 1 | | | | | | Group 2 | | | | | |
| --- | --- | --- | --- | --- | --- | --- | --- | --- | --- | --- | --- | --- | --- |
| Scale | Items | Estimate | *SE* | *z* | *p* | 95% CI  lower | 95% CI  upper | Estimate | *SE* | *z* | *p* | 95% CI  lower | 95% CI  upper |
| QoLIBRI-OS | O1 | 0.69 | 0.03 | 24.27 | <0.001 | 0.64 | 0.75 | 0.73 | 0.03 | 24.56 | <0.001 | 0.67 | 0.78 |
|  | O2 | 0.65 | 0.03 | 19.00 | <0.001 | 0.58 | 0.71 | 0.68 | 0.03 | 19.81 | <0.001 | 0.61 | 0.75 |
|  | O3 | 0.79 | 0.02 | 34.60 | <0.001 | 0.74 | 0.83 | 0.82 | 0.02 | 36.92 | <0.001 | 0.77 | 0.86 |
|  | O4 | 0.75 | 0.03 | 29.38 | <0.001 | 0.70 | 0.80 | 0.78 | 0.02 | 36.51 | <0.001 | 0.74 | 0.82 |
|  | O5 | 0.73 | 0.03 | 26.38 | <0.001 | 0.68 | 0.78 | 0.76 | 0.03 | 28.68 | <0.001 | 0.71 | 0.81 |
|  | O6 | 0.75 | 0.03 | 28.78 | <0.001 | 0.70 | 0.80 | 0.78 | 0.02 | 31.60 | <0.001 | 0.73 | 0.83 |

*Note*. Group: (1) general population sample, (2) TBI sample, Estimate: factor loading, *SE*: standard error, *z*: *z*-value, *p*: *p*-value, 95% CI: 95% confidence interval.

Table S32. Standardized Factor Variances for Measurement Invariance Model 4 (Strict Invariance) for QoLIBRI-OS (MLR Estimator)

|  |  | Group 1 | | | | | | Group 2 | | | | | |
| --- | --- | --- | --- | --- | --- | --- | --- | --- | --- | --- | --- | --- | --- |
| Scale | Items | Estimate | *SE* | *z* | *p* | 95% CI  lower | 95% CI  upper | Estimate | *SE* | *z* | *p* | 95% CI  lower | 95% CI  upper |
| QoLIBRI-OS | O1 | 0.52 | 0.04 | 13.19 | <0.001 | 0.44 | 0.60 | 0.47 | 0.04 | 11.00 | <0.001 | 0.39 | 0.56 |
|  | O2 | 0.58 | 0.04 | 13.32 | <0.001 | 0.50 | 0.67 | 0.54 | 0.05 | 11.45 | <0.001 | 0.44 | 0.63 |
|  | O3 | 0.38 | 0.04 | 10.46 | <0.001 | 0.31 | 0.45 | 0.33 | 0.04 | 9.20 | <0.001 | 0.26 | 0.40 |
|  | O4 | 0.43 | 0.04 | 11.26 | <0.001 | 0.36 | 0.51 | 0.39 | 0.03 | 11.53 | <0.001 | 0.32 | 0.45 |
|  | O5 | 0.47 | 0.04 | 11.55 | <0.001 | 0.39 | 0.55 | 0.42 | 0.04 | 10.34 | <0.001 | 0.34 | 0.50 |
|  | O6 | 0.43 | 0.04 | 11.04 | <0.001 | 0.36 | 0.51 | 0.39 | 0.04 | 9.98 | <0.001 | 0.31 | 0.46 |

*Note*. Group: (1) general population sample, (2) TBI sample, Estimate: variance, *SE*: standard error, *z*: *z*-value, *p*: *p*-value, 95% CI: 95% confidence interval.

# Regression Analysis

## Overview on Regression Analyses Results

Table S33. Summary on Measurement Invariance Analyses Results

| Scale |  | *b* | *SE* | *t* | *p* |
| --- | --- | --- | --- | --- | --- |
| QoLIBRI | Intercept | 65.17 | 0.69 | 95 | <0.001 |
|  | Sex (Female) | -0.66 | 0.49 | -1.36 | 0.175 |
|  | Age (Medium-aged Adults) | 7.37 | 0.56 | 13.09 | <0.001 |
|  | Age (Older Adults) | 14.88 | 0.66 | 22.51 | <0.001 |
|  | Edu (Low Education) | -6.16 | 0.97 | -6.36 | <0.001 |
|  | Edu (Medium Education) | -2.05 | 0.58 | -3.54 | <0.001 |
|  | Chronic Health Condition (Yes) | -6.34 | 0.49 | -12.88 | <0.001 |
| QoLIBRI-OS | Intercept | 65.8 | 0.89 | 74.07 | <0.001 |
|  | Sex (Female) | 0.22 | 0.63 | 0.35 | 0.727 |
|  | Age (Medium-aged Adults) | 1.86 | 0.73 | 2.56 | 0.011 |
|  | Age (Older Adults) | 8.07 | 0.86 | 9.43 | <0.001 |
|  | Education (Low Education) | -7.98 | 1.25 | -6.36 | <0.001 |
|  | Education (Medium Education) | -3.11 | 0.75 | -4.15 | <0.001 |
|  | Chronic Health Condition (Yes) | -7.94 | 0.64 | -12.47 | <0.001 |

*Note*. Reference groups: Sex (Male), Age (Young adults), Education (High Education), Chronic Health Condition (No); *b*: non-standardized regression coefficient, *SE*: standard error, t*:* *t*-value, *p*: *p*-value.

## QoLIBRI(-OS) Scales and Second–Order Regression Analyses

### Cognition

Table S34. Results of Regression-Analysis for the QoLIBRI Cognition Scale

|  | *b* | *SE* | *t* | *p* |
| --- | --- | --- | --- | --- |
| Intercept | 69.72 | 0.82 | 84.55 | <0.001 |
| Sex (Female) | -0.77 | 0.59 | -1.31 | 0.189 |
| Age (Medium-aged Adults) | 8.93 | 0.68 | 13.21 | <0.001 |
| Age (Older Adults) | 15.63 | 0.79 | 19.67 | <0.001 |
| Education (Low Education) | -9.55 | 1.16 | -8.2 | <0.001 |
| Education (Medium Education) | -3.55 | 0.7 | -5.1 | <0.001 |
| Chronic Health Condition (Yes) | -3.59 | 0.59 | -6.07 | <0.001 |

*Note*. Reference groups: Sex (Male), Age (Young adults), Education (High Education), Chronic Health Condition (No); *b*: non-standardized regression coefficient, *SE*: standard error, t*:* *t*-value, *p*: *p*-value.

Table S35. Results of Second-Order Regression Model for the QoLIBRI Cognition Scale

|  | *b* | *SE* | *t* | *p* |
| --- | --- | --- | --- | --- |
| Intercept | 72.34 | 1.57 | 46.13 | <0.001 |
| Sex (Female) | -2.56 | 1.58 | -1.62 | 0.106 |
| Age (Medium-aged Adults) | 7.57 | 1.73 | 4.39 | <0.001 |
| Age (Older Adults) | 12.61 | 1.95 | 6.46 | <0.001 |
| Education (Low Education) | -14.03 | 2.71 | -5.18 | <0.001 |
| Education (Medium Education) | -5.45 | 1.64 | -3.32 | 0.001 |
| Chronic Health Condition (Yes) | -5.65 | 1.66 | -3.40 | 0.001 |
| Sex (Female): Age (Medium-aged Adults) | -0.01 | 1.38 | 0.00 | 0.997 |
| Sex (Female): Age (Older Adults) | 1.02 | 1.62 | 0.63 | 0.527 |
| Sex (Female): Education (Low Education) | 3.30 | 2.38 | 1.39 | 0.165 |
| Sex (Female): Education (Medium Education) | -0.11 | 1.45 | -0.08 | 0.938 |
| Sex (Female): Chronic Health Condition (Yes) | 2.35 | 1.21 | 1.95 | 0.052 |
| Age (Medium-aged Adults): Education (Low Education) | 3.76 | 2.74 | 1.37 | 0.170 |
| Age (Older Adults): Education (Low Education) | 3.37 | 3.31 | 1.02 | 0.308 |
| Age (Medium-aged Adults): Education (Medium Education) | 1.19 | 1.71 | 0.70 | 0.486 |
| Age (Older Adults): Education (Medium Education) | 3.22 | 1.89 | 1.70 | 0.089 |
| Age (Medium-aged Adults): Chronic Health Condition (Yes) | 0.16 | 1.37 | 0.12 | 0.908 |
| Age (Older Adults): Chronic Health Condition (Yes) | 0.27 | 1.65 | 0.16 | 0.870 |
| Education (Low Education): Chronic Health Condition (Yes) | 0.30 | 2.41 | 0.12 | 0.901 |
| Education (Medium Education): Chronic Health Condition (Yes) | 1.05 | 1.44 | 0.73 | 0.465 |

*Note*. Reference groups: Sex (Male), Age (Young adults), Education (High Education), Chronic Health Condition (No); *b*: non-standardized regression coefficient, *SE*: standard error, t*:* *t*-value, *p*: *p*-value.

### Self

Table S36. Results of Regression Analysis for the QoLIBRI Self Scale

|  | *b* | *SE* | *t* | *p* |
| --- | --- | --- | --- | --- |
| Intercept | 61.88 | 0.93 | 66.62 | <0.001 |
| Sex (Female) | -2.60 | 0.66 | -3.94 | <0.001 |
| Age (Medium-aged Adults) | 3.12 | 0.76 | 4.10 | <0.001 |
| Age (Older Adults) | 11.12 | 0.89 | 12.42 | <0.001 |
| Education (Low Education) | -5.27 | 1.31 | -4.02 | <0.001 |
| Education (Medium Education) | -1.91 | 0.78 | -2.43 | 0.015 |
| Chronic Health Condition (Yes) | -6.64 | 0.67 | -9.97 | <0.001 |

*Note*. Reference groups: Sex (Male), Age (Young adults), Education (High Education), Chronic Health Condition (No); *b*: non-standardized regression coefficient, *SE*: standard error, t*:* *t*-value, *p*: *p*-value.

Table S37. Results of Second-Order Regression Model for the QoLIBRI Self Scale

|  | *b* | *SE* | *t* | *p* |
| --- | --- | --- | --- | --- |
| Intercept | 64.09 | 1.76 | 36.31 | <0.001 |
| Sex (Female) | -4.35 | 1.78 | -2.44 | 0.015 |
| Age (Medium-aged Adults) | 2.72 | 1.94 | 1.40 | 0.162 |
| Age (Older Adults) | 7.63 | 2.20 | 3.47 | 0.001 |
| Education (Low Education) | -11.30 | 3.05 | -3.70 | <0.001 |
| Education (Medium Education) | -3.42 | 1.85 | -1.85 | 0.064 |
| Chronic Health Condition (Yes) | -7.09 | 1.87 | -3.79 | <0.001 |
| Sex (Female): Age (Medium-aged Adults) | 2.21 | 1.55 | 1.43 | 0.154 |
| Sex (Female): Age (Older Adults) | 2.91 | 1.82 | 1.60 | 0.110 |
| Sex (Female): Education (Low Education) | 3.98 | 2.68 | 1.49 | 0.137 |
| Sex (Female): Education (Medium Education) | -0.80 | 1.63 | -0.49 | 0.622 |
| Sex (Female): Chronic Health Condition (Yes) | 0.43 | 1.36 | 0.32 | 0.749 |
| Age (Medium-aged Adults): Education (Low Education) | 3.91 | 3.08 | 1.27 | 0.204 |
| Age (Older Adults): Education (Low Education) | 5.47 | 3.72 | 1.47 | 0.142 |
| Age (Medium-aged Adults): Education (Medium Education) | -0.50 | 1.93 | -0.26 | 0.794 |
| Age (Older Adults): Education (Medium Education) | 3.52 | 2.13 | 1.65 | 0.098 |
| Age (Medium-aged Adults): Chronic Health Condition (Yes) | -1.99 | 1.54 | -1.29 | 0.196 |
| Age (Older Adults): Chronic Health Condition (Yes) | -1.50 | 1.85 | -0.81 | 0.417 |
| Education (Low Education): Chronic Health Condition (Yes) | 1.29 | 2.72 | 0.48 | 0.634 |
| Education (Medium Education): Chronic Health Condition (Yes) | 2.01 | 1.62 | 1.25 | 0.213 |

*Note*. Reference groups: Sex (Male), Age (Young adults), Education (High Education), Chronic Health Condition (No); *b*: non-standardized regression coefficient, *SE*: standard error, t*:* *t*-value, *p*: *p*-value.

### Daily Life and Autonomy

Table S38. Results of Regression Analysis for the QoLIBRI Daily Life and Autonomy Scale

|  | *b* | *SE* | *t* | *p* |
| --- | --- | --- | --- | --- |
| Intercept | 67.19 | 0.92 | 73.25 | <0.001 |
| Sex (Female) | -0.34 | 0.65 | -0.52 | 0.602 |
| Age (Medium-aged Adults) | 5.61 | 0.75 | 7.45 | <0.001 |
| Age (Older Adults) | 12.00 | 0.88 | 13.58 | <0.001 |
| Education (Low Education) | -8.51 | 1.30 | -6.57 | <0.001 |
| Education (Medium Education) | -2.20 | 0.77 | -2.84 | 0.005 |
| Chronic Health Condition (Yes) | -6.07 | 0.66 | -9.23 | <0.001 |

*Note*. Reference groups: Sex (Male), Age (Young adults), Education (High Education), Chronic Health Condition (No); *b*: non-standardized regression coefficient, *SE*: standard error, t*:* *t*-value, *p*: *p*-value.

Table S39. Results of Second-Order Regression Model for the QoLIBRI Autonomy and Daily Life Scale

|  | *b* | *SE* | *t* | *p* |
| --- | --- | --- | --- | --- |
| Intercept | 68.72 | 1.74 | 39.44 | <0.001 |
| Sex (Female) | -3.03 | 1.76 | -1.72 | 0.085 |
| Age (Medium-aged Adults) | 5.72 | 1.92 | 2.98 | 0.003 |
| Age (Older Adults) | 12.39 | 2.17 | 5.71 | <0.001 |
| Education (Low Education) | -15.35 | 3.01 | -5.10 | <0.001 |
| Education (Medium Education) | -2.84 | 1.82 | -1.56 | 0.120 |
| Chronic Health Condition (Yes) | -6.76 | 1.85 | -3.66 | <0.001 |
| Sex (Female): Age (Medium-aged Adults) | 1.79 | 1.53 | 1.17 | 0.241 |
| Sex (Female): Age (Older Adults) | -0.13 | 1.80 | -0.07 | 0.941 |
| Sex (Female): Education (Low Education) | 4.94 | 2.64 | 1.87 | 0.062 |
| Sex (Female): Education (Medium Education) | 0.24 | 1.61 | 0.15 | 0.882 |
| Sex (Female): Chronic Health Condition (Yes) | 2.45 | 1.34 | 1.82 | 0.069 |
| Age (Medium-aged Adults): Education (Low Education) | 4.88 | 3.04 | 1.60 | 0.109 |
| Age (Older Adults): Education (Low Education) | 2.80 | 3.68 | 0.76 | 0.447 |
| Age (Medium-aged Adults): Education (Medium Education) | -1.05 | 1.90 | -0.55 | 0.581 |
| Age (Older Adults): Education (Medium Education) | 1.47 | 2.10 | 0.70 | 0.483 |
| Age (Medium-aged Adults): Chronic Health Condition (Yes) | -1.94 | 1.52 | -1.27 | 0.203 |
| Age (Older Adults): Chronic Health Condition (Yes) | -2.99 | 1.83 | -1.63 | 0.102 |
| Education (Low Education): Chronic Health Condition (Yes) | 2.25 | 2.68 | 0.84 | 0.402 |
| Education (Medium Education): Chronic Health Condition (Yes) | 1.12 | 1.60 | 0.70 | 0.484 |

*Note*. Reference groups: Sex (Male), Age (Young adults), Education (High Education), Chronic Health Condition (No); *b*: non-standardized regression coefficient, *SE*: standard error, t*:* *t*-value, *p*: *p*-value.

### Social Relationships

Table S40. Results of Regression Analysis for the QoLIBRI Social Relationships Scale

|  | *b* | *SE* | *t* | *p* |
| --- | --- | --- | --- | --- |
| Intercept | 63.60 | 0.95 | 66.84 | <0.001 |
| Sex (Female) | 3.22 | 0.68 | 4.75 | <0.001 |
| Age (Medium-aged Adults) | 1.17 | 0.78 | 1.50 | 0.135 |
| Age (Older Adults) | 8.17 | 0.92 | 8.90 | <0.001 |
| Education (Low Education) | -3.78 | 1.34 | -2.81 | 0.005 |
| Education (Medium Education) | -0.78 | 0.80 | -0.98 | 0.328 |
| Chronic Health Condition (Yes) | -3.44 | 0.68 | -5.04 | <0.001 |

*Note*. Reference groups: Sex (Male), Age (Young adults), Education (High Education), Chronic Health Condition (No); *b*: non-standardized regression coefficient, *SE*: standard error, t*:* *t*-value, *p*: *p*-value.

Table S41. Results of Second-Order Regression Model for the QOLIBRI Social Relationships Scale

|  | *b* | *SE* | *t* | *p* |
| --- | --- | --- | --- | --- |
| Intercept | 64.69 | 1.81 | 35.75 | <0.001 |
| Sex (Female) | 3.53 | 1.83 | 1.93 | 0.054 |
| Age (Medium-aged Adults) | 1.06 | 1.99 | 0.53 | 0.593 |
| Age (Older Adults) | 6.84 | 2.25 | 3.04 | 0.002 |
| Education (Low Education) | -11.07 | 3.13 | -3.54 | <0.001 |
| Education (Medium Education) | -1.88 | 1.89 | -0.99 | 0.320 |
| Chronic Health Condition (Yes) | -3.99 | 1.92 | -2.08 | 0.037 |
| Sex (Female): Age (Medium-aged Adults) | -0.50 | 1.59 | -0.31 | 0.753 |
| Sex (Female): Age (Older Adults) | -1.91 | 1.86 | -1.03 | 0.305 |
| Sex (Female): Education (Low Education) | 3.10 | 2.74 | 1.13 | 0.258 |
| Sex (Female): Education (Medium Education) | -1.55 | 1.67 | -0.93 | 0.352 |
| Sex (Female): Chronic Health Condition (Yes) | 2.03 | 1.39 | 1.46 | 0.145 |
| Age (Medium-aged Adults): Education (Low Education) | 5.46 | 3.16 | 1.73 | 0.084 |
| Age (Older Adults): Education (Low Education) | 7.98 | 3.82 | 2.09 | 0.037 |
| Age (Medium-aged Adults): Education (Medium Education) | 1.16 | 1.98 | 0.58 | 0.559 |
| Age (Older Adults): Education (Medium Education) | 3.56 | 2.18 | 1.63 | 0.103 |
| Age (Medium-aged Adults): Chronic Health Condition (Yes) | -1.89 | 1.58 | -1.20 | 0.231 |
| Age (Older Adults): Chronic Health Condition (Yes) | -1.41 | 1.90 | -0.75 | 0.456 |
| Education (Low Education): Chronic Health Condition (Yes) | 2.15 | 2.78 | 0.77 | 0.439 |
| Education (Medium Education): Chronic Health Condition (Yes) | 0.73 | 1.66 | 0.44 | 0.658 |

*Note*. Reference groups: Sex (Male), Age (Young adults), Education (High Education), Chronic Health Condition (No); *b*: non-standardized regression coefficient, *SE*: standard error, t*:* *t*-value, *p*: *p*-value.

### Emotional Problems

Table S42. Results of Regression Analysis for the QoLIBRI Emotional Problems Scale

|  | *b* | *SE* | *t* | *p* |
| --- | --- | --- | --- | --- |
| Intercept | 60.35 | 1.07 | 56.59 | <0.001 |
| Sex (Female) | -1.90 | 0.76 | -2.51 | 0.012 |
| Age (Medium-aged Adults) | 15.67 | 0.88 | 17.90 | <0.001 |
| Age (Older Adults) | 28.51 | 1.03 | 27.74 | <0.001 |
| Education (Low Education) | -4.73 | 1.51 | -3.14 | 0.002 |
| Education (Medium Education) | -0.87 | 0.90 | -0.97 | 0.331 |
| Chronic Health Condition (Yes) | -5.94 | 0.76 | -7.76 | <0.001 |

*Note*. Reference groups: Sex (Male), Age (Young adults), Education (High Education), Chronic Health Condition (No); *b*: non-standardized regression coefficient, *SE*: standard error, t*:* *t*-value, *p*: *p*-value.

Table S43. Results of Second-Order Regression Model for the QoLIBRI Emotional Problems Scale

|  | *b* | *SE* | *t* | *p* |
| --- | --- | --- | --- | --- |
| Intercept | 64.17 | 2.03 | 31.64 | <0.001 |
| Sex (Female) | -5.25 | 2.05 | -2.56 | 0.010 |
| Age (Medium-aged Adults) | 12.93 | 2.23 | 5.79 | <0.001 |
| Age (Older Adults) | 22.33 | 2.52 | 8.85 | <0.001 |
| Education (Low Education) | -6.22 | 3.51 | -1.77 | 0.076 |
| Education (Medium Education) | -2.42 | 2.12 | -1.14 | 0.255 |
| Chronic Health Condition (Yes) | -9.65 | 2.15 | -4.49 | <0.001 |
| Sex (Female): Age (Medium-aged Adults) | 3.97 | 1.78 | 2.23 | 0.025 |
| Sex (Female): Age (Older Adults) | 2.95 | 2.09 | 1.41 | 0.158 |
| Sex (Female): Education (Low Education) | -1.15 | 3.07 | -0.37 | 0.709 |
| Sex (Female): Education (Medium Education) | 0.65 | 1.87 | 0.35 | 0.728 |
| Sex (Female): Chronic Health Condition (Yes) | 0.93 | 1.56 | 0.59 | 0.553 |
| Age (Medium-aged Adults): Education (Low Education) | -2.11 | 3.54 | -0.60 | 0.551 |
| Age (Older Adults): Education (Low Education) | -0.54 | 4.28 | -0.13 | 0.900 |
| Age (Medium-aged Adults): Education (Medium Education) | -0.34 | 2.21 | -0.16 | 0.877 |
| Age (Older Adults): Education (Medium Education) | 2.27 | 2.44 | 0.93 | 0.353 |
| Age (Medium-aged Adults): Chronic Health Condition (Yes) | 1.91 | 1.77 | 1.08 | 0.279 |
| Age (Older Adults): Chronic Health Condition (Yes) | 5.15 | 2.13 | 2.42 | 0.016 |
| Education (Low Education): Chronic Health Condition (Yes) | 5.08 | 3.12 | 1.63 | 0.104 |
| Education (Medium Education): Chronic Health Condition (Yes) | 1.25 | 1.86 | 0.68 | 0.500 |

*Note*. Reference groups: Sex (Male), Age (Young adults), Education (High Education), Chronic Health Condition (No); *b*: non-standardized regression coefficient, *SE*: standard error, t*:* *t*-value, *p*: *p*-value.

### Physical Problems

Table S44. Results of Regression Analysis for the QoLIBRI Physical Problems Scale

|  | *b* | *SE* | *t* | *p* |
| --- | --- | --- | --- | --- |
| Intercept | 68.29 | 1.04 | 65.75 | <0.001 |
| Sex (Female) | -1.57 | 0.74 | -2.12 | 0.034 |
| Age (Medium-aged Adults) | 9.71 | 0.85 | 11.39 | <0.001 |
| Age (Older Adults) | 13.85 | 1.00 | 13.84 | <0.001 |
| Education (Low Education) | -5.14 | 1.47 | -3.50 | <0.001 |
| Education (Medium Education) | -2.98 | 0.88 | -3.40 | 0.001 |
| Chronic Health Condition (Yes) | -12.34 | 0.74 | -16.57 | <0.001 |

*Note*. Reference groups: Sex (Male), Age (Young adults), Education (High Education), Chronic Health Condition (No); *b*: non-standardized regression coefficient, *SE*: standard error, t*:* *t*-value, *p*: *p*-value.

Table S45. Results of Second-Order Regression Model for the QOLIBRI Physical Problems Scale

|  | *b* | *SE* | *t* | *p* |
| --- | --- | --- | --- | --- |
| Intercept | 67.97 | 1.97 | 34.44 | <0.001 |
| Sex (Female) | -1.60 | 1.99 | -0.81 | 0.421 |
| Age (Medium-aged Adults) | 10.84 | 2.17 | 4.99 | <0.001 |
| Age (Older Adults) | 13.99 | 2.46 | 5.69 | <0.001 |
| Education (Low Education) | -4.94 | 3.41 | -1.45 | 0.148 |
| Education (Medium Education) | -4.68 | 2.07 | -2.26 | 0.024 |
| Chronic Health Condition (Yes) | -8.94 | 2.09 | -4.27 | <0.001 |
| Sex (Female): Age (Medium-aged Adults) | 1.64 | 1.73 | 0.95 | 0.343 |
| Sex (Female): Age (Older Adults) | -0.28 | 2.03 | -0.14 | 0.890 |
| Sex (Female): Education (Low Education) | -4.11 | 2.99 | -1.37 | 0.169 |
| Sex (Female): Education (Medium Education) | 1.15 | 1.82 | 0.63 | 0.528 |
| Sex (Female): Chronic Health Condition (Yes) | -2.00 | 1.52 | -1.31 | 0.190 |
| Age (Medium-aged Adults): Education (Low Education) | -2.84 | 3.45 | -0.82 | 0.410 |
| Age (Older Adults): Education (Low Education) | 4.07 | 4.17 | 0.98 | 0.329 |
| Age (Medium-aged Adults): Education (Medium Education) | 0.71 | 2.16 | 0.33 | 0.742 |
| Age (Older Adults): Education (Medium Education) | 2.33 | 2.38 | 0.98 | 0.326 |
| Age (Medium-aged Adults): Chronic Health Condition (Yes) | -4.76 | 1.72 | -2.77 | 0.006 |
| Age (Older Adults): Chronic Health Condition (Yes) | -3.99 | 2.07 | -1.93 | 0.054 |
| Education (Low Education): Chronic Health Condition (Yes) | 4.07 | 3.04 | 1.34 | 0.181 |
| Education (Medium Education): Chronic Health Condition (Yes) | 0.42 | 1.81 | 0.23 | 0.816 |

*Note*. Reference groups: Sex (Male), Age (Young adults), Education (High Education), Chronic Health Condition (No); *b*: non-standardized regression coefficient, *SE*: standard error, t*:* *t*-value, *p*: *p*-value.

### QoLIBRI Total Score

Table S46. Results of Second-Order Regression Model for the QoLIBRI Total Score

|  | *b* | *SE* | *t* | *p* |
| --- | --- | --- | --- | --- |
| (Intercept) | 66.99 | 1.30 | 51.35 | <0.001 |
| Sex (Female) | -2.21 | 1.32 | -1.68 | 0.093 |
| Age (Medium-aged Adults) | 6.81 | 1.44 | 4.74 | <0.001 |
| Age (Older Adults) | 12.63 | 1.62 | 7.78 | <0.001 |
| Education (Low Education) | -10.48 | 2.26 | -4.65 | <0.001 |
| Education (Medium Education) | -3.45 | 1.37 | -2.52 | 0.012 |
| Chronic Health Condition (Yes) | -7.01 | 1.38 | -5.07 | <0.001 |
| Sex (Female): Age (Medium-aged Adults) | 1.52 | 1.14 | 1.33 | 0.185 |
| Sex (Female): Age (Older Adults) | 0.76 | 1.34 | 0.56 | 0.573 |
| Sex (Female): Education (Low Education) | 1.68 | 1.98 | 0.85 | 0.397 |
| Sex (Female): Education (Medium Education) | -0.07 | 1.20 | -0.06 | 0.953 |
| Sex (Female): Chronic Health Condition (Yes) | 1.03 | 1.01 | 1.03 | 0.304 |
| Age (Medium-aged Adults): Education (Low Education) | 2.18 | 2.28 | 0.95 | 0.340 |
| Age (Older Adults): Education (Low Education) | 3.86 | 2.75 | 1.40 | 0.161 |
| Age (Medium-aged Adults): Education (Medium Education) | 0.19 | 1.43 | 0.14 | 0.892 |
| Age (Older Adults): Education (Medium Education) | 2.73 | 1.57 | 1.74 | 0.083 |
| Age (Medium-aged Adults): Chronic Health Condition (Yes) | -1.42 | 1.14 | -1.25 | 0.213 |
| Age (Older Adults): Chronic Health Condition (Yes) | -0.75 | 1.37 | -0.55 | 0.586 |
| Education (Low Education): Chronic Health Condition (Yes) | 2.52 | 2.01 | 1.26 | 0.209 |
| Education (Medium Education): Chronic Health Condition (Yes) | 1.10 | 1.20 | 0.92 | 0.358 |

*Note*. Reference groups: Sex (Male), Age (Young adults), Education (High Education), Chronic Health Condition (No); *b*: non-standardized regression coefficient, *SE*: standard error, t*:* *t*-value, *p*: *p*-value.

### QoLIBRI-OS Total Score

Table S47. Results of Second-Order Regression Model for the QOLIBRI-OS Total Score

|  | *b* | *SE* | *t* | *p* |
| --- | --- | --- | --- | --- |
| Intercept | 67.09 | 1.69 | 39.80 | <0.001 |
| Sex (Female) | -1.05 | 1.70 | -0.62 | 0.536 |
| Age (Medium-aged Adults) | 2.91 | 1.85 | 1.57 | 0.117 |
| Age (Older Adults) | 6.73 | 2.10 | 3.21 | 0.001 |
| Education (Low Education) | -16.33 | 2.91 | -5.60 | <0.001 |
| Education (Medium Education) | -4.82 | 1.76 | -2.73 | 0.006 |
| Chronic Health Condition (Yes) | -7.18 | 1.79 | -4.02 | <0.001 |
| Sex (Female): Age (Medium-aged Adults) | 0.79 | 1.48 | 0.54 | 0.591 |
| Sex (Female): Age (Older Adults) | -0.12 | 1.74 | -0.07 | 0.945 |
| Sex (Female): Education (Low Education) | 4.13 | 2.56 | 1.62 | 0.106 |
| Sex (Female): Education (Medium Education) | -0.31 | 1.56 | -0.20 | 0.843 |
| Sex (Female): Chronic Health Condition (Yes) | 1.39 | 1.30 | 1.07 | 0.285 |
| Age (Medium-aged Adults): Education (Low Education) | 5.52 | 2.94 | 1.87 | 0.061 |
| Age (Older Adults): Education (Low Education) | 5.46 | 3.56 | 1.54 | 0.125 |
| Age (Medium-aged Adults): Education (Medium Education) | 0.22 | 1.84 | 0.12 | 0.903 |
| Age (Older Adults): Education (Medium Education) | 3.86 | 2.03 | 1.90 | 0.058 |
| Age (Medium-aged Adults): Chronic Health Condition (Yes) | -4.61 | 1.47 | -3.14 | 0.002 |
| Age (Older Adults): Chronic Health Condition (Yes) | -3.37 | 1.77 | -1.90 | 0.057 |
| Education (Low Education): Chronic Health Condition (Yes) | 4.00 | 2.59 | 1.54 | 0.123 |
| Education (Medium Education): Chronic Health Condition (Yes) | 1.46 | 1.54 | 0.94 | 0.346 |

*Note*. Reference groups: Sex (Male), Age (Young adults), Education (High Education), Chronic Health Condition (No); *b*: non-standardized regression coefficient, *SE*: standard error, t*:* *t*-value, *p*: *p*-value.
